# An ongoing struggle: a mixed-method systematic review of interventions, barriers and facilitators to achieving optimal self-care by children and young people with Type 1 Diabetes in educational settings

**DOI:** 10.1186/1471-2431-14-228

**Published:** 2014-09-12

**Authors:** Deborah Edwards, Jane Noyes, Lesley Lowes, Llinos Haf Spencer, John W Gregory

**Affiliations:** School of Healthcare Sciences College of Biomedical and Life Sciences, Cardiff University, Cardiff, UK; School of Social Sciences, Bangor University, Bangor, LL57 2EF UK; School of Healthcare Sciences, College of Health and Behavioural Sciences, Bangor University, Bangor, UK; Department of Child Health, Wales School of Medicine, Cardiff University, Cardiff, UK

**Keywords:** Systematic review, Diabetes Type 1, Children, Adolescent, Young Person, Educational setting, School, University, College, School nurse

## Abstract

**Background:**

Type 1 diabetes occurs more frequently in younger children who are often pre-school age and enter the education system with diabetes-related support needs that evolve over time. It is important that children are supported to optimally manage their diet, exercise, blood glucose monitoring and insulin regime at school. Young people self-manage at college/university.

**Method:**

Theory-informed mixed-method systematic review to determine intervention effectiveness and synthesise child/parent/professional views of barriers and facilitators to achieving optimal diabetes self-care and management for children and young people age 3–25 years in educational settings.

**Results:**

Eleven intervention and 55 views studies were included. Meta-analysis was not possible. Study foci broadly matched school diabetes guidance. Intervention studies were limited to specific contexts with mostly high risk of bias. Views studies were mostly moderate quality with common transferrable findings.

Health plans, and school nurse support (various types) were effective. Telemedicine in school was effective for individual case management. Most educational interventions to increase knowledge and confidence of children or school staff had significant short-term effects but longer follow-up is required. Children, parents and staff said they struggled with many common structural, organisational, educational and attitudinal school barriers. Aspects of school guidance had not been generally implemented (e.g. individual health plans). Children recognized and appreciated school staff who were trained and confident in supporting diabetes management.

Research with college/university students was lacking. Campus-based college/university student support significantly improved knowledge, attitudes and diabetes self-care. Self-management was easier for students who juggled diabetes-management with student lifestyle, such as adopting strategies to manage alcohol consumption.

**Conclusion:**

This novel mixed-method systematic review is the first to integrate intervention effectiveness with views of children/parents/professionals mapped against school diabetes guidelines. Diabetes management could be generally improved by fully implementing and auditing guideline impact. Evidence is limited by quality and there are gaps in knowledge of what works. Telemedicine between healthcare providers and schools, and school nurse support for children is effective in specific contexts, but not all education systems employ onsite nurses. More innovative and sustainable solutions and robust evaluations are required. Comprehensive lifestyle approaches for college/university students warrant further development and evaluation.

**Electronic supplementary material:**

The online version of this article (doi:10.1186/1471-2431-14-228) contains supplementary material, which is available to authorized users.

## Background

Type 1 diabetes (T1D) now occurs more frequently in younger children who are often pre-school age and enter the education system with specific support needs to optimally manage their blood glucose and insulin regime [1]. It is predicted that there will be a rise in childhood T1D across all ages in Europe over the next 20 years [2]. In the United States (US), approximately 13,000 new cases are diagnosed annually in children with about 15,000 young people under 19 years of age living with T1D [3].

In order to minimise the risk of developing long-term complications it is important that every child and young person with T1D receives appropriate care from diagnosis, and that good metabolic control is maintained [4]. Most children age 4 to 11 years are dependent on adults for their T1D care and for many, a large part of every day is spent in educational, or early years settings. It is important that systems are in place so that children and young people feel comfortable in educational settings and confident to manage their T1D. To optimize the child’s T1D management, school personnel must be knowledgeable about T1D care issues and provide an environment that promotes safety and optimal T1D management. The child with T1D should be able to participate fully in all school activities while performing blood glucose testing, eating appropriately, and administering insulin as needed. Young people attending college/university often live away from their families and need to be able to independently self-manage their T1D.

### Why is the review needed?

Two recent narrative reviews [5, 6] have focused on T1D and school and both have methodological limitations. Wodrich *et al.*[6] did not use systematic processes or report the characteristics and designs of studies. Tolbert [5] only used the keywords type 1 diabetes, school and management to retrieve 10 quantitative descriptive surveys and 1 mixed-method study. No attempt was made to determine study quality in either review. Although these reviews provide useful background context, neither provide a trusted source of synthesized evidence to inform decision-making and policy and practice development.

It is important that a child or young person’s T1D should be managed effectively in educational settings in order to ensure optimal glycaemic control. In contrast to the previous reviews, we sought to conduct a policy-informed mixed-method systematic review that utilized a comprehensive and systematic search strategy and assessed the methodological quality of the included studies. Findings from the review were then used to inform intervention development in a large United Kingdom (UK) Government funded study [7]. The objectives were:

- To determine the effectiveness of interventions across all outcomes conducted with children, young people and school personnel to optimize T1D care and management in educational settings,
- To explore the attitudes and experiences of children and young people with T1D and those involved with their care and management to identify the barriers and facilitators to achieving optimal T1D management educational settings, and
- To conduct an overarching synthesis to determine the extent to which interventions to optimize T1D care and management in educational settings addressed the barriers, and built on the facilitators, to optimal care identified by children, young people, parents and school personnel.

### Conceptual framework

In the UK [8–18] and US [19–22], a number of key guidelines set out the components of safe and optimal T1D care at school. International clinical practice consensus guidance has also been developed by the International Society for Pediatric and Adolescent Diabetes (ISPAD) [14]. The development of T1D medical management plans that address specific needs of the child, specific guidance on general T1D management, and training needs of all those involved in supporting the child with T1D at school are common across all guidelines. We extracted key elements of best practice for children’s T1D management in schools and used this as a conceptual framework to guide analysis and interpretation of evidence (Table 1).

**Table 1:** **Common elements of effective diabetes management in school**

Policies and Guidelines used in the UK[8–18] – including European guidelines.	Policies and Guidelines US[19–23]
**Assembling school health care plans**	
An individualised diabetes medical management plan should be agreed by the parent/guardian, school, and the student’s Children and Young Persons Specialist Diabetes team [12] and updated on a regular basis [11].	A Diabetes Medical Management Plan (DMMP) should be developed by the student’s personal diabetes health care team with input from the parent/guardian [19, 22] along with specific Individualized Health Care Plans (IMP) and Emergency Care Plans (EMP) [20].
**Checking blood glucose during the school day**	
To provide and clean and safe environment [11].	A location in the school that provides privacy during blood glucose monitoring [19, 22].
Suitable location to check blood glucose [9].	Permission for the student to check his or her blood glucose level and take appropriate action to treat hypoglycaemia in the classroom or anywhere the student is in conjunction with a school activity, if indicated in the student’s DMMP [19, 20, 22].
**Accessibility and storage of supplies**	
Provision of fridge space for spare supplies of insulin [11].	Permission for self-sufficient and capable students to carry equipment, supplies, medication, and snacks; to perform diabetes management tasks [19, 22].
Provide correct storage of supplies where necessary [11].	
Diabetes supplies and equipment (for example, glucogel, glucose drinks and some complex carbohydrate to treat hypoglycaemic episodes) should be accessible to the student at all times [8, 9].	An appropriate location for insulin and/or glucagon storage, if necessary [19, 22].
Parents and, where appropriate, school nurses and other carers should have access to glucagon for subcutaneous or intramuscular use in an emergency, especially when there is a high risk of severe hypoglycaemia [17].	The parents/guardian should supply the school with a glucagon emergency kit [20, 23].
Parents and, where appropriate, school nurses and other carers should be offered education on the administration of glucagon [17].	The school nurse and/or trained diabetes personnel must know where the kit is stored and have access to it at all times [20, 23].
	An appropriate location glucagon storage, if necessary [19, 22].
The provision of emergency supply boxes [11].	The parents/guardian must provide an emergency supply kit for use in the event of natural disasters or emergencies when students need to stay at school [20].
Hyperglycemia remedies should always be readily available at school [18].	
**Administering insulin during the school day**	
Provide and clean and safe environment [11].	The school nurse and/or trained diabetes personnel should assist with insulin administration in accordance with the student’s health care plans and education plans [20].
Suitable, private location to manage injections [9].	A location in the school that provides privacy during insulin administration, [19, 22].
	Accessibility to scheduled insulin at times set out in the student’s DMMP as well as immediate accessibility to treatment for hyperglycemia including insulin administration as set out by the student’s DMMP [19, 22].
**Accessibility of and participation in physical education in schools**	
Schools should allow children and young people with diabetes to manage their diabetes according to their chosen management form and to take part in the full range of school activities [12].	Students with diabetes should participate fully in physical education classes and team or individual sports [20].
Staff in charge of physical education or other physical activity sessions should be aware of the need for them to have glucose tablets or a sugary drink to hand [9].	Physical education teachers and sports coaches must be able to recognize the symptoms of hypoglycemia and be prepared to call for help with a hypoglycemia emergency [20].
**Food and dietary management**	
To give permission for child/young person to eat whenever required [11].	School nurse and back-up trained school personnel responsible for the student who will know the schedule of the student’s meals and snacks and work with the parent/guardian to coordinate this schedule with that of the other students as closely as possible [19, 22].
Children and young people with diabetes need to be allowed to eat regularly during the day. This may include eating snacks during class-time or prior to exercise. Schools may need to make special arrangements for them if the school has staggered lunchtimes [9].	Permission for the student to eat a snack anywhere, including the classroom or the school bus, if necessary to prevent or treat hypoglycemia [19, 22].
Snacks should be available during the school day [18].	The food service manager or staff and/or the school nurse should provide the carb content of foods to the parents/guardian and the student [20].
	Information on serving size and caloric, carbohydrate, and fat content of foods served in the school [19, 22].
**Planning for special events, field trips, and extracurricular activities**	
Pupils with diabetes must not be excluded from day or residential visits on the grounds of their condition [12].	Full participation in all field trips, with coverage provided by trained diabetes personnel [19].
Information should be readily available from the paediatric diabetes specialist nurse on the inclusion of children and young people with diabetes on school trips [11].	The school nurse or trained diabetes personnel should accompany the student with diabetes on field trips [20].
	Parental attendance at field trips should never be a prerequisite for participation by students with diabetes [20].
	Full participation in all school-sponsored activities, with coverage provided by trained diabetes personnel [19, 22].
	The school nurse or trained diabetes personnel should be available during school-sponsored extracurricular activities that take place outside of school hours [20].
**Flexible accommodation for exams and tests**	
	Permission for the student to use the restroom and have access to fluids (i.e., water) as necessary [19, 22].
	Alternative times and arrangements for academic exams if the student is experiencing hypoglycaemia or hyperglycaemia [20].
**Dealing with emotional and social issues**	
	The student’s personal diabetes health care team and school health team must be aware of emotional and behavioral issues and refer students with diabetes and their families for counseling and support as needed [20].
**Assisting the student with performing diabetes care tasks** **(Blood glucose monitoring, insulin and glucagon administration, and urine or blood ketone testing)**	
Support for blood glucose monitoring and guidance on the interpretation of blood glucose results and any subsequent action [8, 9].	Assignment of diabetes care tasks, must take into account State laws that may be relevant in determining which tasks are performed by trained diabetes personnel [20].
Support of administration of insulin including treatment changes and a suitable location [8, 9].	The school nurse is the most appropriate person in the school setting to provide care for a student with diabetes [20].
	The School nurse and back-up trained school personnel who can check blood glucose and ketones and administer insulin, glucagon, and other medications as indicated by the student’s DMM [19, 22].
	Permission for the student to see the school nurse and other trained school personnel upon request [19, 22].
	Permission to miss school without consequences for illness and required medical appointments to monitor the student’s diabetes management. This should be an excused absence with a doctor’s note, if required by usual school policy [19, 22].
**Diabetes education and training of school nurses and school personnel**	
Staff in schools should receive appropriate and consistent training, advice and support from health services and children’s diabetes specialist service [11].	All school personnel - Level 1. Diabetes Overview and How to Recognize and Respond to an Emergency Situation [19, 20, 22].
Education about diabetes must be provided to teachers and other school personnel, including school receptionists, PE teachers and school nurses, on a regular basis [12].	School personnel who have responsibility for the student with diabetes throughout the school day (e.g., classroom, physical education, music, and art teachers and other personnel such as lunchroom staff, coaches, and bus drivers).- Level 2 Diabetes Basics and What to Do in an Emergency Situation [19, 20, 22].
Children and young people, their parents, schoolteachers and other carers should be offered education about the recognition and management of hypoglycaemia [17].	School staff members designated as trained diabetes personnel who will perform or assist the student with diabetes care tasks when allowed by State law - Level 3. General and Student-Specific Diabetes Care Tasks [19, 20, 22].
Staff members need an appropriate level of diabetes education, and this should be relevant to activities that take place on the premises as well as those associated with participation in school trips and camps [24].	
It is important that when staff agree to administer blood glucose tests or insulin injections, they should be trained by an appropriate health professional [17].	School nurses need to update their diabetes knowledge regularly and have their competencies checked on a regular basis [21].
	Training of nonmedical school personnel to perform diabetes care duties is essential and should be facilitated by a diabetes-trained health care professional such as the school nurse or a certified diabetes educator [20].
When staff agree to administer blood glucose tests or insulin injections, they should be trained by an appropriate health professional [8, 9].	Opportunities for the appropriate level of ongoing training and diabetes education for the school nurse [19, 22].
**Recognizing and treating hypoglycemia**	
Ability to recognise and manage hypoglycemia [8–16].	Early recognition of hypoglycemia symptoms and prompt treatment [20].
	All school personnel who have responsibility for the student with diabetes should receive a copy of the Hypogycemia Emergency Care [20].
**Recognizing and treating hyperglycemia**	
Awareness by school staff of the signs of hyperglycaemia [8–16].	Hyperglycemia needs to be recognized and treated in accordance with the student’s DMMP [20].
	All school personnel who have responsibility for the student with diabetes should receive a copy of the Hyperglycemia Emergency Care Plan and be prepared to recognize and respond to the signs and symptoms of hyperglycemia [20]. Supervision until appropriate treatment has been administered [19, 22].
**Communication between school health personnel and diabetes healthcare providers**	
None identified	None identified
**Self-care and management at college/university**	
None identified	None identified

## Methods

### Review design

**Figure 1:**
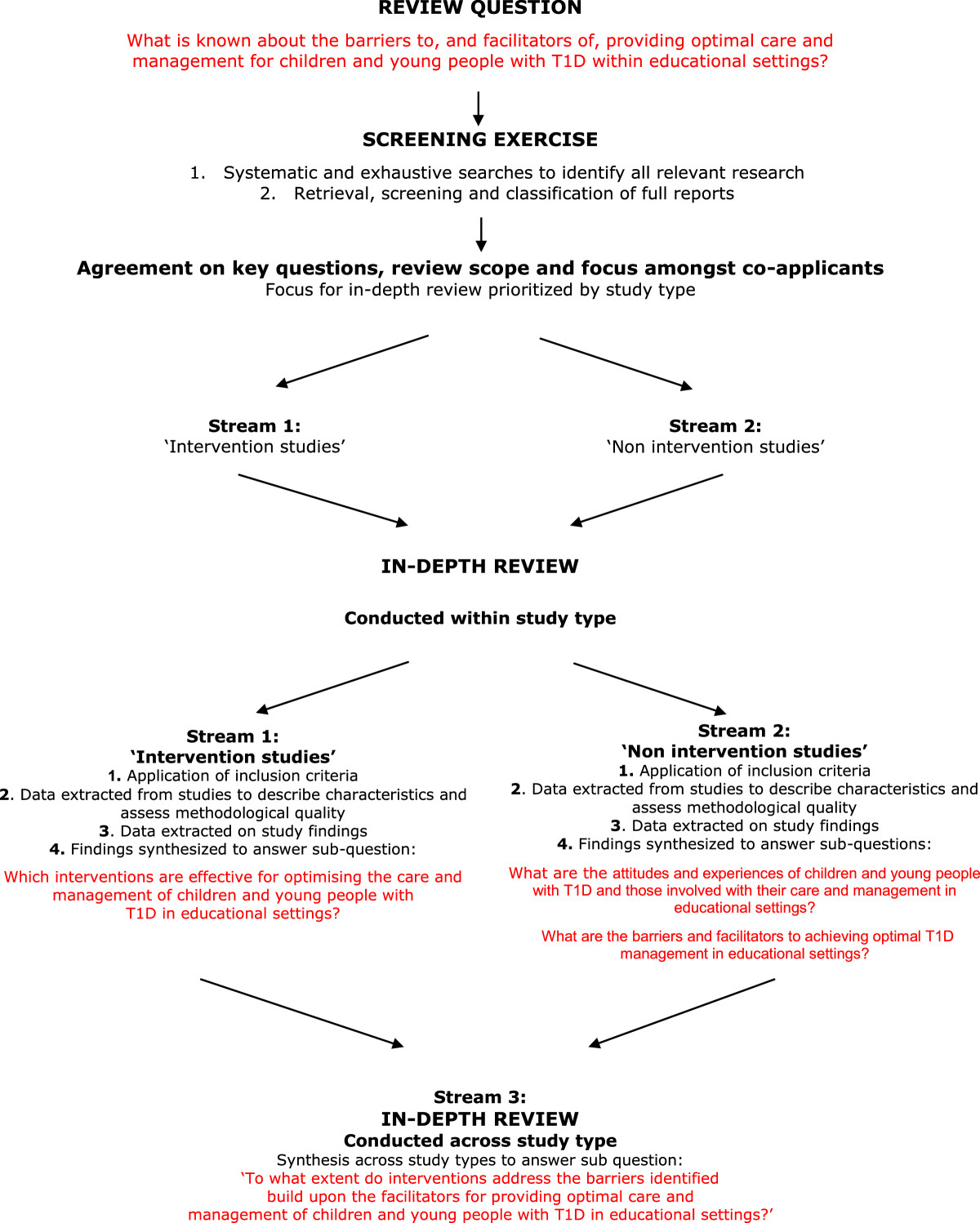
**Mixed-methods review design.**

We conducted a mixed-method systematic review. The design was informed by mixed-method synthesis methods developed by the Evidence for Policy and Practice Information (EPPI) Centre [25, 26] and is shown in Figure 1. We followed Cochrane Effective Practice and Organisation of Care Guidance on the inclusion of more diverse quantitative study designs to determine the effectiveness of interventions as our initial scoping review has identified few randomized controlled trials [27]. The EPPI ‘mixed-methods’ triangulation approach maps evidence from effectiveness studies (Stream1: quantitative data) with evidence from studies reporting the attitudes and experiences of participants (Stream 2: non intervention studies including surveys and qualitative studies). We then conducted an overarching narrative synthesis from streams 1 and 2 to determine the extent to which interventions to optimize T1D care and management in educational settings addressed the barriers, and built on the facilitators, identified by children, parents and teachers. The quantitative component of the review (stream 1) adhered as far as possible to PRISMA reporting guidelines (http://www.prisma-statement.org). We developed a detailed protocol which is not publically available.

### Search methods

The search strategy is summarised within a modified Setting, Population/People/Perspective, Intervention/Issue of Interest, Comparison, Evaulation (SPICE) [28] table (see Table 2). The search terms included medical subject headings (MeSH) and ‘free text’ terms in combination and was adapted according to the particular database. A single search was used for both stages of the review with no methodological restrictions (for a sample of searches see Additional file 1). The databases searched for relevant studies were: CINAHL, MEDLINE, Scopus, British Nursing Index, Cochrane Library, EMBASE, PsychINFO and Web Of Science. In addition, reference lists of retrieved papers and published reviews were searched and unpicked for potentially relevant papers. References were managed using Endnote X1.

**Table 2:** **Search terms presented with the SPICE Framework**

Quantitative review of the strategies and/or interventions that are conducted within an educational setting that seek to improve the care of children and young people with type 1 diabetes				
Setting	Population	Intervention and issues of interest	Comparison	Evaluation
Educational Setting in any country	Children/Young People with type 1 diabetes	All interventions to promote optimal management diabetes in school settings	Any comparison of interest including usual care	Blood Glucose Monitoring
12^th^/twelfth grade	3- 18 years pre school or education	Educational		Glyc*mic control
6^th^/sixth grade	18 – 30 in higher education	Psychosocial		Blood Glucose Monitoring
College	**School-aged children**	Medical		Blood Glucose Levels
Diabetes Camp	P*diatric	Nursing		Self Monitoring Blood Glucose
Institute	Child$	Psychotherapeutic		Blood glucose testing
Junior High	Adolescen$	Secondary issues to include programme theory and service delivery.		BG
Kindergarden	Young person$			Metabolic glyc*mic control
Kindergarten	Young people			Glucose control
Nursery	Young patients			SMBG
Polytechnic	Young women			Self monitoring
Pre School	Young men			Self regulation
School	Young adult$			Metabolic control
School Camp	Youngsters			Blood sugars
Summer camp	Youth			Hypos
University	Year old$			Hyperglyc*mia
	Teen$			Low blood sugar
	Years of age			Hyperglyaemia
	Juvenile			High blood sugar
	Pube$			
	Adult {and type 1 and/ , ages 16, 17, 18)			
				HbA1c
	**Condition**			Glycos*lated H*moglobin
	Diabetes			Glycated H*mogloblin
	Diabetes Mellitus			GHb
	Diabetes Mellitus , Type 1			H*moglobin A1c
	Diabetic			HbA1c
	Diabetic patients			Auto controlling gly*emia
	Diabetic control			
	Type 1 or type l			Insulin Management
	DM			Insulin injections
	IDDM			Insulin sensitivity
	Insulin dependent diabetes mellitus			Insulin adjustment
	Sudden onset diabetes mellitus			Insulin replacement
	Auto immune diabetes mellitus			Hypoglycemic Agents
	Insulin deficient diabetes mellitus			
	Diabetes insipidus			Dietary behaviour
	Early diabetes mellitus			Nutrition
	Labile diabetes mellitus			Eating patterns
	T1D			Eating behavio*r
	Juvenile Diabetes			Carbohydrates
				Carbs
				CHO
				Snacks
				Snacking
				Carbohydrate Counting
				Carb Counting
Qualitative synthesis of the facilitators and barriers to managing type 1 diabetes within an educational setting for children and young people with type 1 diabetes and those involved with their care				
**Setting**	**Perspective/People**	**Issues of Interest**	**Comparison**	**Evaluation**
Educational Setting in any country	Children/Young People with type 1 diabetes	Facilitators/Barriers to:	Compare children with parents/professionals	Management
12^th^/twelfth grade	3 - 18 years preschool or formal education		Family	Patient care management
6^th^/sixth grade	18 – 30 post compulsory education	Problems/Support	Families	Management skills
College	**School-aged children**	Knowledge of	Siblings	Self-management behaviours
Diabetes Camp	P*diatric	Attitudes to	Brothers	
Institute	Child$	Experiences of	Sisters	Self-management
Junior High	Adolescen$	Knowledge	Parents	Self-care
Kindergarden	Young person$	Attitudes	Mother	Care
Kindergarten	Young people	Training of staff	Father	Self-efficacy
Nursery	Young patients	Compliance	Grandparents	Self Regualt$
Polytechnic	Young women	Behaviours	Peers	Self monitor$
Pre School	Young men	Knowledge	School Nurses	Self manage$
School	Young adult$	Attitudes	School Staff	Self Adheren$
School Camp	Youngsters	Training of staff	Teachers	Medical Management
Summer camp	Youth	Compliance	School Psychologists	Health care routines
University	Year old$	Behaviours	School Counsellors	Health related quality life
	Teen$	Needs	School Nurses	
	Years of age	Perceptions	School Health Professionals	
	Juvenile	Concerns	School personnel	
	Pube$	Practices	School Administrators	
	Adult {and type 1 and/, ages 16, 17, 18)	Expectations	Coaches	
			Teaching assistants	
			Learning support assistant/LSA	
	**Condition**			
	Diabetes			
	Diabetes Mellitus			
	Diabetes Mellitus, Type 1			
	Diabetic			
	Diabetic patients			
	Diabetic control			
	Type 1 or type l			
	DM			
	IDDM			
	Insulin dependent diabetes mellitus			
	Sudden onset diabetes mellitus			
	Auto immune diabetes mellitus			
	insulin deficient diabetes mellitus			
	Diabetes insipidus			
	Early diabetes mellitus			
	Labile diabetes mellitus			
	T1D			
	Juvenile Diabetes			

### Inclusion/exclusion criteria

Studies were included if they focused on children and young people with T1D within an educational setting and included those 3–16 years in preschool or formal education and those 16–25 in post compulsory education. In addition, studies including or focusing on parents, peers, educational setting personnel and health professionals that related to this age group were included. Restrictions were not applied in terms of research design or methods. Unpublished data were not sought from authors. All studies published in the preceding 15 years were included (January 1996-July 2011) that were conducted in any country and published in English in peer-reviewed scientific journal. A 15 year window was selected to capture a reasonably contemporary context. Studies were excluded if there was no before and after measures (stream 1) and if the study did not directly report the views of children and young people, parents, peers, professionals (stream 2).

### Screening

All studies identified were assessed for relevance by DE and LS to the review based on the title and abstract. For studies that appeared to meet the inclusion criteria, or in cases when a definite decision could not be made based on the title and/or abstract alone, the full paper was obtained for detailed assessment by two researchers against the inclusion criteria. Any disagreement was resolved by consultation with a third independent reviewer (JN).

### Search outcome

**Figure 2:**
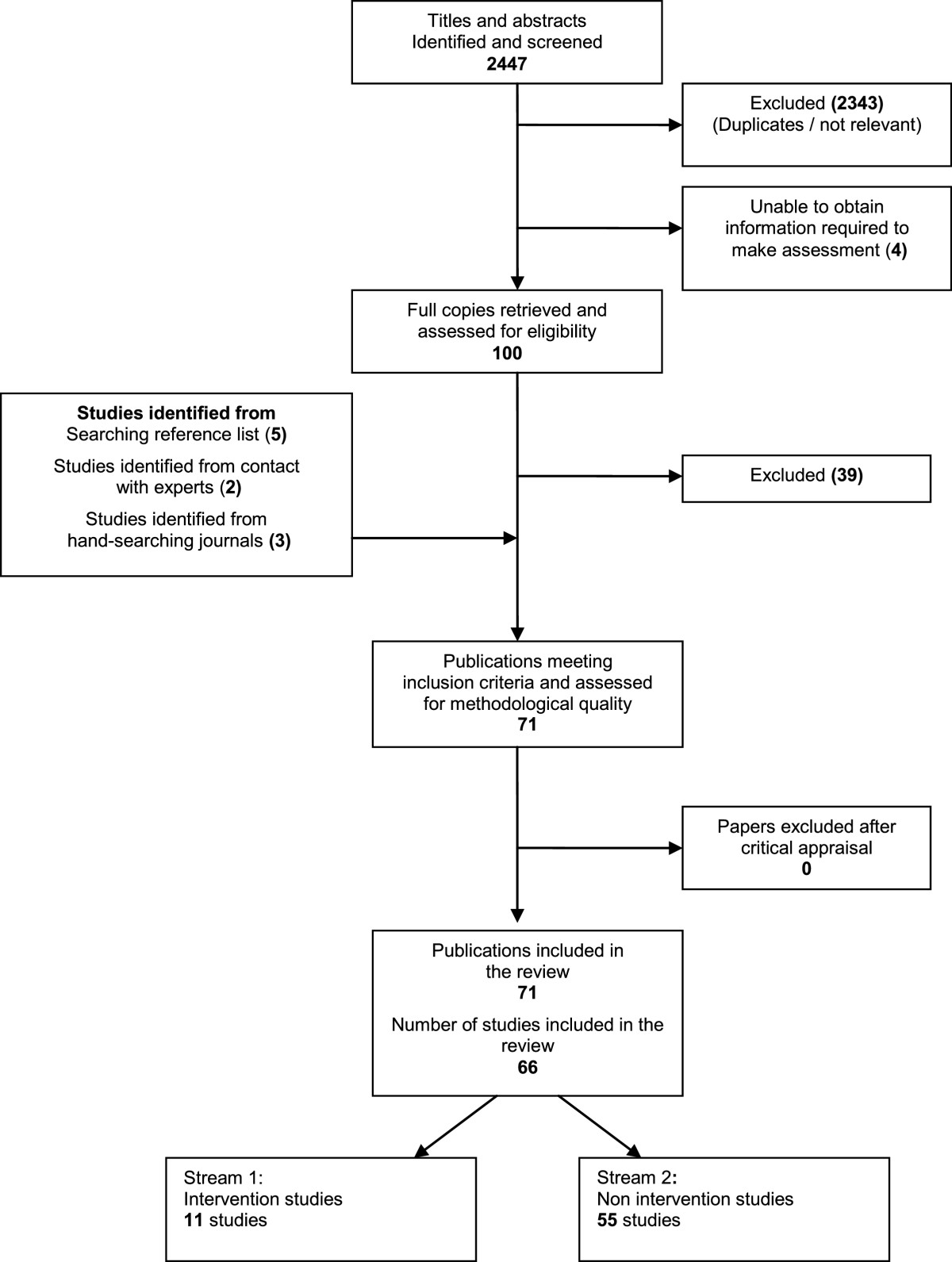
**Flow chart through study selection process.**

Figure 2 shows the flow of papers at each stage. A total of 71 papers reporting 66 studies were included.

### Quality assessment

For stream 1 (intervention studies), randomised intervention studies were assessed on criteria developed by Kirk *et al*. [29]. A summary of the quality assessment is provided in Table 3.

**Table 3:** **Quality of randomised intervention studies**

Author/s Country	Randomisation	Blinding	Sample size	Comparability of groups at baseline	Length of follow up	ITT	Risk of Bias
	Concealment		Use of power calculation		Attrition		
**Children and young people with T1D at school settings**							
Nguyen *et al.*[30] US	Unclear	Not applicable	18	Yes	3 months	Not reported	Unclear
	Unclear		No		2 dropped out of control group		
Izquierdo *et al.*[31] US	Unclear	Not applicable	41	Apart from mean body mass index which was lower in the intervention group	1 Year	Not reported	Unclear
	Unclear		No		Not reported		
**School personnel working with children and young people with T1D**							
Husband *et al.*[32] Canada	Unclear	Not applicable	44	Yes	7 weeks	Not reported	Unclear
	Unclear		No		37/44 completed (84%)		

Key aspects of quality for non randomised intervention studies in stream 1 were based on the work of Deek *et al.*[33] (see p39 of the Centre for Reviews and Dissemination, University of York guidance on undertaking reviews in health care [34] ). A summary of the quality assessment is provided in Table 4.

**Table 4:** **Study characteristics and quality appraisal for intervention studies (Stream 1)**

Study/Country	Design	Participant details	Age (years)	Quality appraisal
		Provider of intervention		
**Children and young people with T1D at school settings**				
Izquierdo *et al.*[31] US	RCT – 2 arms	25 schools with 41 children randomised	Target range: Kindergarten to 8^th^ grade (≤13 years)	See Table 3
		Intervention (n = 23) Usual care (n = 18)	Intervention: 9.74 ± 2.18 years	
		School nurse/PDSN	Control 10.56 ± 2.5 years	
Engelke *et al.*[35] US	Before and after study	36 children	Target range 5–19 years	ABCDEGHI
		School nurse	Actual age of sample not specified	
Nguyen *et al.*[30] US	RCT – 2 arms	36 children	I: Range 11–16 years	See Table 3
		I (n = 18)/C (n = 18)	Mean 14.0 + 1.8 years	
		School nurse/Parents	C: Range 10–17 years	
			Mean 13.3 + 1.7	
Faro *et al.*[36] US	Before and after study	27 children	Target range: Kindergarten to 6^th^ grade (≤11 years)	ABCEH
		PNP	Actual age of sample not specified	
Wdowik *et al.*[37] US	Controlled trial	31 university students	Actual range: 18 to 27 years	ABCDEHI
		I (n = 21)/C (n = 10)	Mean 22 years	
		RD/CED		
**School personnel working with children and young people with T1D**				
Husband *et al.*[32] Canada	RCT – 2 arms	44 elementary teachers	Sample characteristics of children with T1D not specified	See Table 3
		I (n = 22)/C (n = 22)		
		Diabetes researchers		
Siminerio and Koerbel [38] US	Before and after study	156 school personnel from six school districts	Not linked to specific children with T1D	ABCEF
		Diabetes educators (n = 2)		
Cunningham and Wodrich [39] US	Analog experiment (allocated)	90 regular & SE elementary teachers from 4 schools	Not linked to specific children with T1D	ABCDEFI
		Researchers		
Wodrich [40] US	Analog experiment (random assignment)	122 CE & P-S teachers from 1 university	Not linked to specific children with T1D	ABCDEFI
		Researchers		
Bullock *et al.*[41] US	Cohort study	537 school nurses	Not linked to specific children with T1D	ABCDEFHI
		Participation in an on-line CEP for T1D (n = 120)		
		Who had not participated in CEP for T1D		
		(n = 417)		
		Researchers from MDHSS/MUSSON		
Bachman and Hsueh [42] US	Program evaluation	15 school nurses	Not linked to specific children with T1D	ABCDEFHI
		Participated in an on-line CEP for T1D		
		Researchers		

**Key**: BG – Blood glucose, C – Control; CE – Continuing Education; CED - Certified Diabetes Educator; CEP - Continuing Education Program; I – Intervention; MDHSS - Missouri Department of Health and Senior Services; MUSSON - University of Missouri Sinclair School of Nursing; PDSN - Paediatric Diabetes Specialist Nurse; PEP - Paediatric Nurse Practitioner; P-S – Pre-Service; RCT – Randomised Controlled Trial; RD – Registered Dietician; SE – Special Education; T1D - Type 1 Diabetes UC – Usual Care

**Quality criteria key:** A-Clear statement of the aims of the study; B-Adequate description of the context for the study; C-Clear specification of research design and its appropriateness for the research aims; D-Reporting of clear details of the sample and method of recruitment/sampling; E-Clear description of data collection; F-Clear description data analysis provided G-Attempts made to establish rigour of data analysis; H-Discussion of ethical issues / approval details; I-Inclusion of sufficient original data to support interpretations and conclusions.

The strength of synthesized findings for stream 1 (intervention studies) was assessed using the Grading of Recommendations, Assessment, Development and Evaluation (GRADE) approach [43] where certainty of evidence is reported as being high, moderate or low/very low.

For studies in stream 2 (non-intervention studies) that used a survey design we used the checklist as designed by Rees *et al.*[44] and for qualitative studies using the appropriate checklist available from the Critical Appraisal Skills Programme (CASP) [45]. These were then incorporated with quality criteria that were adapted from Kirk *et al*. [29] to provide a summary of quality assessment and available with Table 5.

**Table 5:** **Study characteristics and quality appraisal for non-intervention studies (Stream 2)**

Study/Country	Design	Participant details	Age (years)	Quality appraisal
**Children, young people and/or parents with T1D at school settings**				
Nabors *et al.*[46] US	Interviews	105 children whilst at day and summer camp	Mean 10.11 (S.D. 2.2)	ABCDEHI
	Survey		Range 6 – 14.6	
Bodas *et al.*[47] Spain	Survey	414 children whilst at summer camps	Target range 6-16	ABCEFI
Peters *et al.*[48] US	Survey	167 children from diabetes’s clinic	Mean 12.8 (S.D. 2.5)	
	Review of clinic records		Target range 8-17	ABCDEFGHI
Lehmkuhl and Nabors [49] US	Survey	58 children whilst at summer camp	Mean 11.5 (S.D 1.0)	ABCEHI
	Pilot Study		Target range 8-14	
Tang and Ariyawansa [50] UK	Survey	11 children & 11 parents from diabetes clinics	Target range 12-16	ABCEFHI
Wang *et al.*[51] Taiwan	Interviews	2 children	Age 14/Age 15	ABCDEFGHI
Newbould *et al.*[52] UK	Interviews	26 children & 26 parents from GP practices	Mean 11.7	ABCDEFGHI
			Target range 8-15	
MacArthur [53] UK	Survey	15 children from diabetes clinics	Target range 10-16	ABCHI
Clay *et al.*[54] US	Survey	75 children & 75 parents from diabetes clinics	Mean 13.3 (S.D. 2.8)	ABCDEFGHI
			Target range 8-18	
Schwartz *et al.*[55] US	Survey	80 children & 80 parents from diabetes clinics	Target range 5-12	ABCEH
Hema *et al.*[56] US	Self completion diaries	52 children whilst at summer camp	Mean 13.02 (S.D. 2.66)/Target range 8-18	ABCDEFHI
			8-12 (n = 19)/13–18 (n = 33)	
Peyrot [57] Brazil, Denmark, Germany, Italy, Japan, The Netherlands, Spain, USA	Survey	1905 children^a^	^a^Mean 21.3 (S.D. 2.4 )/Target range 18-25	ABCDEFHI
		4099 parents^b^ part of DAWN Youth WebTalk study	^b^Mean 10.5 (S.D. 4.2)/Target range 0-16	
Carroll and Marrero [58] US	Focus groups	31 children from physicians’ offices	Mean 14.9	ABCDEFGHI
			Target range 13-18	
			13-14 (45%), 15–16 (35%), 17–18 (20%)	
Waller *et al.*[59] UK	Focus Groups	24 children & 29 parents from diabetes clinics	Mean 13.07 (S.D 1.59)	ABCDEFGHI
			Target range 11–16	
Hayes-Bohn *et al.*[60] US	Interviews	30 children & 30 parents from diabetes clinics	Mean 17.3	ABCDEFHI
			Target range 13-20	
Wagner *et al.*[61] US	Survey	58 children & 58 parents Whilst at summer camp	Mean 12 (S.D 1.9)	ABCDEFHI
			Target range 8-15	
Amillategui *et al.*[62] Spain	Survey	152 children^a^	^a^Mean 10.68 (S.D 1.92)/Target range 6-13	ABCDEFHI
		167 parents^b^ from paediatric unit s of 9 hospitals	6-9 (29%)/10–13 (71%)	
			^b^Mean 10.37 (S.D 2.15)/Target range 6-13	
			6-9 (35%)/ 10–13 (65%)	
Barnard *et al.*[63] UK	Interviews	15 children & 17 parents registered on the Roche Diagnostics insulin pump user customer database	Mean age 12.07 (S.D. 2.71)	ABCDEFGHI
			Target Range 9-17	
Low *et al.*[64] US	Interviews	18 children & 21 parents Whilst at diabetes camps & a regional paediatric endocrinology practice.	Mean age 13.9 (S.D. 2.2)	ABCDEFGHI
			Target range 11-18	
Wilson and Beskine [65] UK	Survey	73 parents via a survey on the UK CWD website	<5 (11%), 5–11 (55%), >12(34%)	ABCDEH
Amillategui *et al.*[66] Spain	Survey	499 parents from diabetes clinics	Target range 3-18	ABCDEFGHI
			3-6(12%), 7-10(26%), 11-14(38%), 15-18(24%)	
Pinelli *et al.*[67] Italy	Survey	220 parents from 15 diabetes units	Mean 10	ABCDEFI
			Target range 8-13	
Hellems and Clarke [68] USA	Survey	185 parents from diabetes clinics	Target range 5-18	ABCDEGHI
Jacquez *et al.*[69] US	Survey	309 parents from diabetes clinics	Mean 11.83 (S.D. 3.70)	ABCDEFGI
			Target range 4-19	
Lewis *et al.*[70] US	Survey	47 parents from diabetes clinics	ns	ABCEI
Yu *et al.*[71] US	Survey	66 parents from paediatric endocrinology unit	Mean 12.7 (*diagnosed at ≤5 yrs)*	ABCDEFGI
			Mean 12.6 (*diagnosed after 5 yrs)*	
Lin *et al.*[72] Taiwan	Interviews	12 mothers from diabetes clinics	Mean 8.4	ABCDEFGHI
			Range 7.3 to 9.2	
Ramchandani *et al.*[73] US	Survey	51 students (42 valid) from 5 hospital diabetes centres	Mean 20.1 (S.D. 1.6)	ABCDEFHI
			Range 18.4- 25.7	
Balfe [74], [75] Balfe and Jackson [76]	Interviews	17 students from 5 university health centres	Actual range 18-25	ABCDEFGHI
Balfe [77], [78] UK	Research diaries			
Wdowik *et al.*[79] US	Survey	98 students from 22 college health providers	Mean 24.4 (S.D. 7.4)	ABCDEFGHI
Wdowik [80] US	Focus group^a^	^a^10 students from 1 university health centre	^1^Target range 18–35 (2 over 24 years)	ABCDEFHI
	Interviews^b^	^b^15 students attended pre-college workshop at local diabetes centre representing 9 colleges across 7 states	^b^Target range 19-22	
Geddes *et al.*[81] UK	Case notes	55 students Referrals over a 10 year period to one hospital diabetes centre	Target range 18-24	ABCDEFGH
Ravert [82] US	Survey	450 students T1D on graduate surveys	Mean 20.3 (S.D. 1.6)	ABCDEFI
			Target range 18-25	
Wilson [83] UK	Interviews	23 students no details provided	Actual range 17-19	ABCEFGHI
			17 (30%), 18 (44%), 19 (26%)	
Miller-Hagan and Janas [84] US	Interviews	15 students Advertisements placed in one university	Mean 22.4	ABCDEFI
			Actual range 18-40	
Eaton *et al.*[85] UK	Interview	22 students	Mean 20	ABC
		From one university medical practice	Target range 19-21	
Amillategui *et al.*[62] Spain	Survey	111 teachers of children with T1D attending the paediatric units of nine public hospitals.	Experience of teaching a child with T1D (100%)	ABCDEFHI
**School personnel working with children and young people with T1D**				
Greenhalgh [86] UK	Survey	85 teachers of children with T1D who attended a diabetes clinic a local hospital 30 teachers	Experience of teaching a child with T1D (96%)	ABCDE
Bowen [87] UK	Survey	School nurse assigned to 5 schools	Had taught a child with diabetes (20%)	ABCDEFGHI
			Not linked into specific children with T1D	
Alnasir and Skerman [88] Latif Almasir [89] Bahrain	Survey	1140 teachers from 49 randomly selected schools	Not linked into specific children with T1D	ABCDEF [88]
				ABCDEFI [89]
Gormanous *et al.*[90] US	Survey	463 teachers from schools in one US state	Not linked into specific children with T1D	ABCDEHI
Tahirovic [91] Bosnia and Herzegovina	Survey	83 physical education teachers. All schools within the region included	Not linked into specific children with T1D	ABCDEFH
MacArthur [53] UK	Survey	11 teachers	Experience of teaching a child with T1D (100%)	ABCHI
		Linked with children from one local diabetes centre who took who took pre lunch injections at school		
Boden *et al.*[92] UK	Interviews	22 teachers	No experience (9%)/Currently teaching (46%) In directly involved (9%)/taught in previous year (27%) Taught a child though no longer in school (9%)	ABCDEFGHI
		25 primary schools with a child with diabetes in the school (currently or who had left very recently)		
Nabors *et al.*[93] US	Survey	247 teachers from 5 elementary schools in one city	Not linked into specific children with T1D	ABCEFGHI
Lewis *et al.*[70] US	Survey	65 teachers	Not linked into specific children with T1D	ABCEI
		222 schools in 3 counties were randomly selected to participate in the study.		
Rickabaugh and Salterelli [94] US	Survey	32 physical education teachers linked with 25 children with T1D from schools across three states.	Had taught on average 4 children with T1D	ABCDEGHI
Chmiel-Perzynska *et al.*[95] Poland	Survey	52 teachers Part of a wider survey	Currently teaching or had taught a child with diabetes.	ABCDE
			Not linked into specific children with T1D	
Fisher [96] US	Survey	70 school nurses from a convenience sample of 115 schools	Experience of children with T1D: 63%	ABCDEGHI
			Number of children with T1D: 0 (37%)/1 (31%)/2(21%)/3 (6%)/4(3%)/5(1%)	
Guttu *et al.*[97] US	Survey	21 counties, 19 provided school nurse services	Each county was characterised as having a good nurse-student ratio (1 nurse < 1,000 students) or a fair to poor nurse-student ratio (1 nurse >1,000 students	ABCDEI
Joshi *et al.*[98] US	Survey	43 school nurses from schools in 1 US state	Not provided	ABCEH
Nabors *et al.*[99] US	Survey	38 school nurses from schools in 3 US states	Experience of children with T1D: 87% Number of children with T1D: ns	ABCDEHI
Wagner and James [100] US	Survey	132 school counsellors attendees at two school counsellor association annual meetings	Experience of children with T1D: 83% children with diabetes in their schools.	ABCDEFGHI
			14% did not know if there were children with diabetes in their schools.	
			Number of children with diabetes average of 4 students	
Schwartz *et al.*[55] US	Survey	28 school personnel Linked with children from a hospital diabetes centre. 20 schools represented	Experience of children with T1D: 63% Number of children with diabetes:	ABCEH
		School nurses (85%);	0(5.9%) / 1–2 (27.5%)	
		Dieticians, teachers, & other (15%)	3–4 (41.2%) / 5–10 (13.7%)	
			>10 (11.8%)	
Darby [101] US	Interviews	11 school nurses helped students with CSII therapy	Experience of children with T1D: 100%	ABCDEFHI
		Survey of local schools across 3 counties	Number of children with T1D: 1-4	
		RN(n = 6), CNP or APN: (n = 2)/LPN (n = 3)		

**Key:** APN - Advanced practice nurses; CNP - Certified nurse practitioners; DAFNE - Dose Adjustment For Normal Eating; G1 – group 1, G2- group 2, LPN - Licensed practical nurses, NS – not stated, RR – response rate, RN – Registered Nurse.

**Quality criteria key:** A-Clear statement of the aims of the study; B-Adequate description of the context for the study; C-Clear specification of research design and its appropriateness for the research aims; D-Reporting of clear details of the sample and method of recruitment/sampling; E-Clear description of data collection; F-Clear description data analysis provided G-Attempts made to establish rigour of data analysis; H-Discussion of ethical issues / approval details; I-Inclusion of sufficient original data to support interpretations and conclusions.

**Figure 3:**
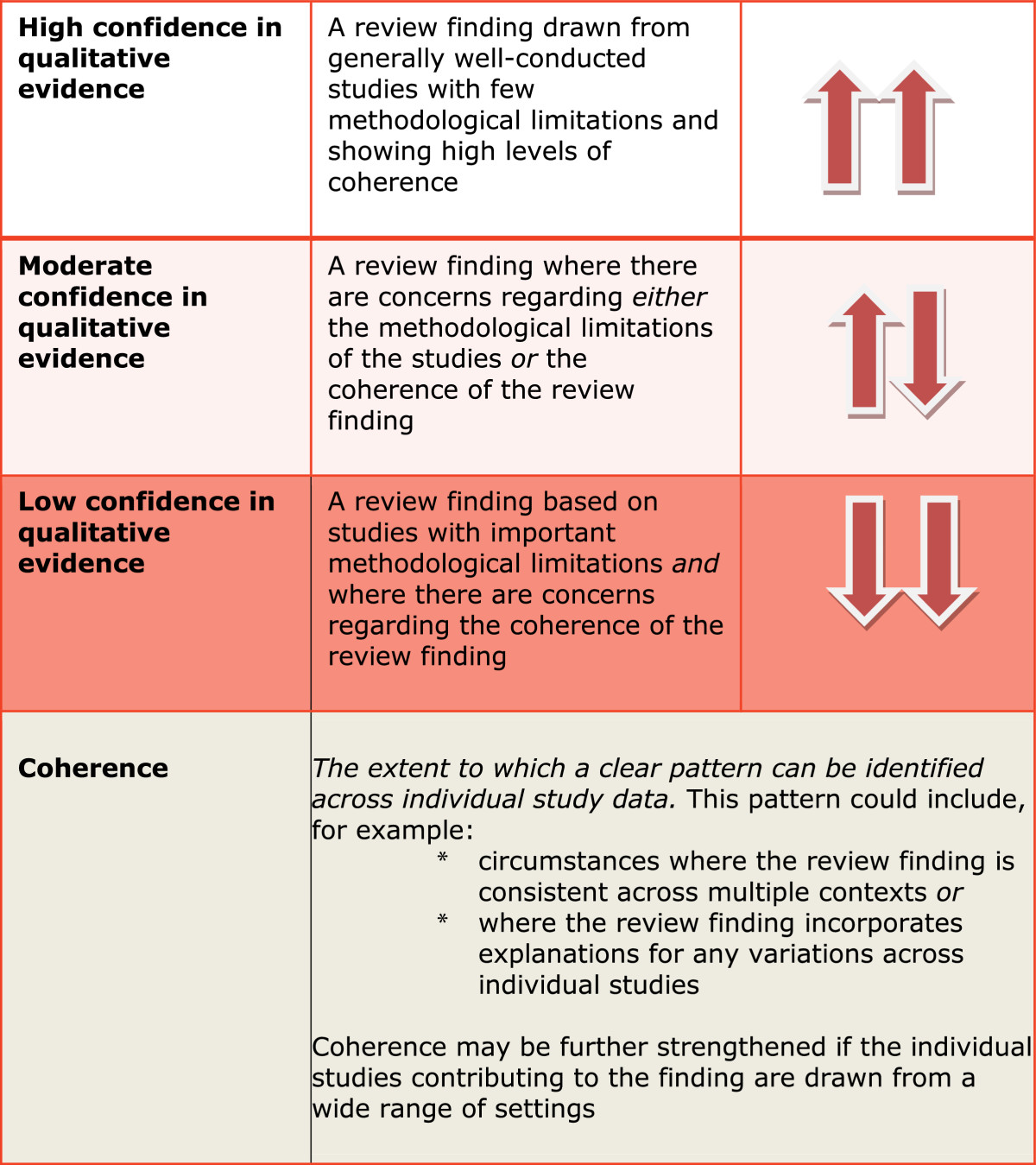
**CerQual: applying High, Moderate and Low confidence to evidence based on Glenton** ***et al.***[45]**.**

Confidence in synthesized qualitative and survey findings was assessed using the Confidence in the Evidence from Reviews of Qualitative Research (CerQual) tool developed by Glenton *et al.*[102], which uses a similar approach to GRADE. The original CerQual approach was designed for qualitative findings and we used the same process but included findings from surveys in the assessment of confidence. Confidence in findings is described as high, moderate or low (See Figure 3). All studies were included unless fatally flawed and study quality is reported for each stream.

### Data extraction

Additional study characteristics (Additional files 2 and 3) and results (Additional files 4 and 5) were extracted directly into pre-formatted tables and followed the format recommended by the Centre for Reviews and Dissemination (CRD) [34]. One researcher extracted the data and a second researcher independently checked extracted data for accuracy and completeness [34]. Any disagreements were noted and resolved by consensus among the researchers.

### Data synthesis

Three types of syntheses were performed. Firstly for stream 1 (intervention studies) meta-analysis was inappropriate due to the heterogeneous nature of the studies in relation to populations, interventions and outcomes. Instead the results from the studies (any summary measure) were reported in a narrative summary within and across studies. Secondly, for stream 2 we used Ritchie and Spencer’s thematic framework synthesis [103] for non-intervention studies. All included studies in stream 2 were then uploaded into the software Atlas Ti and an a priori index coding framework based on the conceptual framework presented in Table 1 and issues of interest mapped against review questions and objectives was applied to studies. Thirdly, a final overarching synthesis of intervention and non intervention studies was conducted. For this final synthesis a matrix was constructed that mapped best practice guidance against the age-related barriers and facilitators identified by children and young people, parents, school personnel and school health professionals and age-related interventions and outcomes in stream 1 (Additional file 6). We were particularly interested to see the extent to which interventions were effective and addressed the barriers identified by children, parents and teachers/health professionals, and built upon the facilitators to providing optimal care and management of children and young people with T1D in educational settings. We also identified gaps in evidence, assessed the robustness of the synthesis by making observations about the quality of included evidence, and looked specifically at the age and context of child participants in interventions compared with child participants in studies of attitudes and experiences.

## Results

### Description of the studies

Sixty-six studies were included in the review. For stream 1 (intervention studies) 11 were included (see Table 4, and detailed tabular summary Additional file 2). Only 3 out of the 11 studies were randomised controlled trials (RCTs) [30–32]; 1 was a controlled trial [80], 3 were before and after studies [35, 36, 38], 2 were analog experiments [39, 40], 1 cohort study [41] and 1 programme evaluation [42].

Of the 11 interventions, only 2 were explicitly reported as theory based [36, 37]. The study by Wdowik [79] utilised the Theory of Reasoned Action, and Social Learning Theory and developed an expanded Health Belief Model. The conceptual frameworks for the pilot study by Faro [36] were based on social learning and developmental theory.

Sample sizes were small and ranged from 20 to 156 with the exception of the cohort study where the number of nurses attending the continuing education programme was 417 [41]. Follow-up periods ranged from 3 months to 1 year. The majority of studies (9) were conducted in the US and 1 study was conducted in Canada [32].

For stream 2, 55 studies were included (see Table 5, and detailed tabular summary Additional file 3). Thirty-four studies used a survey design, 17 used a qualitative approach, 2 employed a mixed-method design, 1 utilized a survey followed by qualitative group interviews and 1 employed a retrospective survey using case notes.

### Stream 1: Effectiveness of interventions to support children’s and young people optimal T1D management in educational settings

Studies investigated different types of interventions and used different outcomes to assess their effectiveness and were too diverse to undertake a meta-analysis. A narrative and tabular summaries (see Additional files 2 and 4) are reported. The narrative summary and tables are organized into two groups: interventions focusing on children and young people with T1D at educational settings and interventions focusing on school personnel.

### Interventions focusing on children and young people with T1D at educational settings

Diabetes quality-of-life was measured in two studies [30, 80] Both studies found significant improvements on the treatment barriers subscale at 12 months (Izquierdo *et al.*[31], p = 0.039) and Engelke, [35] p = 0.01). Izquierdo *et al.*[31] also found a significant improvement (at 6 months: p = 0.017 which was maintained at 12 months).

Three studies [30, 31, 36] measured HbA1c levels (measure of glycaemic control). Two studies [30, 31] showed significant improvements in the HbA1c readings over a 3 month [30] (p < 0.05) and 6 month [31], (p < 0.02) period following the intervention, whereas the other [36] showed no significant change. The effect of the intervention on health service use was measured in two studies. This section of the analysis by Izquierdo *et al.*[31] was poorly reported but showed that urgent visits to the school nurse for diabetes related problems and urgent calls to the diabetes centre decreased significantly (p value not reported) and that there were significantly fewer hospitalisations (p value not reported), and emergency department visits (p value not reported). Whereas Faro *et al.*[36] did not show any significant differences for the frequency of hospitalization or emergency department visits.

One study investigated the diabetes knowledge of university students and reported that knowledge was significantly improved (p < 0.001) as a direct result of the intervention and was maintained at 3 month follow up (p < 0.001). They also showed a significant increase in the number of university students who knew their recent HbA1c results (p = 0.003) post intervention [37].

### Interventions focusing on school personnel working with children and young people with T1D

Six studies involved school personnel and the samples included both school nurses and school teachers [38], elementary school teachers [32], regular and special education elementary teachers [39], continuing education and pre service teachers [40] and school nurses [41].

The T1D knowledge of school teachers showed a significant improvement (p < 0.004) after the implementation of an education programme [38] whereas there was no significant change after the introduction of a compact disc (CD Rom) teaching tool [32]. This difference in findings could be attributed to the fact that all school personnel already had experience of caring for a child and young person with T1D and therefore already possessed a good level of knowledge of T1D [32] whereas only 38% of teachers in the study by Siminerio and Koerbel [38] had experience of children with T1D.

With regard to confidence, providing education through a CD Rom was found to significantly increase school teachers levels of confidence in managing diabetes (p < 0.016) [32]. Two further studies assessed teachers confidence in attributing class learning and behaviour problems to hypothetical students with T1D and found that a teacher’s level of perceived confidence to manage a child with T1D in their classroom was not related to the amount of disease related information they received [39]. However the more knowledge teachers were given about the consequences in the classroom of chronic health conditions the more confident they were in attributing chronic conditions to behavior (p = 0.007) [104].

Two further studies reported that perceived levels of competence for school nurses and suggested that where diabetes education was part of continuing education programs that school nurses ability to manage students with diabetes would be enhanced [41] and one study showed significant significantly improved results (p = 0.0001).

### Stream 2: Attitudes and experiences of children, young people parents and professionals

Best practice guidance (see Table 1) sets out optimal ways for children and young people to self-manage their T1D whilst at school. However, there is no specific guidance on the management of T1D specifically for college/university students but there are recommendations concerning all young people with T1D and alcohol within the NICE guidelines [24].

### Assembling school health care pans

Less than half (31-46%) of students had a written care plan [55, 65, 69]. School policies generally applied to the entire student body within a particular school and did not often consider the child and young person with T1D and their needs to perform T1D self-management at school [52, 60]. School nurses felt that a care plan for emergencies was important for facilitating the care of a student with T1D in the school environment [99].

### Checking blood glucose during the school day

Younger students (≤10 years) kindergarten/nursery through to junior/middle school) reported that they needed assistance with blood glucose monitoring during school hours [47, 68]. This was usually the role of school nurse [68]; or a designated member of the school staff [46, 47, 62, 65], peers [62] and in some instances parents [65, 68]. Older students(≥12 years) generally required less assistance once they were attending high/secondary school [65, 68].

### Accessibility and storage of supplies and snacks

Glucagon was found to be available at school for between 34–49% of students [47, 66, 69]. A high percentage of both children (60%) [47] and parents (64 percent) [66], felt that that glucagon should be readily available, together with a person who was aware of how to administer it. Only 10% of children had experienced a serious hypoglycaemic episode at school [66]. In only a very small number of cases was a call to emergency services made (3% [67]), or glucagon administered [68].

Students considered having accessible test kits and snacks available whenever they needed them as important [46]. Healthcare professionals felt that schools should rethink policies which prevented children having easy access to their medical equipment [92]. Students reported that storage of medication or items related to T1D was in a variety of places i.e. with the student [52–54], another room in school, in classroom, in the school office [52], the nurse’s office [54], secretary/teachers office/desk [54]. School nurses felt that they could better support students if they could have ready “access” to snacks and testing kits as well as appropriate medical supplies [98, 99]. Most students with T1D took a snack to school [50] but some reported that they were not allowed to eat snacks when they needed to [54, 60]. However, some students reported that they it difficult it have a snack before physical education (PE) lessons [58].

### Administering insulin during the school day

When insulin was administered in school, between 46–97% [47, 51, 52, 63, 68] self-injected, especially older students (11 years and older) who attended secondary/high school [65, 68]. For a small minority of students it was the school nurse (18%) [54] or a member of school staff (1-6%) [47, 54, 65, 67]. For younger students (6–10 years [47], ≤ 12 years [65],:≤10 years [68]) (2-32%) it was the young student’s parent who came into school to give an injection or administer a bolus if the child needed insulin if no one at school was trained and/or allowed to administer insulin [47, 65, 67, 68]. In certain instances, however, students occasionally had to go home if nobody was available to administer insulin [65] and when this was not possible a small minority reported treatment modifications were made because of a lack of cooperation from the school [47, 66]. A small number of students were also not allowed to inject insulin whilst in school [50].

Some students who self-injected were supervised (20–49%) whilst taking their insulin [54, 65]. This was usually the role of a school nurse or a designated member of the school staff [68]. Younger children (6–10 years) required more support [47] especially if they were in kindergarten/nursery (5–6 years) or infant/elementary school (7–10 years) [68]. Students generally appreciated being reminded by the teaching staff to administer their insulin [54].

Only 30–54% of students were permitted to check their own blood glucose in the classroom [65, 68, 69] increasing to 74% for students at high school [68]. For those not allowed to perform blood glucose monitoring in the classroom, a number alternative locations are provided which include medical room [65], school office [65], head teachers office [65], anywhere [65]. Students have been shown to demonstrate significantly better glycaemic control when they are given flexibility to decide where to perform self-care behaviours [61].

With regard to insulin administration, students reported problems with a lack of a private location within the school where they could administer injections [50, 52, 69]. Locations provided or chosen by the students included first aid/medical room/health office [50, 52, 60, 65], toilets/cloakroom [50, 52, 65], classroom [50, 69], “Wherever I have my lunch”, school dining room [53], cupboard in the school office [53], school office [65], locker between classes [61] and head teachers office [65]. However some students reported being happy with their usual place [53]. Most students with T1D took a snack to school [50] but some report that they were not allowed to eat snacks when they needed to [52, 60].

### Accessibility of and participation in physical education in schools

Whilst some schools provided strategies so that students with T1D could participate in sport [52, 66], some parents stated that their assistance and presence was required during and/or after school sport [67] especially for younger children (age not specified) [46] and in some instances their children were not allowed to play sports such as football [70]. Children felt that participation in such activities could be facilitated if a nurse was still on site [49] as T1D supplies were often locked in the nurse’s office [58]. Older children said that their coaches needed to be more knowledgeable about T1D [46].

### Food and dietary management

The timing of school lunches was a commonly reported problem [52] with only 25% of primary school teachers and 38% of secondary school teachers appreciated that students with T1D should not be late for a meal [86].

Some students, parents and school nurses however felt that food choices provided in the school canteen, vending machine and classrooms, which were conducive to healthy T1D management, were limited [60, 98] and that ensuring snacks and appropriate foods were available can reduce barriers to good control at school [99]. Students reported that they would benefit from more healthy food and drink options [57, 66, 70], prominent and consistent information about prepared food and developing nutritional analyses for all foods available in the cafeteria as a way of helping in choosing meal options in the cafeteria [60]. As a solution, some students in some instances had to take their own lunch to school [55]. Some parents reported that schools were not able, or did not consider it their responsibility, to modify diets to enable children with T1D to eat a school lunch [66].

Parents sometimes reported having trouble getting nutritional information about foods served and portion sizes from their child’s school. This made it difficult to plan ahead whether using a constant carbohydrate approach or insulin to carbohydrate ratio [23]. Only 7% of students reported that their school cafeteria made carbohydrate content of prepared foods available [61].

### Planning for special events, field trips, and extracurricular activities

Parents reported that their child’s T1D affected their decisions regarding extracurricular activities [61]. In some instances parents/guardians were asked to act as chaperone on field trips, especially for younger children [68], but parental attendance should not be considered a prerequisite for participation by students with T1D [19].

Even though teachers felt that all children with T1D should be allowed to go on extended trips with the school [86], between 15 and 20% [47, 62, 65, 66] of parents reported experiencing difficulties with their school over responsibility of the children during 1-day trips especially for children under 10 years of age [47, 66] with greater problems for trips which extend over several days [66]. In some instances, parents reported that their child was not allowed to participate in outside school trips unless accompanied by a parent or a school nurse [70] and for others, the schools had specific policies in relation to medication on school trips and worked with the family to ensure that children and young people could participate [52]. School nurses reported that planning for management during ‘out of town’ trips was critical [99].

### Flexible accommodation for exams and tests

Flexible accommodation with exams and educational tests was considered important as older children (>11 years) reported (23-39%) that if they experienced hypoglycaemic events before or during an exam, they did not have the opportunity to do the exam again [47, 62].

### Dealing with emotional and social issues

Students with T1D often expressed a feeling of ‘being different’ from their peers due to their T1D [50–52, 55, 92, 99]. They also felt embarrassed if they ‘had a hypo’ [51, 55] or when they had to check their blood glucose or take medication at school [55, 59]. These feelings could act as barriers to positive T1D self-care behaviours in school [98]. In an attempt to not appear different from their peers, young people compromised their T1D self-management by choosing not to alleviate their symptoms [51]. Some students reported that they did not like peers watching them inject [59]. On the other hand, some students stated that they did not mind others seeing them take their insulin [53].

Some parents reported that their child was bullied/picked on at school because of their T1D (26%) [65] and a small percentage of students themselves reported problems with their peers such as diabetes-related bullying or teasing [50, 51, 61]. This was more of a problem for older students (over 12 years) in high/secondary school [65]. As a result, students balanced the need to have peers around them who knew about their T1D and the emergency management strategies by telling one or two close friends they felt they could trust [52] to keep the T1D a secret from most of their peers [46, 51]. Some talked about such peers as a T1D “buddy”, who with training would be able to recognize hypoglycaemia, alert staff, prompt self-care, buffer teasing, and escort the student to the nurse [61]. When age and level of HbA1c was taken into consideration, students who received help from trained peers were found to have significantly higher quality-of-life in the school environment [61].

### Assisting the student with performing diabetes care tasks

School healthcare personnel were more able to facilitate optimal management when confident in providing *support to children, able to communicate with healthcare providers*, *possessed T1D knowledge and skills and undertook regular education and training.* The main difference between the UK and US policy was the provision of a school nurse. There are no specific recommendations regarding the role of the school nurse within UK guidelines. The US guidelines recognise the school nurse as the most appropriate person in the school setting to provide care for the student with diabetes.

Despite the policy intent, many US schools did not have a full-time nurse, and sometimes a single nurse covered more than one school [105–107]. The numbers of students reporting that their schools had a school nurse on site varied widely (Spain: 21 to 48% [47, 62] and US: 65 to 95% [54, 68, 69] with a smaller percentage working full time [46, 68], and some students reported that they received support from school counsellors (57%) for non-medical, T1D related problems [61]. US children reported that even though they had school nurses assigned to their schools that these nurses did not come every day, and they worried about what might happen if they “got very low” and no one was there to help them [46]. Those nurses who did come every day were not always on site all day and this caused problems for some children as supplies were often locked in their office [46]. Just under 50% of children from Spanish schools [47, 66] felt that a nurse should be available daily during school hours to help with the management of T1D. Parents also expressed concern about the qualifications and training and preferred the presence of a daily onsite nurse [70] as opposed to a health aide who they felt was unable to provide adequate care [60]. Over 80% of school nurses felt that numbers of school nurses that were available across schools for students with T1D were inadequate and that a school nurse should be available on school premises during the school day if a student with T1D was enrolled [55]. Guttu *et al.*[97] demonstrated that a significant correlation existed between increased presence of school nurses and care and services provided to children with T1D.

The greatest support students received at school came from teachers [46, 47, 62, 66]. A high proportion of school personnel however (65%), expressed concern about the potential liability and child safeguarding issues when caring for students with T1D in school [55], which was related to concern surrounding exposure to and interaction with children’s bodies, especially someone else’s child [92].

The most supportive teachers were those who (1) were flexible in allowing children and young people to test their blood glucose [46], (2) allowed them go to the nurses’ office in the middle of a class or test [46], (3) included snack times for the entire class based on the schedule of students with T1D [60], (4) kept a supply of juice or snacks for students with T1D to use during an emergency [60]. Some students (14–45%) reported that they were not allowed to snack in class when they needed to [50, 61, 69] or that teachers delayed the student attending the nurse’s office to treat their hypoglycaemia [61].

### Diabetes education and training of school nurses and school personnel

Some young people felt that school nurses were well educated about T1D [60], whereas others felt the nurses’ knowledge could be improved [60]. A third of school nurses perceived their own levels of T1D knowledge as being low to average [98] and rated themselves as moderately confident in providing T1D care and education [96]. Self-efficacy was significantly higher if they were currently participating in the care of children with T1D, when there were students with T1D in the school system, and when they were supervising students with blood glucose meter testing [96]. Only 20% felt adequately prepared to assist a child with hypoglycaemia [55].

Parents reported concerns about Continuous Subcutaneous Insulin Infusion (CSII) therapy in school, specifically testing, bolusing, and pump management [64]. As well as being completely unfamiliar with pumps or CSII therapy [64], the biggest challenge faced by school nurses was learning to count carbohydrates when a student was on CSII therapy [101]. When they first encountered a student on CSII therapy, school nurses were scared, intimidated and overwhelmed as a consequence of their lack of education and experience with this new technology [101].

The majority of school nurses (94%) had up-to-date T1D reference materials in their offices [96] with some nurses obtaining information about T1D from the internet and professional books and magazines [98]. Just over a third (36% of school nurses reported that they had attended a conference on T1D during the past year [96]. Barriers to acquiring new information by school nurses were time constraints (37%), lack of access to education/regular updates and inadequate training (28%) [98].

Children and young people reported that they would like teachers to be better informed about T1D and to have better knowledge about T1D in order to help the students manage their T1D in school [46, 60, 62]. The lack of education received by school personnel was described as being problematic by parents [60]. Most teachers had received written information about children’s T1D (82%) [65], whereas only 22% of both regular education and special education teachers indicated that they felt well informed regarding T1D [93]. Students and parents felt that teachers should receive written instructions in order to improve the management of T1D and to improve the child’s integration at school [47, 66], in particular information about the symptoms and steps to be followed in case of a hypoglycaemia, more information about T1D in general, information regarding the optimal management of emergencies [62] that should be kept in their class and in the common areas [47]. School nurses however felt that school staff needed to improve their T1D knowledge and this then could reduce barriers to good control at school [99]. Both children and parents felt teachers had a basic knowledge about T1D and that they were adequately trained to care for children to manage T1D [55, 62, 66] although sometimes they reported concerns that there was confusion at school between Type 1 and Type 2 diabetes [65].

### Recognizing and treating hypoglycemia and hyperglycemia

Diabetes healthcare professionals’ biggest concern was about the ability of teachers to spot the onset of hypoglycaemia and react quickly [92]. Teachers were confident in their ability to be able to recognise the signs of hypoglycaemia (70-71%) [87, 95] but were less confident that they would be to cope with emergencies that arose from high or low blood sugar levels (42-63%) [87, 95].

### Communication between school health personnel and diabetes healthcare providers

Having regular appointments with healthcare providers and written communication between the healthcare providers and school nurses regarding management needs for school and increased information exchange between the two was seen as beneficial [92, 99]. However, communication between the healthcare team and the school nurse was reported to only occur often or very often for a quarter of nurses [55]. Healthcare providers were often difficult to reach and were too busy to respond to questions [98].

### Self-care and management at college/university

Important facilitators to optimal self-care and management included the ability to balance T1D and student lifestyle and to have specific diabetes management strategies in place when drinking alcohol.

College/University students found it challenging trying to juggle all aspects of T1D. Students said that lack of a perceived routine at university was a barrier to effective self-management [80] with many reporting little or no time to engage in practices such as blood glucose testing [76, 79, 80, 83, 85], exercising [79, 80, 85], eating snacks during the day [79, 83] and injecting in a suitable environment [83]. Inadequate finance was also cited as a barrier to successful T1D management with students studying in the US as they worried about the cost of blood glucose monitoring strips [80]. Whereas in the UK this is not an issue and students reported that being on a student budget did not affect their control because they get free prescriptions and supplies, they either lived in halls of residence where meals were provided, or they borrowed money from their parents, or built up a student debt to be paid off later [85]. Having to eat in class or carry food or supplies around and having to test blood glucose were seen as barriers to diabetes management [80]. Motivating factors were being physically able to keep up with their peers and the of long term consequences [80]. When significant barriers or negative emotions were present Wdowik *et al*. [79] reported that students with positive attitudes and good intentions however, may be unable to engage in desired self-care behaviours when complications arose. Whilst some students wanted their friends to know about their diabetes and to be able to help them in an emergency others were concerned about what their peers would think if they knew they had diabetes and wanted to avoid being treated differently.

The majority of students reported that they drank alcohol whilst at university [74, 82, 85]. Drinking excessive amounts of alcohol can interfere with metabolic control and can induce hypoglycaemia and in the longer term it can worsen or increase the risk of diabetes complications [108].

Students reported using several strategies when drinking alcohol which were in keeping with current guidelines for young people [24] and general advice for students [109]. These included the following: eating before and/or during drinking [74, 80, 82, 84]; keeping track of how many drinks you were having [82]; determining, in advance, not to exceed a set number of drinks [80, 82, 84]; avoiding drinking games [82]; having a friend let you know when you have had enough [82]; alternating non-alcoholic with alcoholic beverages [82]; pacing drinks to 1 or fewer per hour [82]; choosing not to drink alcohol [84]; drinking an alcohol look-alike (non-alcoholic beer, punch etc.) [82]; avoiding or diffusing peer pressure [84]; limiting the frequency of going out to parties and bars [84]; drinking with trusted friends [80, 84] who understood the symptoms of low blood sugar that would mimic intoxication [80], and checking or monitoring blood glucose levels [76, 84]. Students reported that they usually tested their blood glucose at home, or in more private spaces, before and after going out, rather than while they were out [76].

Students felt that they did not receive adequate support from their college or university that would enable them to balance the demands of further education and management of their T1D. An example was being allowed to manage their glycemic control during examinations [83].

Although some students preferred to continue to receive care from their diabetes team in their home town [85] others felt unsupported by their T1D team [83]. For example they reported that care had to stop with their old diabetes team now that they were at university and they experienced difficulties assessing a new local diabetes team [83], whilst others felt that they had not received enough information particularly in relation to managing their diabetes and drinking alcohol [84].

### Overarching synthesis mapping guidance imperatives against barriers and facilitators and interventions for optimal self-management in educational settings

Additional file 6 shows the juxtaposition of guidance imperatives mapped against barriers and facilitators to optimal T1D management in educational settings identified by children, parents and professionals, mapped against interventions to promote optimal T1D management in educational settings.

Overall, interventions aimed to provide additional targeted help and support for children to self-manage some specific aspects of their T1D in schools, or interventions to increase knowledge and awareness of school nurses and staff, and better communication with diabetes professionals. These foci broadly match with children’s and parents views and experiences of what needs to be done to optimize children’s T1D self-management in education settings, which in turn map onto interventions outlined in guidance as shown in Table 1.

In the following section, where reciprocal guidance, quantitative and qualitative evidence exists addressing the same issues, a synthetic line of argument derived from integrating intervention effectiveness and views evidence is presented along with an assessment of the certainty/confidence in evidence.

#### Assembling school health care plans

Having a diabetes medical management plan (DMMP) was shown to be effective in improving diabetes-specific quality of life with regard to treatment barriers (for example: embarrassment about having diabetes, arguments about patient care, and difficulty complying with their diabetes plan) (GRADE:Low [35]).

School nurses considered plans to be important for optimal diabetes self-management (CerQual:Low). Both children and parents agreed that it was difficult to manage their diabetes at school when DMMPs were not in place, but parents confirmed that this was often the case, especially with regard to providing suitable locations for blood glucose monitoring and insulin administration, allowing students to eat snacks when needed), timing of school lunches, participation in physical activity programmes and extra curricular activities. Children, parents, school personnel and school counsellors all agreed that students who had a DMMP that covered these areas were facilitated to optimally manage their diabetes whilst at school (CerQual:Moderate).

#### Checking blood glucose and administering insulin during the school day

Providing support from an adult, specifically a school nurse, was effective in promoting optimal blood glucose and insulin management for children age 10 to 17 (GRADE:Very Low [30]). Children (especially younger children under 13 years) and parents confirmed that they needed this type of support and those who received it said that they benefitted from it, whilst those that did not reported ongoing difficulties with their diabetes self-management at school (CerQual:Moderate). However just having someone to review blood glucose readings was not effective (GRADE:Very Low [36]).

#### Food and dietary management

When students were provided with school menus that included carbohydrate servings for all food items listed this did not have a significant effective on HbA1c levels (GRADE:Low [36]). However, children and parents reported that it was difficult to manage their diabetes when the canteen did not offer healthy choices, or sufficient information (CerQual:Low). Whereas when snacks and appropriate food and drinks were available (CerQual:Moderate) and nutritional were information were provided (CerQual:Low) this was seen as facilitating optimal T1D management.

#### Communication between school health personnel and diabetes healthcare providers

Healthcare professionals considered that having regular appointments with healthcare providers and written communication between the health care providers and the school nurse regarding management needs for school and increased information exchange between the two was seen as something that would be beneficial (CerQual:Moderate). Strengthening general collaboration between school health personnel and the children’s diabetes center staff to resolve diabetes-related school problems and enhance diabetes management showed no significant differences HbA1c. Although a trend towards increased blood glucose monitoring at home was observed and the frequency of insulin administrations at school doubled. Poor communication with health care providers and the school nurse is seen by parents and health care professionals to be a barrier to optimal T1D management (GRADE:Very Low [36].) Whereas exchanging graphical and tabular blood glucose measurement information between the school nurse with the diabetes center nurse via telemedicine was effective in significantly improving diabetes QOL (treatment barriers and treatment adherence) and HbA1c which was maintained at follow up (GRADE:Very Low [31]).

#### Diabetes education and training of school nurses and school personnel

Continuing education programmes were effective in increasing perceived competence of school nurses (GRADE:Low [42]) and enabled nurses to feel that their ability to manage students with diabetes was enhanced (GRADE:Very Low [41]). Parents and students reported that inadequate knowledge, was a barrier to optimal diabetes management in school and nurses themselves reported that they felt inadequately prepared to assist students with hypoglycemia and manage those on CSII therapy. When nurses had access to up to date information and were able to update their knowledge on skills on a regular basis, then they were more able to assist a students with T1D. Whereas not having any time or access to education and regular updates is seen as a barrier (CerQual:Low).

Education was shown to be effective in increasing confidence but not knowledge of school personnel(GRADE:Very Low [32]). Providing school personnel with basic disease information about a student with T1D is effective in increasing confidence and their ability to make accommodations for the student within the classroom (GRADE:Very Low [39, 40]). Care was found to be optimally facilitated when teachers had a basic knowledge about T1D and when they were adequately trained to care for children to manage T1D (CerQual:Moderate). A lack of diabetes knowledge by school personnel was seen by health care professionals, students and parents to be a barrier to the optimal management of T1D in schools (CerQual:Moderate), and healthcare professionals biggest concern was about the ability of teachers to spot the onset of hypoglycaemia and react quickly (CerQual:Low).

Students and parents felt school personnel would benefit from written information about T1D, but teachers themselves were not willing to participate in diabetes training. School personnel received the majority of information about diabetes from parents (CerQual:Low). Students, parents and school counselors reported that training in diabetes management for school staff was seen as beneficial , especially in how to deal with an emergency diabetes situation (CerQual:Moderate).

#### Self-care and management at college/university

When students were supported on campus, knowledge and attitudes and diabetes self-care practices were significantly improved (GRADE:Very Low [37]). Self-management was easier for students who are able to juggle all aspects of T1D with being a student, and have strategies in place for alcohol consumption, whereas those students who could not manage this struggled to engage in self-care practices, such as poor adherence to dietary recommendations (CerQual:Moderate), and not having adequate finances as a barrier (CerQual:Low).

## Discussion

Irrespective of setting or country context, many children and young people struggle with their T1D in educational settings due to a myriad of barriers to optimal self care and management that are needlessly put in their way. Some young people risk their health and wellbeing by disengaging from active self-care during school hours to avoid drawing attention to themselves or because unnecessary barriers prevent them optimally self-caring. School nurses and school personnel are commonly inadequately trained and many are not able or willing to support children and young people to manage their T1D on a daily basis.

Findings reinforce the appropriateness and importance of intentions contained in guidelines for schools to support students with T1D to optimize their self-care and management during school hours. Schools have a vital role to play in supporting children and yet many failed to fully implement basic common sense principles from mainly best-practice and some evidence-based guidelines. There appears to be no audit or feedback system to assess compliance with guidelines or to continuously improve practice and outcomes for children with T1D. The lack of translation of guidelines and monitoring of school culture and practice towards children with T1D urgently needs addressing.

Optimal management and control of T1D in children and young people in schools is critically important and if effective can reduce the incidence and delay the impact of associated microvascular and other long-term complications [4]. Better T1D management also has shorter-term benefits, including improved academic performance and school attendance, reduced hospital admissions and greater satisfaction with services [12].

The few child level and school level interventions that were effective provided additional targeted help and support for children to self-manage their insulin administration and blood glucose monitoring (especially younger children), and educational interventions to increase knowledge and awareness of school nurses and school personnel, and health system interventions such as telemedicine to facilitate better communication between schools and diabetes professionals. The review has limitations as none of these studies explored cost-effectiveness. Although these intervention studies had methodological concerns, their intentions matched with the views of children, parents and professionals as to what was needed to improve T1D management whilst children are at school. These interventions show potential for further development and refinement and more robust evaluation with large scale pragmatic cluster RCTs.

For the interventions that appear intuitively sensible – such as proving additional targeted and tailored support to children (especially younger children) for blood glucose monitoring and insulin management in school, there is a need to identify and train an appropriate cadre of people to do this as it is unlikely that it will be cost-effective or feasible to employ wrap around qualified and expensive school nurses. As some teachers appear resistant to taking on this role, there is a need to explore more novel and cost-effective solutions such as recruiting volunteer adults with T1D (i.e. following the established model of delivering additional school reading support), or lay health trainers [110] to support professionals and children to manage a range of health needs in schools including T1D. There are other models that could be worth exploring such as peer to peer support by teenagers and young people who are able to optimally manage their T1D, as well as peers without T1D.

Interventions such as food labeling for carbohydrate content of canteen food which were said by children and parents to be needed but were found to be ineffective require further research to find out why.

Interpretation of evidence is limited by the lack of novel complex intervention, implementation and evaluation research specifically focusing on supporting children and young people with T1D in educational settings. Most of the intervention studies were poorly reported, with many not including estimates of precision (such as confidence intervals) alongside p values. We identified no school-based interventions to promote positive coping or resilience and yet children, parents and professionals all said that many children and young people struggled to cope with their T1D at school and many had been affected by bullying. Nor did we locate any studies of children’s T1D information resources or diabetes management tools to specifically support children’s self-management whilst at school.

There is also a critical lack of research to inform development of interventions to support young people managing independently of their families whilst studying at further and higher education settings. From the two linked studies identified [37, 79] it was clear that interventions required by college and university students with T1D are distinctly different to school age students and need to encompass an integrated motivational, educational and lifestyle approach that can be individually-tailored and incorporate a high degree of peer support.

There is a wealth of qualitative and survey evidence describing the experiences of children, parents, and professionals. Children’s support needs, the circumstances in which children were well supported, and what needed to be done so that children were not left struggling to manage their T1D were clearly articulated and need to be addressed. There is however not an even spread of evidence across cultures and contexts. School peers are underrepresented and studies vary in quality. Confidence in the transferability of synthesized findings was assessed as ‘moderate’ because studies were limited to specific contexts or had some issues with methodological rigor. As with all international reviews of this type, readers are required to make judgments about the comparability of their local context and supplement with local evidence where appropriate.

Finally, we used the review findings along with additional primary qualitative research with children to subsequently inform development of a complex and general age-appropriate, individually-tailored, children’s T1D information and self-management intervention (self-care information packs and diabetes diaries to manage and titrate insulin doses) [111]. Although our complex intervention focused on T1D self-care management generally in any setting, and not specifically on school settings, we were interested to find out if the intervention was used at school by children. Our intervention was evaluated in an adequately powered pragmatic RCT that achieved 100% recruitment. Similar to other UK RCTs exploring [112–118] various general (rather than school specific) psycho-educational T1D interventions that were commissioned at the same time, our complex intervention was found to be no more effective than usual care, and was by itself not sufficient to help children navigate the barriers to optimal T1D management experienced by children at school. The lack of impact of any of the recently completed trials of general children’s T1D self-care interventions in the UK shows how difficult it is to change children’s and their family’s behaviour to favour optimal glycaemic control and that other barriers to optimal self-care and management, such as barriers at school (as one example) can negatively impact on outcomes generally. Our embedded process evaluation [111] involving interviews with 139 children/parents/healthcare professionals provides external validation for the barriers and facilitators to optimal T1D management in educational settings reported in this review.

## Conclusions

This novel mixed-method systematic review is the first to integrate intervention effectiveness with views of children, parents and professionals mapped against guidelines for the optimal management of children and young with T1D in education settings. The key messages are as follows. Self-care by children and young people in educational settings could be improved by fully implementing school T1D guidelines and auditing their impact in a quality improvement process. The evidence-base is limited by methodological quality and there are gaps in knowledge of what works. There are important gaps between what children, young people, parents say are barriers to optimal T1D self-management in educational settings and robustly evaluated interventions that seek to tackle these issues. Telemedicine between healthcare providers and schools, and individually-tailored support for school children is effective in specific contexts, but more robust evaluations are required. Comprehensive lifestyle and relationship management approaches for college and university students warrant further development and evaluation.

## Authors’ information

JN: Professor of Health and Social Services Research and Child Health at Bangor University and lead of the Cochrane Qualitative and Implementation Methods group.

DE: Research Officer Cardiff University.

LL: Florence Nightingale Foundation Chair of Clinical Nursing Research at Cardiff University and Paediatric Diabetes Specialist Nurse.

LS: Research Officer Bangor University.

JG: Consultant in Children’s Diabetes Endocrinology, Cardiff University School of Medicine.

## Electronic supplementary material

Additional file 1: Search strategies.(DOC 42 KB)

Additional file 2: Additional study characteristics of included intervention studies for children and young people with T1D at educational settings.(DOC 84 KB)

Additional file 3: Study characteristics for non-intervention studies (Stream 2).(DOC 158 KB)

Additional file 4: Summary of results (Interventions focusing on children and young people with T1D at educational settings).(DOC 54 KB)

Additional file 5: Study methods, quality appraisal and summary of results (Stream 2).(DOC 208 KB)

Additional file 6: Overarching synthesis matrix of entire dataset mapped against best practice.(DOC 80 KB)

Below are the links to the authors’ original submitted files for images.

Authors’ original file for figure 1

Authors’ original file for figure 2

Authors’ original file for figure 3

Authors’ original file for figure 4

## Abbreviations

CerQual: Confidence in the Evidence from Reviews of Qualitative research
CD Rom: Compact disc
CSII: Continuous Subcutaneous Insulin Infusion
DMMP: Diabetes medical management Plan
EPPI Centre: Evidence for policy and practice information centre
GRADE: Grading of recommendations assessment, development and evaluation
HbA1c: Measure of glycaemic control
ISPAD: International Society for Pediatric and Adolescent Diabetes
PDSN: Pediatric/Paediatric Diabetes Specialist Nurse
PE: Physical education
RCT: Randomised controlled trial
SPICE: Setting, Population/Perspective/People, Intervention/Issue of Interest, Comparison, Evaluation
T1D: Type 1 diabetes
UK: United Kingdom
US: United States.
