# Supplementary material for: An ongoing struggle: a mixed-method systematic review of interventions, barriers and facilitators to achieving optimal self-care by children and young people with Type 1 Diabetes in educational settings
Source: BMC Pediatr. 2014 Sep 12;14:228. doi: 10.1186/1471-2431-14-228 (PMC4263204; doi:10.1186/1471-2431-14-228)
Supplement: Supplementary file 1 — Additional file 1: Search strategies.(DOC 42 KB) [file 12887_2014_1206_MOESM1_ESM.doc]

PsycINFO Database

1. university.m_titl. (13278)
2. Schools/ (16489)
3. school$.m_titl. (82027)
4. institute.m_titl. (1757)
5. nurser$.ti,ab. (3759)
6. kindergarden.ti,ab. (22)
7. kindergarten.ti,ab. (10465)
8. polytechnic.ti,ab. (198)
9. college.ti. (29473)
10. (high adj1 school).ti,ab. (41051)
11. (junior adj1 high).ti,ab. (4859)
12. (pre adj1 school).ti,ab. (2015)
13. pre-school.ti,ab. (1977)
14. grade.ti,ab. (55400)
15. (educational adj1 establishments).ti,ab. (35)
16. (educational adj1 setting$).ti,ab. (2540)
17. (tertiary adj1 education).ti,ab. (232)
18. (further adj1 education).ti,ab. (743)
19. (higher adj1 education).ti,ab. (10222)
20. 1 or 2 or 3 or 4 or 5 or 6 or 7 or 8 or 9 or 10 or 11 or 12 or 13 or 14 or 15 or 16 or 17 or 18 or 19 (214647)
21. diabetes.ti,ab. (12090)
22. diabetic.ti,ab. (3175)
23. DM.ti,ab. (1140)
24. IDDM.ti,ab. (231)
25. T1DM.ti,ab. (48)
26. exp Diabetes/ (8011)
27. 21 or 22 or 23 or 24 or 25 or 26 (14303)
28. p*diatric.ti,ab. (13680)
29. child$.ti,ab. (445255)
30. adolescen$.ti,ab. (135127)
31. toddler$.ti,ab. (4745)
32. youth.ti,ab. (40168)
33. teen$.ti,ab. (12694)
34. youngster$.ti,ab. (2709)
35. juvenile.ti,ab. (15300)
36. pubert$.ti,ab. (4710)
37. (young adj1 adult).ti,ab. (6028)
38. (young adj1 person).ti,ab. (585)
39. (young adj1 men).ti,ab. (2866)
40. (young adj1 patients).ti,ab. (782)
41. (young adj1 women).ti,ab. (4003)
42. (young adj1 people).ti,ab. (12719)
43. (years adj2 age).ti,ab. (21208)
44. (years adj1 old).ti,ab. (10513)
45. student$.ti,ab. (311485)
46. exp Students/ (159324)
47. 28 or 29 or 30 or 31 or 32 or 33 or 34 or 35 or 36 or 37 or 38 or 39 or 40 or 41 or 42 or 43 or 44 or 45 or 46 (867923)
48. 20 and 27 and 47 (176)
49. limit 48 to (english language and yr="1996 -Current") (129)

Medline database

1. exp Universities/ (11972)
2. university.m_titl. (13947)
3. Schools/ (9353)
4. school$.m_titl. (26302)
5. exp Academies/ and Institutes/ (5107)
6. institute.m_titl. (5335)
7. exp Nurseries/ (428)
8. nurser$.ti,ab. (3045)
9. kindergarden.ti,ab. (6)
10. kindergarten.ti,ab. (1452)
11. polytechnic.ti,ab. (111)
12. college.ti. (8807)
13. (high adj1 school).ti,ab. (8789)
14. (junior adj1 high).ti,ab. (841)
15. (pre adj1 school).ti,ab. (1395)
16. pre-school.ti,ab. (1386)
17. grade.ti,ab. (112920)
18. (educational adj1 establishments).ti,ab. (90)
19. (educational adj1 setting$).ti,ab. (455)
20. (tertiary adj1 education).ti,ab. (207)
21. (further adj1 education).ti,ab. (737)
22. (higher adj1 education).ti,ab. (3312)
23. 1 or 2 or 3 or 4 or 6 or 8 or 9 or 10 or 11 or 12 or 13 or 14 or 15 or 16 or 17 or 18 or 19 or 20 or 21 or 22 (186008)
24. exp Child, Preschool/ or exp Child/ (590330)
25. exp Adolescent/ (657111)
26. p*diatric.ti,ab. (99491)
27. child$.ti,ab. (418384)
28. adolescen$.ti,ab. (85797)
29. toddler$.ti,ab. (3123)
30. youth.ti,ab. (17622)
31. teen$.ti,ab. (10697)
32. youngster$.ti,ab. (946)
33. juvenile.ti,ab. (23704)
34. pubert$.ti,ab. (12807)
35. (young adj1 adult).ti,ab. (9037)
36. (young adj1 person).ti,ab. (315)
37. (young adj1 men).ti,ab. (4897)
38. (young adj1 patients).ti,ab. (8286)
39. (young adj1 women).ti,ab. (8137)
40. (young adj1 people).ti,ab. (8241)
41. (years adj2 age).ti,ab. (119312)
42. (years adj1 old).ti,ab. (48860)
43. student$.ti,ab. (82596)
44. exp Students/ (39373)
45. 24 or 25 or 26 or 27 or 28 or 29 or 30 or 31 or 32 or 33 or 34 or 35 or 36 or 37 or 38 or 39 or 40 or 41 or 42 or 43 or 44 (1247090)
46. diabetes.ti,ab. (164726)
47. diabetic.ti,ab. (77850)
48. DM.ti,ab. (14606)
49. IDDM.ti,ab. (2982)
50. exp Diabetes Mellitus, Type 1/ (29286)
51. T1DM.ti,ab. (983)
52. 46 or 47 or 48 or 49 or 50 or 51 (206968)
53. 23 and 45 and 52 (1022)
54. limit 53 to (english language and yr="1996 -Current") (895)

Embase database

- - 1. diabetes.ti. (92066)
    2. diabetic.ti. (46247)
    3. DM.ti. (768)
    4. "IDDM".ti. (834)
    5. "T1DM".ti. (100)
    6. exp Diabetes Mellitus, Type 1/ (40086)
    7. 1 or 2 or 3 or 4 or 5 or 6 (149700)
    8. exp Universities/ (26143)
    9. university.m_titl. (19207)
    10. Schools/ (18169)
    11. school$.m_titl. (32110)
    12. "Academies and Institutes"/ (53716)
    13. institute.m_titl. (6921)
    14. exp Nurseries/ (881)
    15. nurser$.ti,ab. (3759)
    16. kindergarden.ti,ab. (17)
    17. kindergarten.ti,ab. (1776)
    18. polytechnic.ti,ab. (176)
    19. college.ti. (11327)
    20. (high adj1 school).ti,ab. (10906)
    21. (junior adj1 high).ti,ab. (1085)
    22. (pre adj1 school).ti,ab. (1853)
    23. pre-school.ti,ab. (1839)
    24. grade.ti,ab. (154584)
    25. (educational adj1 establishments).ti,ab. (97)
    26. (educational adj1 setting$).ti,ab. (554)
    27. (tertiary adj1 education).ti,ab. (289)
    28. (further adj1 education).ti,ab. (1105)
    29. (higher adj1 education).ti,ab. (4232)
    30. 8 or 9 or 10 or 11 or 12 or 13 or 14 or 15 or 16 or 17 or 18 or 19 or 20 or 21 or 22 or 23 or 24 or 25 or 26 or 27 or 28 or 29 (315581)
    31. exp Child, Preschool/ or exp Child/ (694676)
    32. exp Adolescent/ (563727)
    33. p*diatric.ti,ab. (140180)
    34. child$.ti,ab. (545202)
    35. adolescen$.ti,ab. (112076)
    36. toddler$.ti,ab. (3951)
    37. youth.ti,ab. (21500)
    38. teen$.ti,ab. (13801)
    39. youngster$.ti,ab. (1277)
    40. juvenile.ti,ab. (29147)
    41. pubert$.ti,ab. (16218)
    42. (young adj1 adult).ti,ab. (10993)
    43. (young adj1 person).ti,ab. (476)
    44. (young adj1 men).ti,ab. (5852)
    45. (young adj1 patients).ti,ab. (11379)
    46. (young adj1 women).ti,ab. (10420)
    47. (young adj1 people).ti,ab. (11099)
    48. (years adj2 age).ti,ab. (158078)
    49. (years adj1 old).ti,ab. (74490)
    50. student$.ti,ab. (104941)
    51. exp Students/ (33446)
    52. 31 or 32 or 33 or 34 or 35 or 36 or 37 or 38 or 39 or 40 or 41 or 42 or 43 or 44 or 45 or 46 or 47 or 48 or 49 or 50 or 51 (1441506)
    53. 7 and 30 and 52 (726)
    54. limit 53 to (english language and yr="1996 -Current") (641)

British Nursing Index database

1. exp Children/ (2055)
2. exp Adolescents/ (2186)
3. p*diatric.ti,ab. (2013)
4. child$.ti,ab. (14207)
5. adolescen$.ti,ab. (2457)
6. toddler$.ti,ab. (109)
7. youth.ti,ab. (256)
8. teen$.ti,ab. (936)
9. youngster$.ti,ab. (9)
10. juvenile.ti,ab. (64)
11. pubert$.ti,ab. (39)
12. (young adj1 adult).ti,ab. (59)
13. (young adj1 person).ti,ab. (18)
14. (young adj1 men).ti,ab. (54)
15. (young adj1 patients).ti,ab. (24)
16. (young adj1 women).ti,ab. (119)
17. (young adj1 people).ti,ab. (1333)
18. (years adj2 age).ti,ab. (88)
19. (years adj1 old).ti,ab. (94)
20. student$.ti,ab. (6485)
21. 1 or 2 or 3 or 4 or 5 or 6 or 7 or 8 or 9 or 10 or 11 or 12 or 13 or 14 or 15 or 16 or 17 or 18 or 19 or 20 (24576)
22. university.m_titl. (280)
23. Schools/ (609)
24. school$.m_titl. (1585)
25. institute.m_titl. (64)
26. nurser$.ti,ab. (126)
27. kindergarden.ti,ab. (0)
28. kindergarten.ti,ab. (5)
29. polytechnic.ti,ab. (14)
30. college.ti. (189)
31. (high adj1 school).ti,ab. (78)
32. (junior adj1 high).ti,ab. (1)
33. (pre adj1 school).ti,ab. (118)
34. pre-school.ti,ab. (118)
35. grade.ti,ab. (163)
36. (educational adj1 establishments).ti,ab. (4)
37. (educational adj1 setting$).ti,ab. (15)
38. (tertiary adj1 education).ti,ab. (3)
39. (further adj1 education).ti,ab. (34)
40. (higher adj1 education).ti,ab. (291)
41. 22 or 23 or 24 or 25 or 26 or 27 or 28 or 29 or 30 or 31 or 32 or 33 or 34 or 35 or 36 or 37 or 38 or 39 or 40 (3323)
42. diabetes.ti,ab. (3687)
43. diabetic.ti,ab. (993)
44. DM.ti,ab. (2)
45. IDDM.ti,ab. (5)
46. T1DM.ti,ab. (0)
47. exp Diabetes/ (2802)
48. 42 or 43 or 44 or 45 or 46 or 47 (4441)
49. 21 and 41 and 48 (27)
50. limit 49 to yr="1996 -Current" (27)
