# Supplementary material for: An ongoing struggle: a mixed-method systematic review of interventions, barriers and facilitators to achieving optimal self-care by children and young people with Type 1 Diabetes in educational settings
Source: BMC Pediatr. 2014 Sep 12;14:228. doi: 10.1186/1471-2431-14-228 (PMC4263204; doi:10.1186/1471-2431-14-228)
Supplement: Supplementary file 2 — Additional file 2: Additional study characteristics of included intervention studies for children and young people with T1D at educational settings.(DOC 84 KB) [file 12887_2014_1206_MOESM2_ESM.doc]

**Additional File: A2**

**Additional study characteristics of included intervention studies for children and young people with T1D at educational settings**

| Author(s) & Country | Design | Participants | Outcomes | Intervention |
| --- | --- | --- | --- | --- |
| Izquierdo et al (2009)  USA  Aims  To test the feasibility and effectiveness of telemedicine to improve care of children with T1D in schools | RCT – 2 arms  *Randomisation*  Unclear  Considered the following:  size of school (Student/teacher ratio),  the number of children with diabetes,  mean household income per school  *Blinding*  Not applicable  *ITT analysis conducted?*  No  *Length of follow-up*  1 year | 25 schools with 41 students randomised  Intervention (n=23) / usual Care (n=18)  Number completing the study not specified  *Sample characteristics*  *Mean age (years)*  Target range: Kindergarten to 8^th^ grade  Intervention: 9.74 + 2.18 years / Control 10.56 +2.5 years  *Gender:* ns    *Social Class: Median Household income*  *Intervention: £49 134 / Control: £46 528*  *Ethnicity*  All white except Intervention: African American (n-1) / Control: African American (n=3)  *Duration of diabetes*  Intervention : 4.7 years + 3.4 years / Control: 5.1 + 3.1 years  *Insulin regime*  4 injections a day or insulin pump  *Educational establishment*  *Large schools n=2*  *(>3 children with diabetes per school)*  *Intervention(n=1) / control (n=1)*  *Medium Sized Schools n=8*  *(2-3 children with diabetes per school)*  *Intervention(n=5) / control (n=3)*  *Small Schools n=15*  *(1 child with diabetes per school)*  *Intervention(n=6) / control (n=9)* | Primary  HbA1c  Others  Urgent encounters  Urgent calls  Treatment provided  Diabetes Quality of Life  Hospitalisations  Emergency department visits  Measures  Peds QL 3.0  Quality of Life - Peds QL 3.0 T1D Module  This is a 28 item instrument with five subscales: diabetes symptoms, treatment barriers, treatment adherence, worry and communication.  treatment barriers subscale at 12 months (Izquierdo et al [33], p=0.039) and Engelke, [36] p= 0.01) which assesses the extent to which children experience pain during finger prick or insulin injections, embarrassment about having diabetes, arguments about patient care, and difficulty complying with their diabetes plan.  in the treatment adherence subscale, which assesses the extent to which children experience difficulty with undertaking blood glucose tests, insulin injections, exercising, tracking carbohydrates / exchanges, wearing their medical alert bracelet, carrying a fast acting carbohydrate, or difficulty eating snacks. | Intervention in addition to usual care  A school telemedicine system with a centrally managed internet-based portal to facilitate communication between the school and the diabetes centre.  Ability to exchange graphical and tabular blood glucose measurement information with the diabetes centre nurse practitioner  Prescheduled regular monthly meetings (10-20 minutes) between the school nurse, student with or without a parent  Availability of an education curriculum  Usual care  Visits to the diabetes centre every 3 months  Communication with the school nurse and parents as needed by phone |
| Engelke et al(2008)  USA  Aims  To implement and evaluate a school-based case management program for children with chronic illnesses | Before and after study  Beginning and end of school year | Part of wider study on chronic illness  36 CHILDREN with diabetes. School nurse completed quality of life assessment on each child  Sample characteristics  *Age (years)*  Target range 5-19 not specified for children with diabetes  *Gender:* ns  *Social Class :*ns  *Ethnicity:* ns by illness type  Children were enrolled in program if they struggling academically or were having difficulty managing their illness at school | Primary  Quality of Life  Other  % meeting goals  Measures  Quality of Life - Peds QL 3.0 T1D Module | Intervention  School based case management program  Individual goals and intervention set for each child.  Training provided |
| Faro et al (2005)  USA  Aims  To conduct periodic diabetes care visits in school, with the goal of promoting optimal management of diabetes for high risk youth | Before and after study  Beginning and end of school year | 27 children with diabetes recruited  (38% Response rate)  22 nurses and health aides implementing the intervention  Sample characteristics  *Age:*  *Kindergarten to 6^th^ grade*  *Gender:* Males (55%)  *Social Class:* ns  *Ethnicity*  African American (55%) / Hispanic (25%) / White (18%) / Other (1%)  *Educational Setting*  Elementary School (17)  Middle School (4) / High School (6)  *Duration of diabetes*  6 months to 10 years / Mean 3.9 (SD = 2.7) | Primary  Self efficacy  Measures  Revised 25 item Self Efficacy scale for younger children.  15-item survey to measure parent perceptions about diabetes related experiences with schools and their satisfaction with the school’s handling of their child’s diabetes care  Investigator designed survey about usual diabetes care practices from the perspective of the by the nurses and health aides. | Intervention  Pediatric Nurse Practitioner (PNP) directed school based intervention  Monthly visits lasting 20-30 minutes  Activities included a review of home blood glucose readings, a review of school blood glucose readings and developmentally appropriate teaching focused on increasing the students’ understanding of diabetes management and improving diabetes-related problem solving skills and coping |
| Nguyen *et al* 2008  USA  Aims  To test the hypothesis that supervised BG monitoring and insulin injections at school will improve glycemic control in children and adolescents with poorly controlled T1DM. | RCT – 2 arms  Randomisation  unclear  Blinding:  Not applicable  ITT analysis conducted?  Not reported  Length of follow up  3 months  2 dropped out of control group | 36 children with high HbA1c  I (n=18) / C (n=18)  School nurse / Parents  Sample characteristics  Range 11-16 years (I) / 10-17 years (C)  Mean 14.0+1.8 years (I) / 13.3+1.7 (I)  *Gender*  Males n=7/ Females n=9 (C)  Males n=10 / Females n=10 (I)  *Social Class*: ns  *Ethnicity:* ns  *Educational Setting*:  *Duration of diabetes*  6.5 + 2.8 (3.0 to 12.7) years (C)  5.3 +4.4 (1.1 to 15.9) years (I)  *Insulin Regime:* injections | Primary  HbA1c  Adverse Events  Hypoglycaemia  Weight gain  Diabetes Ketoacidosis | Intervention  Supervision of insulin administration as a strategy to improve glycemic control  Insulin glargine injections, and periodic appropriate insulin dose adjustment  BG check and insulin injections at lunch under the direct supervision of a) the school nurse during school days and b) parents or their adult designees on the weekends and school holidays  The control group were instructed to continue their usual diabetes care and insulin regime |
| Wdowik et al (2000)  USA  Aims  To develop and evaluate an intervention program and a written curriculum guide for college students with T1D, incorporating the expanded Health Belief Model (EHBM) and social learning theory | Controlled trial  21 students recruited from university health centre(36% response rate)  10 students recruited from advertisement in local newspaper  Divided into three cohorts  21 students (100%) completed the study  Qualitative and Quantitative evaluations were conducted before and after the study, and at a 3 month follow up | Sample characteristics:  Age  Mean 22 years / Actual range: 18 to 27 years  Gender: Males (52%)  Social Class: ns  Ethnicity: ns  Educational Setting  Freshman (19%) / Juniors (28%)  Seniors (33%) / Graduate school (20%)  Duration of diabetes  Mean : 12 years / Range 4 to 24 years  Control  10 students recruited from a different university population through advertisement  1 student recruited from class  10 students (91%) completed the study  Sample characteristics  Age: Mean 24.5 years  Gender: Males (70%)  Social Class: ns  Ethnicity: ns  Educational Setting  Freshman (10%) / Sophomore (10%)  Junior (10%) / Seniors (60%)  Graduate school (10%)  Duration of diabetes: 12.9 years | Primary  Knowledge  Obtaining and knowing a HbA1c result  Values, attitudes and behaviours  Measure  12 item investigator designed measure to assess knowledge.  82-item investigator designed questionnaire to assess attitudes  8 item investigator designed questionnaire on a 5 point Likert scale to assess diabetes self care behaviours and outcomes | Intervention  “Control on Campus”  Which is a 92 page comprehensive guide that provides up to date information on diabetes management which was delivered over 3 sessions ;lasting 2 hours over 3 consecutive weeks  Attendance at one individual session with the group facilitator, a registered dietician / certified diabetes educator. (Only 505 attended |

**Additional study characteristics of included intervention studies for school personnel working with children and young people with T1D**

| Husband et al 2001  Canada  Aims  To determine of a CD-Rom teaching tool accomplishes the following:  Increases teachers’ knowledge about T1D in children  Increases teachers’ knowledge about hypoglycaemia in children  Increases teachers’ confidence in responding to the needs of children with T1D | RCT – 2 arms  Randomisation  Not clear  Conducted by school  *Blinding:*  Not applicable  *ITT analysis conducted?*  No  *Length of follow-up*  *7 weeks* | 44 Teachers recruited  (39% response rate)  37 completed the study  Experimental group n=17  Control Group n=20  Sample characteristics of children with T1D not specified  *Educational establishment*  Elementary school  *Experience of children with T1D*  *Experimental Group:100%*  *Control Group:100%* | Primary  Knowledge  Measure  17 item multiple choice questionnaire developed by the researchers  Other  Confidence  Measure  4 –item questionnaire with a 5 point Likert scale developed by the researchers | Intervention  A CD-Rom teaching tool entitled *Type 1 Diabetes in Children: A Passport to knowledge* |
| --- | --- | --- | --- | --- |
| Siminerio and Koerbel  (2000){Siminerio, 2000 #3836}  USA  Aims  To assess knowledge levels and needs of school personnel and to determine the effectiveness of a comprehensive diabetes education program that highlights the current trends and associated advanced technologies in the care of children with diabetes | Before and after study  Immediately before and after intervention | 156 school personnel recruited from six school districts  (Response rate not specified)  Sample characteristics  School Nurses (ns)  School Teachers (ns)  Elementary (ns)  Middle (ns)  High (ns)  Previous experience diabetes  Yes – student with diabetes (38%)  Yes – family member / friend / other teacher (42%)  No (20%) | Primary  Knowledge  Measure  10 item investigator designed  3 additional open ended questions | Intervention  The program was entitled the `5 Cs of diabetes', and highlighted new information in the areas of cause, classification, complications (acute and chronic), care and cure.  The 5 Cs program was presented in a lecture format that lasted 1 to 1 ½ hours.  Delivered by two certified diabetes educators from the Children's Hospital of Pittsburgh |
| Cunningham and Wodrich 2006  USA  Aims  To examine the effect of providing teachers with varying levels of information about T1D  Does providing teachers with health information influences the proportion of well-targeted (i.e disease related) classroom accommodations and teachers’ confidence in their accommodation?  Do teachers’ demographic variables (e.g level of experience, background in special education, acquaintance with someone with T1DM) alter the value of providing health information in creating disease related accommodation’s.  Does health information influence teachers’ desire for supplemental information that they need to help students with T1D  From whom is such supplemental information sought by teachers and does providing more health information alter to whom teachers turn for this information. | Analog experiment (allocated)  Survey (administered during staff meetings)  The three levels of the independent variable (T1DM information level) were:  *no disease information*,  *basic disease information*,  *basic disease information + classroom implications*.  In each condition, basic student data also were provided that comprised information about a hypothetical student’s school-attendance history, past school performance, personality characteristics, and T1M diagnosis | 90 recruited (Response rate not specified)  Sample characteristics  Not linked into specific children with T1D  School characteristics  Elementary (n=4)  Staff Characteristics  Regular Teachers – 70%  Special Education Teachers – 30%  Experience of children with T1DM*:*  *26% k*new a student with T1DM  19% knew a friend with T1DM  23% had a relative with T1DM  26% knew a student with T1DM  Of participants who knew someone with T1DM, 33% knew them quite well or very well. | Primary  Confidence and knowledge  Measure  Investigator designed  tool | Intervention  Teachers were provided with varying levels of information about T1D with each participant received only one T1D information level, creating a between-subjects design  Level one (no disease information) provided teachers one page of information on general education unrelated to T1D. The content of this page focused on current issues in education, including the challenges of providing all children equal access to education and educating atypical students. T1D was not mentioned in this literature  Level two (basic disease information) provided a one-page description of T1D and its psychological implications  Level three (basic disease information_classroom implications) provided a one-page description of T1D, its psychological implications, and examples of well targeted classroom accommodations for children with T1D |
| Wodrich 2005  USA  Aims  To investigate the effects of disclosing information about T1D with implications for classroom learning and behaviour | Analog experiment (random assignment)  Analog school situation with continuing education and pre service teachers.  Randomly assigned to one of three levels of health information (no knowledge, diagnosis only, diagnosis and facts) and provided and a list of sources to which the problematic classroom performance may be attributed | 122 recruited(Response rate not specified)  Sample characteristics  Not linked into specific children with T1D  School characteristics  Elementary (80%)  Middle/Junior High School (10%)  High School (10%)  Staff Characteristics  Continuing Education teachers (n=56)  Pre-service teachers (n=66)  Experience of children with T1D  N/A | Primary  Confidence and knowledge  Measure  Investigator designed  tool | Intervention  Each participant learned facts from two sources about one hypothetical elementary student who had T1D  The first information source was a Cumulative Folder that summarized information about the student’s family (e.g.  family size and parents’ vocations), health (differed across the three health information levels (See Cunningham and Wodrich, educational background, educational records (e.g., attendance records, report card grades), and educational environment (e.g. class size, curriculum material used).  The second source of information was a video of a teacher and a school psychologist discussing the student.  Teachers randomly assigned to one of three levels of health information (no knowledge, diagnosis only, diagnosis and facts) and provided and a list of sources to which the problematic classroom performance may be attributed. |
| Bullock *et al* 2002  USA  Aims  To determine if attendance at specific continuing education programs increased competence of school nurses who enrolled and completed the programmes | Cohort study  Survey (postal) | 561 (36% response rate)  Sample characteristics for diabetes program area (n=537)  School nurses who had participated in a continuing education program (n=120)  School nurses who had not participated in continuing education program (n=417). Includes those who did not enrol and those who enrolled and did not complete course  Registered nurses (79%)  Licensed practical nurses (18%)  Masters prepared school nurses (3%)  Years of experience: Mean of 6.9 years  Experience of children with T1D*:*ns  Number of children with T1D*:*ns | Primary  Competence  Measure  35 forced choice item, investigator designed measured on a Likert scale:  1= *perceived competence in practice 75–100% of the time*; 2= *perceived competence 50–75% of the time*; 3 = *perceived competence 25–50% of the time*; 4= *perceived competence 0–25% of the time*.  Therefore, a lower mean score indicated a higher competence level | Intervention  Intervention those attending the CEP on diabetes management  No further details provided  Control those non attending the CEP on diabetes management |
| Bachman and Hsueh (2008)  USA  Aims  To develop and evaluate an online continuing education program to educate school nurses in how to manage care for children with diabetes in schools using current practice principles outlined in *Diabetes Management in the School Setting. A resource guide for School Nurses* | Program evaluation | 15 recruited (79% response rate)  Sample characteristics  School nurses who had participated in an on-line continuing education programme for diabetes  No further details presented | Primary  Perceived confidence  Measure  Survey  Evaluation of online course  Investigated whether lesson objectives met  Open ended questions to rate effectiveness of each lesson and make suggestions for change | Intervention  On-line continuing education programme which consisted of three lessons. Lesson 1 was an overview of diabetes in children and an update on diabetes management in the school setting. Lesson 2 covered managing students with insulin pumps. Lesson 3 discussed the role of the school nurse in managing children with diabetes  Course participants used the revised *Diabetes Management in the School Setting: A Resource Guide for School Nurses* which includes (a) first steps in developing a diabetes program in the school; (b) an overview of diabetes; (c) nutrition guidelines for diabetes; (d) exercise and exercise safety tips with diabetes; (e) insulin, insulin therapy, insulin pumps, and troubleshooting insulin pumps; (f) monitoring glucose levels; (g) emergency action plans and sample tools needed to implement emergency action plans; (h) health management (eye, oral, foot, immunizations); (i) references with links to local and national resources; (j) a survey to elicit feedback about the manual; and (k) forms and handouts that can be adapted easily by the school nurse, printed out, and distributed as appropriate |
