# Supplementary material for: An ongoing struggle: a mixed-method systematic review of interventions, barriers and facilitators to achieving optimal self-care by children and young people with Type 1 Diabetes in educational settings
Source: BMC Pediatr. 2014 Sep 12;14:228. doi: 10.1186/1471-2431-14-228 (PMC4263204; doi:10.1186/1471-2431-14-228)
Supplement: Supplementary file 3 — Additional file 3: Study characteristics for non-intervention studies (Stream 2).(DOC 158 KB) [file 12887_2014_1206_MOESM3_ESM.doc]

| **Study / Country / Aims** | **Sampling and Sample Characteristics** | | |
| --- | --- | --- | --- |
| **Children and Parents** | | | |
| Nabors *et al* (2003), USA  To assess children’s and young adolescents perceptions of supportive behaviour by nurses, teachers and friends that allow them to improve their diabetes management at school | 105 children recruited (RR not specified)  Recruited at day and summer camp  Age (years):  Mean 10.11 (S.D. 2.2) / Range 6–14.6  Gender: Female (43%)  Social class: ns | Ethnicity:  Caucasian (94%) / African American (6%)  Insulin regime: ns  Educational establishment: ns  Duration of diabetes (years):  Average about 4 years / 4 months to 11 years | |
| Bodas *et al* (2008), Spain  To improve awareness of the needs, within school settings of children and adolescents with T1D based on information provided by the students | 414 children recruited (RR not specified)  Recruited at summer camps  Age (years): Target range 6-16 years  Gender: Female (51%)  Social class: ns | Ethnicity: ns  Insulin regime: ns  Educational establishment: Primary School (47%) / Middle School (48%) / High School (5%)  Duration of diabetes (years): ns | |
| Peters *et al* (2008), USA  To examine diabetes-related teacher victimization  To examine the association between teacher victimization, adherence and metabolic control  To examine whether these relations differ between children and adolescents | 167 children recruited (RR not specified)  Recruited from a paediatric clinic of a major University.  Age (years):  Mean 12.8 (S.D 2.5)  Target range 8-17  Gender: Females (64%)  Social class: ns | Ethnicity:  White (80.2%) / African American (13.8%)  Hispanic (2.6%) / Other (2.4%)  Insulin regime:  Excluded if they used a pump or if serious psychopathology in the child or parent.  Educational establishment: ns  Duration of diabetes (years): At least 1 year | |
| Lehmkuhl and Nabors (2008), USA  To assess children’s perceptions of their satisfaction and support from school nurses, teachers, and friends in their classrooms as well as the types of support they needed from each group | 58 children recruited (RR not specified)  Recruited at summer camp  Age (years):  Mean 11.5 ( S.D 1.0 ) / Target range 8-14  Gender: Females (47%)  Social class: ns | Ethnicity: Caucasian (100%)  Insulin regime: ns  Educational establishment: ns  Duration of diabetes (years):  Mean 6.7 (S.D 2.9) / Range 1 – 11 | |
| Tang and Ariyawanska 2007, UK  To identify the difficulties that young people with diabetes may encounter while at school | 11 children recruited (55% RR)  11 parents recruited (55% RR)  Recruited from diabetes’s clinic  Age (years): Target range 12-16  Gender: ns  Social class: ns | Ethnicity: ns  Insulin regime: ns  Educational setting: Secondary (100%)  Duration of diabetes (years):  Diagnosed under the age of 10 (n=8) | |
| Wang *et al* (2010), Taiwan  To obtain an initial understanding of school –based lived experiences of adolescents with T1D | 2 recruited (RR not specified)  1 Female (age 14) injections 3 times a day. Been diagnosed for 1 year.    1 Male (age 15) pump. Been diagnosed for 6 years. | | |
| Newbould *et al* (2007), UK  To examine the experiences and concerns of young people and their parents in the management of medication for asthma or diabetes whilst at school | 26 children recruited (30% RR)  26 parents recruited (30% RR)  Recruited from GP practices  Age (years): Mean 11.7 / Target range 8-15  Gender: Female (54%)  Social class: Home Owners (81%) | | Ethnicity: White (100%)  Insulin regime: ns  Educational setting:  Primary (n=12) / Lower (5-8 yrs) (n=1)  Secondary (n=13)  Duration of Diabetes (years): Mean 4.8 |
| MacArthur 1996, UK  Looked at the practice and attitudes of local children who were taking pre lunch insulin and injections at school | 15 children recruited (88% RR)  Recruited from diabetes clinic  Age (years): Target range 10-16  Gender: ns  Social class: ns | | Ethnicity: ns  Insulin regime: pre lunch injections at school  Educational establishment: Secondary n=11)  Duration of diabetes (years):ns |
| Clay 2008, USA  To examine problems with medication administration in the school by | 75 children recruited. (41% RR)  75 parents recruited. (41% RR)  Recruited from routine clinic appointments  Age (years):  Mean 13.3 (S.D. 2.8) / Target range 8-18  Gender: Female (31%)  Social class: ns | | Ethnicity: ns  Insulin regime: ns  Educational establishment: Kindergarten-3^rd^ grade (11%) / 4^th^ -6^th^ grade (25%) / 7^th^ - 8^th^ grade (23%) / 9^th^-12^th^ grade (41%)  Duration of diabetes (years):ns |
| Schwartz *et al* (2010), USA  To evaluate the experience of children and adolescents with T1D in school | 80 children recruited (62% RR)  80 parents recruited (62% RR)  Recruited from University Medical Associates Diabetes/Endocrine Center  Age (years):Target range 5-12  Gender: ns  Social class: ns | | Ethnicity: ns  Insulin regime: ns  Educational establishment:  Kindergarten to 12^th^ grade  Duration of diabetes (years): ns |
| Hema *et al* (2009), USA  To investigate the daily stressors and coping responses of children and adolescents with T1D | 52 recruited (RR not specified)  Recruited at summer camp  Age (years):  Mean 13.02 (S.D. 2.66) / Target range 8-18  8-12 years (n=19) / 13-18 years (n=33)  Gender: Female (65%)  Social class: ns | | Ethnicity:  Caucasian (96%) / Other (ns)  Insulin regime: ns  Educational establishment: ns  Duration of diabetes (years): 4.78 (S.D 3.46) |
| Peyrot 2009  Part of the DAWN Youth WebTalk Study  Brazil, Denmark, Germany, Italy  Japan, The Netherlands, Spain, USA  To gain understanding of the challenges and issues facing young people with diabetes and those with responsibility | 1905 children^a^ recruited (RR not specified)  4099 parents^b^ recruited (RR not specified)  Part of the DAWN Youth WebTalk Study.  Age (years):  ^a^Mean 21.3 (S.D. 2.4) / Target range 18-25  ^b^ Mean 10.5 (S.D. 4.2) / Target range 0-16  Gender: ^a^Female (60%) / ^b^ Female (50%)  Social Class: ^a &b^ns | | Ethnicity: ^a b^ns  Insulin regime: ^a&b^ns (T1D: ^a^94.1% /^b^98.1%  Educational establishment: ^a&b^ ns  Duration of diabetes (years):  ^a^Age at diagnosis 12.2+2.3 /  ^b^ Age at diagnosis: 6.3+3.9 |
| Carroll and Marrero 2006, USA  To explore the perceptions of how diabetes influences adolescents’ perceptions of quality-of-life in general and their relationships with parents, peers, school and their physician | 31 children recruited (RR not specified)  Recruited from physicians’ offices  Age (years):  Mean 14.9 / Target range 13-18  13-14 (45%) / 15-16 (35%)  17-18 (20%)  Gender: Females (42%) | | Ethnicity: White (90%)/African American (10%)  Educational establishment: ns  Insulin regime: Injections (52%)/Pump (48%)  Duration of diabetes (years):  Mean 6.6 (Range 6-14) / 0-3 yrs (26%) / 3-6 yrs (29%) / 6-9 yrs (16%) / >10 years (29%) |
| Waller *et al* (2005), UK  To seek the views of children and adolescents with T1D and their parents regarding the acceptability and design of a new diabetes education programme called the DAFNE | 24 children recruited (27% RR)  29 parents recruited (31% RR)  Recruited from diabetes clinic lists  Age (years):  Mean 13.07 (S.D 1.59) / Target range 11–16  Gender: Female (50%)  Social class: ns | | Ethnicity: ns  Insulin regime: Injections (100%)  Educational establishment: ns  Duration of diabetes (years):  Had been diagnosed for at least 1 year |
| Hayes-Bohn *et al* (2004), USA  Diabetes care at school from the perspective of adolescents with T1D and their parents | 30 children recruited (RR not specified)  30 parents recruited (RR not specified)  Recruited from outpatients department  Age (years): Mean 17.3 / Target range 13-20  Gender: Females (100%)  Social class: ns | | Ethnicity: Caucasian (84%)  African American (13%) / Hispanic (3%)  Insulin regime: ns  Educational establishment: ns  Duration of diabetes (years):Inclusion criteria diagnosed > 1 year / Average 7.7 (S.D. 4.1) |
| Wagner *et al* (2006), USA  To investigate the relationships among perceived school experiences, diabetes control and quality-of-life | 58 children recruited (48% RR)  58 parents recruited (48% RR)  Recruited at a summer camp  Age (years):  Mean 12 (S.D 1.9) / Target range 8-15  Gender: Female (55%)  Social class: ns | | Ethnicity*:* European American (98%)  Insulin regime*:* Pump or MDI (100%)  Educational setting: Public schools (90%)  Duration of Diabetes (years):  Mean 5.3 years (S.D. 3.1 years) |
| Amillategui *et al* (2009), Spain  To identify the special needs of children with T1D at primary school taking into account the perceptions reported by parents, children and teachers | 152 children^a^ recruited (35% RR). 167 parents^b^ recruited (39% RR). Recruited from paediatric unit of nine public hospitals  Age (years):  ^a^Mean 10.68 (S.D 1.92) / Target range 6-13  6-9 (29%) / 10-13 (71%)  b Mean 10.37 (S.D 2.15)/Target range 6-13  6-9 (35%)/ 10-13 (65%)  Gender: ^a^Female (48%) / ^b^ Female (50%) | | Social class: ^a &b^ns  Ethnicity: ^a &b^ns    Insulin regime: ^a &b^ ns  Educational establishment: ^a &b^Primary (100%)  Duration of diabetes (years):  ^a^Mean 4.25 (S.D 2.90) / <3 years (33%)  3-6 years (45%) / >6 years (22%  ^b^ns |
| Barnard *et al* (2008), UK  To identify key components of quality-of-life and assess the impact of insulin pump therapy on children / adolescents with type 1 diabetes and their parents | 15 children recruited (14% RR)  17 parents recruited (15% RR)  Registered on the Roche Diagnostics insulin pump user customer database  Age (years):  Mean age 12.07 (S.D. 2.71) / Target range 9-17  Gender: ns  Social class: ns | | Ethnicity: ns  Insulin regime: Pump (100%)  Educational establishment: ns  Duration of diabetes (years):  Mean 6.67 (S.D. 2.42 ) / Range 2 – 12 |
| Low *et al* (2005), USA  To explore psychosocial issues related to insulin pump use (continuous subcutaneous insulin infusion (CSII)) in youth aged between 11 and 18 years | 18 children recruited (57% RR)  21 parents recruited (57% RR)  Recruited through diabetes camps and a regional paediatric endocrinology practice.  Age (years):  Mean age 13.9(S.D. 2.2) / Target range 11-18  Gender: Female (50%)  Social class: Family Income $  0-25,000 (4.4%) / 25,000-50,000 (11.1%) / 50,000-75,000 (33.3%) /  Over 75,000 (50%)2 | | Ethnicity: Caucasian (100%)  Insulin regime: Pump (100%)  Educational establishment: ns  Duration of diabetes (years):  Mean 6.1 (S.D. 2.5 ) |
| Wilson and Beskine 2007, UK  To examine how children with diabetes are managing their condition in the school setting using pump therapy and multiple daily injections | 73 parents recruited (64% RR)  Recruited via a survey on the UK CWD website  Age (years):  Under 5 (11%) / 5-11 (55%)  Over 12 (34%)  Gender: Female (59%)  Social class: ns | | Ethnicity: ns  Insulin regime: Pump (60%) / MDI (40%).  Educational establishment: ns  Duration of diabetes (years):ns |
| Amillategui *et al* (2007), Spain  To identify the special needs of children with T1D in schools from the parents’ | 499 parents recruited (RR not specified)  Recruited from one paediatric outpatient clinic  Age (years): Target range 3- 8 /  3-6(12%) 7-10 (26%) / 11-14 (38%) /  15-18 (24%)  Gender: Female (45%)  Social class: ns | | Ethnicity: ns  Insulin regime: ns  Educational establishment: ns  Duration of diabetes (years): ns |
| Pinelli *et al* (2011), Italy  To determine how Italian parents and school personnel of 6-13 year old children with T1D manage during school hours, including insulin administration, management of hypoglycaemia, and glucagon use | 220 parents recruited (100% RR)  Recruited from 15 diabetes units  Age (years): Mean 10 / Target range 8-13  Gender: Female (41%)  Social class: ns | | Ethnicity: ns  Insulin regime: ns  Educational establishment:  Elementary (65.6%) / Middle (46%)  Duration of diabetes (years):  Mean 5 (Range 1 to 12 years) |
| Hellems and Clarke 2007, USA  To determine which school personnel currently assist students with insulin administration and management of hypoglycaemia and to determine whether these students are being cared for in a safe manner | 185 parents recruited (RR not specified)  Recruited from diabetes clinics  Age (years): Target range 5-18  Gender: ns  Social class: ns | | Ethnicity: ns  Insulin regime: ns  Educational establishment: Elementary(n=58)  Middle (n=60) / High (n=67)  Duration of diabetes (years): ns |
| Jacquez *et al* (2008), USA  To investigate parent reports of the diabetes care support their children receive in school, their concerns about diabetes management in school, and their knowledge about diabetes management in school, and their knowledge of federal laws that protect children with diabetes | 309 parents recruited (92% RR)  Recruited from 2 outpatient diabetes clinics  Age (years):  Mean 11.83 (S.D. 3.70) / Target range 4-19  Gender: ns  Social class:  Mean Hollingshead socio-economic status score =40.94 (S.D. 10.47) | | Ethnicity: Hispanic White (61%) / Non Hispanic white (19%) / African/Caribbean American (19%)  Other (1%)  Insulin regime:  T1D: Pump (29%) / Injections (71%)  T2DM: Injections (38%) / Oral Medication (62%)  Educational establishment: ns  Duration of diabetes (years): 4.37 (S.D. 3.62) |
| Lewis *et al* (2003), USA  To identify obstacles to good control of diabetes in the school setting and document the level of support available in various school districts serving the clinic patient population | 47 parents (RR not specified)  Recruited from diabetes clinic  Age (years): ns  Gender: ns  Social class: ns | | Ethnicity: ns  Insulin regime: ns  Educational establishment: Elementary (40.4%) / Middle (29.8%) / High (29.8%)  Duration of diabetes (years): ns |
| Yu *et al* (2000), USA  To characterize the academic and social experiences of children with diabetes | 66 parents recruited (RR not specified  Recruited from paediatric endocrinology unit  *Early onset diagnosed at <5 yrs (n=31)*  Age (years): Mean 12.7  Gender: Female (52%)  Social class: ns  Ethnicity: White (94%)  Insulin regime: ns  Educational establishment: ns  Duration of diabetes (years): Mean 9.7 | | *Late onset – diagnosed after 5 yrs (n=35)*  Age (years): Mean 12.6  Gender: Female (51%)  Social class: ns  Ethnicity: White (97%)  Insulin regime: ns  Educational establishment: ns  Duration of diabetes (years): Mean 4.2 |
| Lin *et al* (2008), Taiwan  To explore the essential structure of mothers’ life experience when helping their first-to-third grade children with T1D make adjustments at school | 12 mothers (RR not specified)  Recruited from children’s day clinic  Age (years): mean 8.4. Range 7.3- 9.2  Gender: Female (67%)  Social class: ns | | Ethnicity: ns  Insulin regime: injections (100%)  Educational establishment: 1^st^ – 3^rd^ grade  Duration of diabetes (years): 3.3 years (2 to 5.8) |
| **Students** | | | |
| Ramchandani *et al* (2000), USA  To assess changes in diabetes management and control that occurs in the transition from high school to attending college away from home. | 51 students recruited (31% RR)  From 5 different hospital diabetes centres  Data used for 42 participants  Age (years):  Mean 20.1 (S.D. 1.6) / Range 18.4- 25.7  Gender: Female (64%)  Social Class: ns  Ethnicity: ns | | Insulin regime: Injections (92.8%)/Pump (7.2%)  Educational establishment:  College or University (100%)  Year of study:  Freshman (36.6%) / Sophomore (12.2%)  Junior (26.8%) / Senior (14.6%)  Graduates (9.8%)  Duration of diabetes (years): ns |
| Balfe 2007a, 2007b,  Balfe and Jackson 2007  Balfe 2009a, 2009b, UK  To explore the narratives of practice of young university students with T1D | 17 students recruited from across 5 university health centres. (11% RR)  Age (years):  Actual range 18-25 years  Gender: Female (65%)  Social class: ns  Ethnicity: White (100%) | | Insulin regime: Injections (94%)/ Pump (6%)  Educational establishment: University (100%)  Year of study:  1^st^ year (n=4) / 2^nd^ year (n=9)  3^rd^ year (n=1) / Post graduate student (n=3)  Duration of diabetes (years):  at least 1 year, (n=14 > 6 years) |
| Wdowik et al (2001), USA  To determine relationships between constructs of the Expanded Health Belief Model and to identify characteristics of college studiers who successfully manage their diabetes | 98 students recruited (85% RR)  Recruited from 22 college health providers  Age (years): Mean 24.4 (S.D 7.4)  Gender:*:* Female (57%)  Social class: ns  Ethnicity: White (83%) /  Black / African American (4%)  Hispanic / Mexican American (6%) /  Not stated (6%) | | Insulin regime:  Not T1D (n=2)  Educational establishment:  College students (100%)  Year of study: ns  Duration of diabetes (years):  Mean 11.8 (S.D 6.9) |
| Wdowik 1997, USA  To identify factors that affect the ability and motivation of college students to engage in appropriate self-care behaviours for successful management of diabetes | 10 students recruited to focus groups (32% RR)  Used the student health services or seen advertisement in college newspaper  Age (years):  Target range 18–35 / only 2 over 24 years  Gender: Females (80%)  Social class: ns  Ethnicity: ns  Insulin regime: ns  Duration of Diabetes (years): 3 months-13 yrs  Educational establishment: College (100%)  Year of study*:* ns | | 15 recruited to telephone interviews (75% RR)  Attended a pre-college workshop at local diabetes centre who were then from 9 colleges across 7 different states  Age (years):  Target range 19-22 years  Gender: Females (60%)  Social class: ns  Ethnicity: *ns*  Insulin regime: ns  Duration of diabetes (years): 3-17 years  Educational establishment:  College (100%)  Year of study: ns |
| Geddes *et al* (2006), UK  To examine the clinical characteristics of, and diabetes management provided for, young people with T1D in tertiary education | 55 case notes of students (37% RR)  Referred to one hospital over a 10 year period  Age (years): Target range 18-24  Gender: ns  Social class: ns  Ethnicity: ns | | Insulin regime:  Basal-bolus (35%)  Insulin 3 x a day (3%)  Insulin 2 x a day (62%)  Educational establishment: degree level study  Year of study: ns  Duration of diabetes (years): Over 1 year |
| Ravert 2009, USA  To examine the use of nine common alcohol management strategies among college undergraduates with diabetes in order to determine which strategies predicted alcohol consumption and consequences | 450 respondents who had competed a graduate survey who indicated that they had diabetes, were less than 26 years of age and who had undergraduate status from 123 North American post secondary institutions.  Age (years):  Mean 20.3 (S.D. 1.6) / Target range 18-25  Gender: Female (68%)  Social class: ns  Ethnicity: White (79.6%) / Black (5.1%)  Hispanic (5.6%) / Asian (6.9%)  Indian (2.4%) / Other (4.7%) | | Insulin regime:*:* ns  Educational establishment:  College undergraduates  Year of study:  1^st^ year (28.2%) / 2^nd^ year (24.7%)  3^rd^ year (24.7%) / 4^th^ year or more (22.4%)  Duration of diabetes (years): ns |
| Wilson 2010, UK  To explore the experiences of young people managing their diabetes at college of university | 23 students recruited (no details provided)  RR not specified  Age (years): Actual range 17-19  17 (30%) / 18 (44%) /19 (26%)  Gender: Female (52%)  Social class: ns  Ethnicity: ns | | Insulin regime: ns  Educational establishment:  College (30%) / University (70%)  Year of study: ns  Duration of diabetes (years):  8 (44%) / 9 (30%) / 10 (26%) |
| Miller-Hagan and Janas 2002, USA  To explore how college students with diabetes perceive and manage alcohol consumption | 15 students recruited (RR not specified)  Advertisements placed in one large North-eastern University.  Age (years):  Mean 22.4 / Actual range 18-40  Social class: ns  Gender: Female (73%)  Ethnicity:  White (87%) / Black (6%) / Asian (6%) | | Insulin regime: Type: 1 (n=11 /Type 2 (n=4)  Pump (n=4) / Oral (n=3)  Educational establishment:  Sample included graduate students  Year of student: ns  Duration of diabetes (years):  8.1 (range 3.5 months to 17 years) |
| Eaton *et al* (2001), UK  To contact students with diabetes at the University of Leeds to ascertain their, alcohol, smoking and exercise habits and to explore their views on diabetes and factors which influence their ability to maintain glycaemic control | 22 recruited. (47% RR)  Registered at one university medical practice.  8 selected for interview  Age (years): Mean 20 / Actual range 19-21  Gender*:* ns  Social class: ns | | Ethnicity: ns  Insulin regime: ns  Educational establishment: University (100%)  Year of study: ns  Duration of diabetes (years):  Mean age at diagnosis 12.6 (range 6-20) |
| **School Teachers** |  | |  |
| Amillategui *et al* (2009), Spain  To identify the special needs of children with T1D at primary school taking into account the perceptions reported by parents, children and teachers | 111 recruited (26% RR)  Teachers of children with T1D attending the paediatric units of nine public hospitals.  Experience of children with T1D: Yes (100%)  School characteristics: Primary (100%)  Staff Characteristics: Teachers (100%) | | Details of children with T1D:  Age (years):  Mean 10.08 (S.D. 2.26)  6 to 9 (38%) / 10 to 13 (62%)  No further details specified |
| Greenhalgh 1997, UK  To assess school teachers knowledge of insulin dependent diabetes in school children aged 5 to 16 years | 85 recruited. (60% RR)  School teachers of children who attended a diabetes clinic a local hospital  Experience of children with T1D: Yes (96%)  School characteristics:  Primary (n=22) / Secondary (n=16) | | Staff characteristics:  Primary: head &, child class teachers (n=25)  Secondary: science/biology & PE teachers (n=23)  Head of year and form teachers (n=37)  Details of children with T1D:  Not provided |
| Bowen 1996, UK  To discover whether the teachers had the training to cope with the health related emergencies which, may arise with children who have special needs | 30 recruited (83% RR)  Experience of children with T1D:  Taught a child with diabetes (20%)  School characteristics:  Primary (n=4) / Secondary (n=1) | | Staff characteristics:  Primary (43%) / Secondary (57%)  Sample characteristics:  Not linked into specific children with T1D |
| Alnasir and Skerman 2004, Bahrain  To study awareness about common health problems in Bahrain  Latif Almasir 2003, Bahrain  To assess the Bahrani school teachers’ knowledge of diabetes | 1140 recruited (91% RR).  All Bahraini teachers of all disciplines in 49 randomly selected schools  Experience of children with T1D: Not reported  School characteristics: ns | | Staff characteristics:  Primary Teachers (45%)  Intermediate Teachers (25%)  Secondary Teachers (30%)  Details of children with T1D:  Not linked into specific children with T1D |
| Gormanous *et al* (2002), USA  To determine the levels of knowledge about diabetes mellitus among Arkansas public elementary school teachers | 463 recruited (64% RR)  Details of children with T1D:  Not linked into specific children with T1D  School characteristics: Elementary (n=27)  Staff characteristics: Kindergarten to 6^th^ grade | | Experience of children with T1D:  Student with diabetes in classroom (7%)  Not aware if students with diabetes were in their classrooms (24%)  Family member or close friend (42%) |
| Tahirovic 2007, Bosnia and Herzegovina  To investigate how far physical education teachers from elementary school understand diabetes and are trained in its management and in the treatment of diabetes emergencies according to their understanding | 83 recruited (RR not specified)  All schools within the region included.  Experience of children with T1D:  G1: Teachers in whose schools were attended by at least 1 pupil suffering from T1D  G2: Teachers in whose schools there were no pupils suffering from T1D | | School characteristics: Elementary (n=83)  Staff characteristics:  Physical Education Teachers  Details of children with T1D:  Not linked into specific children with T1D |
| MacArthur 1996, UK  Looked at the practice and attitudes of local children who were taking pre lunch insulin and injections at school | 11 recruited (82% RR)  Teachers of children with T1D recruited from diabetes clinic.  Experience of children with T1D:  All children in schools who took who took pre lunch injections at school  School characteristics: Secondary (n=9) | | Staff characteristics:  Head teacher (n=4) / Form teacher (n=3)  Deputy Head (n=2) / Head of year (n=1)  School Nurse jointly with deputy head (n=1)  Details of children with T1D: ns  Age (years): 10-16  No further details specified |
| Boden *et al* 2011, UK  To examine the concerns of primary school staff working with children with T1D and their parents, and to relate these views to the views of health care professionals working with school personnel | 22 recruited (88% RR)  25 primary schools were identified as having a child with diabetes in the school either currently or who had left very recently  Experience of children with T1D:  No (9%) / Current (46%) / In directly (9%)  Previous (previous year) (27%)  Previous (no longer in school) (9%) | | School characteristics: Primary (n=13)  Staff characteristics:  Head teachers, teachers and teaching assistants, who had managed, or could potentially manage, children with diabetes  Details of children with T1D:  Not details provided |
| Nabors *et al* (2008), USA  To assess special education and regular education teachers’ perceptions of their knowledge about and confidence in meeting the academic and social needs of children with chronic medical conditions (including diabetes) | 247 recruited from 15 elementary schools in a Midwestern city. (RR not specified)  Experience of children with T1D: ns  School characteristics: Elementary (n=15)2 | | Staff characteristics:  Special Education Teachers (22%)  Regular Teachers (78%)  Details of children with T1D:  Not linked into specific children with T1D |
| Lewis *et al* (2003), USA  To identify obstacles to good control of diabetes in the school setting and document the level of support available in various school districts serving the clinic patient population | 222 schools in 3 counties were randomly selected to participate in the study.  65 responded (29% RR)  Experience of children with T1D:  80% schools had a child enrolled with diabetes  4 schools did not know if they had students with diabetes enrolled | | Staff characteristics: ns  School characteristics:  Elementary (27.6%) / Middle (33.8%)  High (35.3% ) / Combined Middle/High (3.1%)  Details of children with T1D:  Not linked into specific children with T1D |
| Rickabaugh and Salterelli 1999, USA  To explore the attitudes and reported behaviours of participants concerning diabetes and exercise guidelines | Participants included 25 children with T1D and 28 of their parents and 32 physical education teachers. Recruited from three across three states. (RRs not specified)  Experience of children with T1D:  Had taught on average 4 children with T1D (S.D=0.9).  Had an average of less than one incidence of hypoglycaemia (Mean 0.94 events, S.D. 0.08) in their classes per year | | School characteristics: ns  Staff characteristics:  Physical Education Teachers (100%)  Details of children with T1D:  Age (years): Mean 12.1 (S.D 0.7)  Gender: Female (48%)  Ethnicity: ns  Social Class: ns  Duration of diabetes (years): 5.0 (S.D 0.6) |
| Chmiel-Perzynska *et al* (2008), Poland  To evaluate the knowledge of primary school teachers in the Lubelskie Province, Poland, about hypoglycaemia and to determine educational needs necessary to ensure that children with diabetes are properly dealt with by their teachers | 200/753 (26%) of teachers had previously responded to a survey. Of these 52 were currently teaching or had taught a child with diabetes. (27% RR)  Experience of children with T1D:  Currently teaching / had taught | | School characteristics: Primary (100%)  Staff characteristics: Teachers (100%)  Details of children with T1D:  Not linked into specific children with T1D |
| **School Healthcare Personnel** |  | |  |
| Fisher 2006, USA  To measure school nurses’ perceived self-efficacy in providing diabetes care in education to children | 70 school nurses recruited (61% RR)  From a sample of 115 schools in a sub urban are in New England. RN (100%)  Years of experience:  1–5 (36%) /6–10 (27%)  11–15 (19%) / >16 (19%) | | Type of school: Elementary and Middle  Experience of children with T1D: 63%  Number of children with T1D:  0 (37%) / 1 (31%) / 2(21%) / 3 (6%) /4(3%) / 5(1%) |
| Guttu *et al* (2004), USA  To examine the impact of school nurse-to-student ratios on student outcomes | 21 counties, 19 provided school nurse services  No further details presented | | Each county was characterised as having a good nurse-student ratio (1 nurse < 1,000 students) or a fair to poor nurse-student ratio (1 nurse >1,000 students |
| Joshi *et al* (2008), USA  To gather nurses perceptions of the barriers related to diabetes knowledge, communication and management | 43 school nurses recruited (RR not specified)  From across one US state  Sample characteristics: not provided | |  |
| Nabors *et al* (2005), USA  To examine nurses’ perceptions of how to support adolescents with T1D at school | 38 school nurses recruited, surveyed from across 3 states. (34% RR). RN (34%)  Years of experience*:* 6 months-32 years  Mean = 8.6 S.D. 6.3 | | Type of school: Middle (31%) / High (38%)  Both middle and high (31%)  Experience of children with T1D: 87%  Number of children with T1D: ns |
| Wagner and James 2006, USA  The purpose of this study of school counsellors were to explore whether training in diabetes is associated with better knowledge and more helpful attitudes regarding students with diabetes | 132 school counsellors recruited  Attendees at two School Counsellor Association annual meetings (83% & 42% RR)  School Counsellors (100%)  Years of experience: ns  Type of School: Elementary (7%) /Junior / Middle (28%) / High (61%) / Combined (4%) | | Experience of children with T1D:  83% children with diabetes in their schools.  14% did not know if there were children with diabetes in their schools.  Number of children with diabetes  average of 4 students. |
| Schwartz *et al* (2010), USA  To evaluate the experience of children and adolescents with T1D in school by surveying patients, their parents or guardians, and the school personnel directly involved in their care | 28 school personnel recruited. Linked with children from a hospital diabetes centre. 20 schools represented  (RR not specified). School nurses (85%); Dieticians, teachers, & other (15%)    Years of experience: ns  Type of school: Kindergarten to 12th grade | | Experience of children with T1D: 62.9%  Number of children with diabetes:  0(5.9%) / 1–2 (27.5%)  3–4 (41.2%) / 5–10 (13.7%)  >10 (11.8%) |
| Darby 2006, USA  To examine the challenges encountered by school nurses when caring for students receiving CSII therapy | 11 school nurses recruited who cared for students with T1D on pump therapy (85% RR).  Survey of local schools across 3 counties  Type of school:  Elementary (n=5) / Middle (n=4)  Both middle and high (n=1)  Both elementary, middle and high(n=1) | | Experience of children with T1D:  School nurses who cared for students with T1D on pump therapy  Number of children with T1D: Range 1-4  Years of experience: Range 2 months-5 years  RN(n=6), CNP or APN: (n=2) / LPN (n=3) |
