# Supplementary material for: An ongoing struggle: a mixed-method systematic review of interventions, barriers and facilitators to achieving optimal self-care by children and young people with Type 1 Diabetes in educational settings
Source: BMC Pediatr. 2014 Sep 12;14:228. doi: 10.1186/1471-2431-14-228 (PMC4263204; doi:10.1186/1471-2431-14-228)
Supplement: Supplementary file 4 — Additional file 4: Summary of results (Interventions focusing on children and young people with T1D at educational settings).(DOC 54 KB) [file 12887_2014_1206_MOESM4_ESM.doc]

Additional File: A4

Summary of results (Interventions focusing on children and young people with T1D at educational settings)

| **Izquierdo *et al* 2009, USA, RCT – 2 arms,**  **To test the feasibility and effectiveness of telemedicine to improve care of children with T1D in schools** |
| --- |
| HbA1c  HbA1c values decreased in the telemedicine cohort (p<0.02), and the improvement was maintained over the next several months. No significant differences in slopes or within-group slopes were observed after the 6 month point (the beginning of the summer vacation).  Urgent encounters  Urgent visits to the school nurse for diabetes related problems and urgent calls to the diabetes centre decreased significantly over time in the telemedicine cohort, but not in the usual care group (p value not reported).  Hospitalisations and emergency department visits  There were fewer hospitalisations and emergency department visits in the telemedicine intervention group than the usual care group. (not tested)  Diabetes Quality-of-life  Treatment 1 Dimension – assesses the extent to which children experience pain during finger prick or insulin injections, embarrassment about having diabetes, arguments about patient care, and difficulty complying with their diabetes plan. There was significant improvement at 12 months on this dimension for the telemedicine group (p<0.039).  Treatment 2 Dimension – assesses the extent to which children experience difficulty with taking blood glucose tests, taking insulin injections, exercising, tracking carbohydrates / exchanges, wearing their medical alert bracelet, carrying a fast acting carbohydrate, or difficulty eating snacks. There were significant improvements at 6 months for children in the telemedicine group (p=0.017). Usual care showed no significant change during this time but at 12 months the usual care group had improved significantly (p= 0.29 ) and the telemedicine participants remained at levels similar to the 6 month time point.  Generic Quality-of-life  Physical Functioning: Improved in usual care group at 6 months (p=0.14) and was maintained at 12 months  Emotional Functioning: Improved in telemedicine group at 6 months but no further change by 12 months, whereas in the usual care group participants showed an improvement between month 6 and month 12 (p=0.034). |
| **Nguyen *et al* 2008, USA, RCT – 2 arms**  **To determine if school nurse supervision of glucose and insulin-dose adjustment would lead to improvements in Hba1c level in pediatric patients with poorly controlled T1D** |
| HbA1c  At the end of the 3-month study period, the HbA1c level remained unchanged in the control group, but was decreased significantly in the intervention group (p<0.0001) |
| **Engelke *et al* 2008, USA, Before and after study**  **To implement and evaluate a school-based case management program for children with chronic illnesses** |
| Quality-of-life  Baseline QOL overall – 65.48, SD 13.5 / End of year 69.41 SD 11.9. This was reported as a percentage change of 6%.  There was a statistically significant improvement in the treatment barrier subscale (p=0.01, Eta squared =0.19) this was reported as a percentage change of 18.3%.  % Meeting Goals  The degree of achievement for goals set for each child was examined. Nurses were more successful in achieving goals that were under their control.  Percentage of children meeting achievement goal   - Decrease episodes of hypoglycaemia (65%) / Decrease episodes of hyperglycaemia (54%) - HbA1c value of less than 7% (27%) / Teacher/staff complete diabetes management training (100%) |
| **Faro *et al* 2005, USA, Before and after study**  **To conduct periodic diabetes care visits in school, with the goal of promoting optimal management of diabetes for high risk youth** |
| Self-efficacy  No statistically significant differences were seen between pre-intervention and post-intervention mean scores on the Self-Efficacy for Diabetes (SED) Tool. Neither were statistically significant differences seen between pre-intervention and post-intervention self-care practices, parent satisfaction with school care, HbA1c, family knowledge and competence in diabetes management, or frequency of hospitalization or emergency department visits.  Despite the lack of statistically significant outcomes, some encouraging trends were seen. In particular, blood glucose monitoring at home increased, the frequency of insulin administrations at school doubled as need for in intensification of insulin regimens was demonstrated by increased blood glucose monitoring, and insulin adjustments in response to documented need increased.  The researcher felt that had the study been extended for another school year and the sample size been larger, a stronger outcome might have been achieved. |
| **Wdowik *et al* 2000, USA, Controlled trial**  **To develop and evaluate “Control on Camps” for college students with T1D** |
| Knowledge was improved as a direct result of the intervention and was maintained at follow up  Scores in the treatment group increased significantly (p<0.001) from 66.3% (SD 3.4) correct at pre-test to 90.5% (SD 3.4) correct at post test. Treatment group scores at 3 month follow up were 82.1% (SD 3.4) and remained significantly higher than pre-test scores (p<0.001).  HbA1c  A higher percent of treatment group participants (91%) knew their recent HbA1c results than did control group participants (40%), p=0.003.  Within the treatment group, the change in the number of students knowing HbA1c at pre-test versus 3 month follow-up was significant (p=0.005).  Attitudes / Beliefs  The EBHM consists of 10 constructs and at pre-test the mean scores were initially high for both treatment and control groups. The only attitudinal construct hat appeared to change over time was the social influence construct. The mean score for the treatment group increased significantly (p<0.05) from 4.1 (SD 0.1) at pre-test to 4.5 (SD=0.1) at 3 month follow up.  On Campus Support  Question: I feel support on campus for my diabetes: Treatment group participants had significantly more increased scores (76%, p<0.01) than control groups (30%).  Blood Glucose Monitoring  Question: I don’t test my blood sugar as often as I should because I am afraid of what I will find: Improvement for treatment group participants with 38% showing decreased scores, with control group participants had no decreased scores, which was a significant difference. (p<0.05).  Behaviours  In the past week, I tested my blood sugar: Mean scores improved for the treatment group from 4.2 (SD 0.3) at pre-test to 4.5 (SD 0.3) at 3 month follow up (p<0.05) and did not change for the control group.  In the past week I followed my prescribed insulin regime: Mean score remained high for the treatment group, from 4.8 (SD 0.1) at pre-test to 4.9 (SD 0.1) at follow up but declined from pre-test to follow-up for the control group, from 4.9 (SD 0.1) to 4.6 (SD 0.1) p<0.05. |

**Summary of results (Interventions focusing on** school personnel working with children and young people with T1D)

| **Husband *et al* 2001, Canada, RCT – 2 arms**  **To determine of a CD-Rom teaching tool increases teachers’ diabetes knowledge and confidence** |
| --- |
| Knowledge of diabetes  There was no significance in pre-test scores between the control group and the experimental group for total knowledge following CD-Rom mailing. The post test scores increased for both groups but this was not significant  Knowledge of hypoglycaemia  There was no significance in pre-test scores between the control group and the experimental group for hypoglycaemia knowledge following CD-Rom mailing. The post test scores increased for both groups but this was not significant  Confidence  There was no significant difference in confidence pre-test scores between the control and experimental groups. The post-test confidence scores increased significantly in the experimental group only (p < .016). |
| **Siminerio and Koerbel 2000, USA, before and after study**  **To assess diabetes knowledge and needs of school personnel and to determine the effectiveness of the “5 ‘s program”** |
| Knowledge  Overall pre-test scores were 75+11.0; post-test scores were 94+4.1 (mean+SD). Participants showed a statistically significant (p<0.004) improvement in overall knowledge scores.  Answering with correct answers to pre-test questions  1. Sugar is essential for the brain to function: 88%  2. The American Disabilities Act includes children with diabetes: 60%  3. A major concern for the school child with diabetes is the likelihood of developing:79%  4. A sign of high blood sugar in a child with diabetes may be: 53%  5. A low blood sugar requires: 88%  6. A general rule for treatment of low blood sugar: 95%  7. Children with diabetes need to miss more school days: 92%  8. Glucagon is: 64%  9. Schools can ask parents to waive liability: 77%  10. Children with diabetes should be reprimanded if seen eating a candy bar: 75%  Open ended responses  The most frequent concerns in having a child with diabetes in the classroom were.  Having enough knowledge regarding signs and symptoms of high and low blood sugars  Being able to recognize and properly treat hypoglycaemia  The lack of preparedness of other school personnel, e.g. substitutes, aides, bus drivers and coaches |
| **Cunningham and Wodrich 2006 , USA, Analog experiment (allocated)**  **To examine the effect of providing teachers with varying levels of information about T1D** |
| Confidence  Teachers’ mean confidence was similar across no disease information, basic disease information, and basic disease information _ classroom implications levels (11.93 *SD*_2.53, 11.59 *SD* _ 2.71, and 11.26 *SD* _ 3.67, respectively). There was no support for the prospect of enhanced confidence in accommodations based on teachers receiving greater information.  Years of teaching experience did not improve the model, indicating that years of teaching experience is not a significant covariate influencing the relationship between increased information and generation of disease-specific accommodations.  Nine five percent of the sample indicated that they would desire more information and/or assistance about how to better accommodate the learning needs of a student with T1D (there was no difference between the different T1D information levels).  Nine three percent would seek additional information from a school nurse, 90% from the student’s parents, 60% from the student, 58% from an experienced fellow teacher, 53% from the student’s physician, 43% from a special education director or teacher,34% from a school counsellor, and 32% from a school psychologist. |
| **Wodrich 2005, USA, Analog experiment (random assignment)**  **To investigate the effects of disclosing information about T1D with implications for classroom learning and behaviour** |
| Confidence  The more knowledge teachers have about the consequences in the classroom of chronic health conditions the more confident they will be in attributing chronic conditions to behaviour (*chi squared* = 10.0; p = .007; Cramer’s V = .38) |
| **Bullock *et al* 2002 USA, Cohort study**  **To determine if attendance at specific continuing education programs increased competence of school nurses who enrolled and completed the programmes** |
| Ninety one percent reported that the education would enhance their ability to manage students with diabetes |
| **Bachman and Hsueh 2008 USA, Program evaluation**  **To develop and evaluate an online continuing education program to educate school nurses in how to manage care for children with diabetes in schools using current practice principles outlined in *Diabetes Management in the School Setting. A resource guide for School Nurses*** |
| Perceived competence  Those school nurses who had enrolled and completed the course reported a statistically significant higher level of perceived competence (mean = 1.54) than those who had not participated (mean 1.87, p=0.0001) |
