# Supplementary material for: An ongoing struggle: a mixed-method systematic review of interventions, barriers and facilitators to achieving optimal self-care by children and young people with Type 1 Diabetes in educational settings
Source: BMC Pediatr. 2014 Sep 12;14:228. doi: 10.1186/1471-2431-14-228 (PMC4263204; doi:10.1186/1471-2431-14-228)
Supplement: Supplementary file 5 — Additional file 5: Study methods, quality appraisal and summary of results (Stream 2).(DOC 208 KB) [file 12887_2014_1206_MOESM5_ESM.doc]

| **Study / Country / Aims** | **Methods/ Quality appraisal** | | **Summary results** (Only results relating to barriers and facilitators are reported) |
| --- | --- | --- | --- |
| **Children and Parents** | | | |
| Nabors *et al* (2003), USA, To assess children’s and young adolescents perceptions of supportive behaviour by nurses, teachers and friends that allow them to improve their diabetes management at school | | | |
| Design: Interviews & survey  Measure: ID - *“How is School Scale” /* SSI-S  Data analysis: CCA / Descriptive and inferential statistics  Quality criteria met: ABCDEHI | | Substantive themes: Improving staff knowledge / Availability of snacks and supplies / Help with lows  Reminders / Support from other people to help them manage their diabetes at school  Support: Younger children reported needing more support from teachers and nurses | |
| Lehmkuhl and Nabors 2008, USA, To assess children’s perceptions of their satisfaction and support from school nurses, teachers, and friends in their classrooms as well as the types of support they needed from each group | | | |
| Design: Survey - Pilot Study  Measure: Revised How is School Scale / Revised CATIS  Data analysis: Descriptive and inferential statistics  Quality criteria met: ABCEHI | | Support from school nurses (n=45), make sure do insulin injections (13%), BGM(42%), have a snack available (69%), have juice available (73%), help with lows (82%) and have supplies for testing available (80%).  Support from teachers (n=53), make sure do insulin injections (100%), BGM (4%), have a snack available (62%), have juice available (66%), help with lows (30%), have supplies for testing available (28%), help with following meal plan at lunch (17%), help me to recognise when I am beginning to experience a low (58%), send me to the nurse right away if I am low (66%) and let me test when I need to (79%)  Support from others(n=49), help me with BGM (8%), help with following meal plan at lunch (14%), help me to recognise when I am beginning to experience a low (49%), don’t give me snacks (39%), find an adult if I look unwell (45%) and walk me to the nurse if I need to go (63%)  Extra curriculum activities: The support they received from adult leaders during after school activities was between “some” and “ok” (M3.38, SD 1.19)  Support: A regression analysis explained 40% of the variance in HbA1c at 6 months (Mean satisfaction with support ratings, HbA1c at study entry, and the interaction term were significant F(3.19)=11.97, p<0.001).  HbA1c: Children reporting higher levels of satisfaction with support were more likely to have higher HbA1c at 6 months (Beta =2.16, SE=0.92, t=2.35, p<0.05). Children with a higher HbA1c at study entry were apt to have a higher level 6 months later (Beta = 6.92, SE=1.89, t=3.67, p<0.001). The interaction of support and HbA1c was significant (Beta =-0.187, SE=0.60, t=-3.10, p<0.01). When satisfaction ratings were higher and HbA1c was lower at study entry, children were more likely to have a lower HbA1c at 6 months. | |
| Bodas *et al* (2008), Spain, To improve awareness of the needs, within school settings of children and adolescents with T1D based on information provided by the students | | | |
| Design: Survey  Measure: ID  Data analysis: Descriptive statistics  Quality criteria met: ABCEFI | | School nurse: 21% reported having a school nurse (private schools 51%) and 45% felt that there should be a nurse at school.  Glucagon: was available at school 34% and 60% felt that someone school would be willing to inject glucagon.  Support: Younger children (6-10 years) received more support than older children (11-16 years). With only a minority reporting that they had to modify their insulin guideline and/or diminish the glycaemia controls due to the lack of help at school.  BGM: 7% of the teachers help the children with BGM (of those under 10 – 15%).  Insulin: 32% need to inject insulin at school (under 10 years old, 42%, private schools, 49%) and the presence of a nurse (47%). Most (97%) self inject (Mother (2%) / Teachers (1%)  Teachers knowledge: 54% stated that at school they know how to recognize the symptoms of a hypoglycaemia and 49% of PE Teachers able to recognise signs of hypoglycaemia.  Local school policies: 23% reported that they did not have this opportunity to resist an exam after a hypoglycaemia event  Extra curriculum activities: Younger children (6-10 years) reported difficulties in relation to the out-of-school activities  Information: more written information for teachers about the symptoms and steps to be followed in case of a hypoglycaemia, information about the diabetes in general and should have emergency information about the diabetes in their class and in the common areas. Friends should have information about the diabetes and the availability of juices or glucose | |
| Peters *et al* (2008), USA, To examine diabetes-related teacher victimization | | | |
| Design: Survey and review of clinic records  Measure: DSMP  Data analysis: Descriptive and inferential statistics  Quality criteria met: ABCDEFGHI | | Support: Mean score for diabetes related teacher victimisation was 4.35 + 1.16 (range 4-12).  BGM: Whether they put off or delayed checking their blood sugar at school because their teacher might get angry (Sometimes (n=11, 7%, n=4, 2% Often and n=3, 2% always). Whether they avoided checking their blood sugar at school because their teacher might get angry (Sometimes n=8, 5%, n=2, 1% Often, n=2, 1% Always).  Teacher victimisation was associated significantly with decreased dietary adherence as assessed by the DSMP hypoglycaemia sub scale (r=-0.158, p<0.05).  For children aged 8-11 years, teacher victimisation was associated significantly and negatively with DSMP diet subscale (r=-0.256, p<0.05), DSMP insulin adherence subscale (r=-0.343, p<0.01) and DSMP total score (r=-0.305, p<0.01).  For adolescents aged 12 years and above, teacher victimisation was not associated with any variables. | |
| Tang and Ariyawanska 2007, UK, To identify the difficulties that young people with diabetes may encounter while at school | | | |
| Design: Survey  Measure: Open ended questions  Data analysis:  Descriptive statistics and numerical content analysis  Quality criteria met: ABCEFHI | | Self-management skills at school: 64% undertake BGM, 27% administer insulin and 91% take a snack to school 18% reported feeling different to their friends. 55% reported that their friends already knew about their diabetes and 45% would like their friends know especially hypoglycaemia episodes  School based issues:(Medical Room, Toilet) to go for BGM, Inject insulin etc). 45% reported a problem with the teaching staff in relation to needing to BGM, administer insulin or eat a snack during a lesson. Only 1 person reported issues of bullying. | |
| Wang *et al* (2010), Taiwan, To obtain an initial understanding of school –based lived experiences of adolescents with T1D | | | |
| Design: Interviews conducted in Mandarin Chinese  Measure: Semi structured schedule  Data analysis:  A Heideggerian hermeneutic phenomenological approach  Quality criteria met: ABCDEFGHI | | Substantive themes: learning to be master of their disease, learning to find ways to feel comfortable learning not to be different, learning to not let others (especially parents) worry about them | |
| Newbould *et al* (2007), UK, To examine the experiences and concerns of young people and their parents in the management of medication for asthma or diabetes whilst at school | | | |
| Design: Interviews  Measure: Semi structured schedule  Data analysis: Qualitative analytical procedures and numerical content analysis  Quality criteria met: ABCDEFGHI | | Self-management skills: 100% took items related with their condition to school, only 12% injected insulin during school hours  Accessibility of own snacks and supplies: Storage of medication or items related to diabetes; with young person (38%), another room in school (16%), in Classroom (42%), in school Office (4%)  Local school policies: All of the 3 young people who regularly administered insulin during the school day they reported problems with a lack of a private location within the school where they could administer injections.  Parents were unhappy that school policies meant that behaviours encourages within the home to ensure good diabetic control, such as regular snacking and BGM throughout the day, could not be continued at school.  50% of parents did not know whether or not their child’s school have a policy regarding medicine, 31% said yes and 19% said no. | |
| MacArthur 1996, UK, Looked at the practice and attitudes of local children who were taking pre lunch insulin and injections at school | | | |
| Design: Survey  Measure: ID:  Data analysis:  Descriptive statistics and numerical content analysis  Quality criteria met: ABCHI | | Location of lunch time injections: a variety of locations was reported including classroom, toilets, medical nurses room, “Wherever I have my lunch”, “in the dining room”, in a cupboard in the school office” and go home. However they reported being happy with their usual place.  Self-management skills: The majority conducted their own injections  Accessibility of own snacks and supplies: They all looked after their own pen at school  Classmates watching: 12 (80%) stated that they did not mind others seeing them take their insulin. | |
| Clay 2008, USA, To examine problems with medication administration in the school | | | |
| Design: Survey  Measure: ID  Data analysis: Descriptive statistics  Quality criteria met: ABCDEFGHI | | School nurse: 87.5% reported that they had a school nurse  Self-management skills at school: Medicines kept in the nurse’s office (62.5%), Secretary/teachers office / desk (16.7%), in their own bag (15.3%) and other 9.7%). Reminder to take medicines (Teacher 6.9%, no reminding needed 86.1%, someone gets me 1.5%, other 8.3%).  76.4% administer their own medicine (Nurse 18.1%, Teacher/secretary 5.6%, other 8.3%). 49.3% - no one watches when they take their medicine (Person who gives it 40.9%, another person-9.9%). Never miss taking medicine at school (81.9%), Once a year (12.5%), once a month to < once per week (4.2%). | |
| Schwartz *et al* (2010), USA, To evaluate the experience of children and adolescents with T1D in school by surveying patients, their parents or guardians, and the school personnel directly involved in their care. | | | |
| Design: Survey  Measure: ID  Data analysis: Descriptive statistics  Quality criteria met: ABCEH | | Self-management skills at school: Treated differently at school Very Often 4.2%, Often 10.4%, Sometimes 31.2% & Rarely 22.9%. Accused of using diabetes as an excuse Very Often 14.6%, Often 6.2%, Sometimes 16.7% & Rarely 12.5%. Prevented from managing their diabetes (Very Often 8.3%, Often 8.3%, Sometimes 12.5% & Rarely 14.6%).  Felt embarrassed at school experiencing hypoglycaemia or other diabetes related incidents requiring intervention (22.9%) or when they had to check their blood glucose or take medication at school 27.7%, generally embarrassed in front of their classmates (11.4%) and felt embarrassed intentionally by school personnel (11.4%)  Food availability at the canteen: Does the cafeteria have proper food? (Very Often 20.0%, Often 40.0%, Sometimes – 20.0%, Rarely 6.7%). Own lunch to school (Very Often 11.1%, Often 11.1%, Sometimes 13.3%, Rarely 17.8%).  Education and training: Parents felt that school personnel adequately trained to care for their children / manage T1D. (Always 40%, Usually 20.2%, Sometimes 15.6%, Rarely 35.2%).  Support - parent’s perspective: When child misses school (making up missed class work and communication with the child’s health care provider). (Always 53.7%, Usually 26.8%, Sometimes 12.2%, Rarely 7.3%). Adequacy of support provided when their child missed school because of a diabetes-related illness or doctor visit. (Supportive 53.7%, 26.8% usually supportive, 12.2% sometimes supportive and 7.3% not supportive). 30.8% responded that they had to miss work because of the school’s inadequacy to care for their child.  Liability issues: 65% of school personnel expressed concern about the potential liability of caring for these children and adolescents at school.  Skills of School Nurse: 20% felt adequately prepared to assist a child with hypoglycaemia  Availability of school nurses: Adequate numbers of school nurses (20%). School nurse should be available on school premises during the school day if a student with type 1 diabetes is enrolled (76%).  School policies: 31% no policies in their schools and 21.6% were unaware of specific policies.  Interaction with HCP: Communicating with the child’s health care team - often or very often by (25%). | |
| Hema *et al* (2009), USA, To investigate the daily stressors and coping responses of children and adolescents with T1D | | | |
| Design: Self completion diaries 2/3 weeks  Measure: What upset me today was / What I did  Data analysis: Qualitative description  Quality criteria met: ABCDEFHI | | Identified that diabetes related issues caused them stress during the school day Younger (8-12 years) - n=4 (1.52%), Older (13-18 years) - Diabetes Related n=14 (2.78%).  Specifically mentioned a stressor related to diabetes – “I was really low” “… I had to take 2 units of insulin) | |
| Peyrot 2009, Part of the DAWN Youth WebTalk Study, Brazil, Denmark, Germany, Italy Japan, The Netherlands, Spain, USA, To gain understanding of the challenges and issues facing young people with diabetes and those with responsibility | | | |
| Design: Survey  Measure: ID  Data analysis: Descriptive statistics  Quality criteria met: ABCDEFHI | | Role of school teachers:  With regard to areas for improvement, 58% felt that teachers need to be better informed about diabetes and trained how to deal with an emergency diabetes situation.  Food availability at the canteen  55% felt that they would benefit from more healthy food and drink options. | |
| Carroll and Marrero 2006, USA, To explore the perceptions of how diabetes influences adolescents’ perceptions of quality-of-life in general and their relationships with parents, peers, school and their physician | | | |
| Design: Focus Groups  Measure: Open ended qualitative questions  Data analysis: Themes generated  Quality criteria met: ABCDEFGHI | | Substantive themes: Personal perceptions of life living with diabetes, impact on parental and peer relationships and impact on school  School Culture  Missing lessons because of unstable blood glucose levels and leaving the class to do their blood glucose monitoring | |
| Waller *et al* (2005), UK, To seek the views of children and adolescents with T1D and their parents regarding the acceptability and design of a new diabetes education programme | | | |
| Design: Focus Groups (n=8)  Measure: Semi structured schedule  Data analysis: Themes .generated  Quality criteria met: ABCDEFGHI | | Adjusting insulin dose depending on their chosen meal. The Dose Adjustment for Normal Eating program requires greater self-management, children would be expected to have the knowledge and self-esteem to test blood glucose and inject insulin during school time.  Substantive themes were perceived advantages of the DAFNE programme and concerns about the DAFNE programme. Parents were concerned about the commotion in schools (‘Secondary schools are hyper. C is frightened he’s going to get it (syringe) dropped so he won’t take it.’ Mother) and the lack of understanding of diabetes and its management, despite efforts to educate staff (‘J’s teacher didn’t know for two years that he was diabetic even though I’d written a letter.’ Mother).  Location / Missing break / Classmates watching  Children worried about the inconvenience – having to inject ion the middle of the hall, the reactions of peers watching them injecting and the prospect of missing out on break time | |
| Hayes-Bohn *et al* (2004), USA, Diabetes care at school from the perspective of adolescents with T1D and their parents | | | |
| Design: Interviews  Measure: 12 questions, (1 school)  Data analysis: Thematic analysis  Quality criteria met: ABCDEFHI | | Part of a larger study. Findings for parents and adolescents presented together.  Substantive themes: Knowledge/training of school staff, Food Offered/available and School Rules | |
| Wagner *et al* (2006), USA, To investigate the relationships among perceived school experiences, diabetes control and quality-of-life | | | |
| Design: Survey  Measure: DQOLY  ID: Problems & assistance at school  Data analysis:  Descriptive and inferential statistics  Quality criteria met: ABCDEFHI | | Bullying: 21% reported problems with peers regarding their diabetes.  Missing class: 56% reported missing class time for routine, non-emergent, diabetes care.  Food availability at the canteen: 7% - their school cafeteria made carbohydrate content of prepared foods available.  Support others: 57% indicated they saw a school counsellor for a non-medical, diabetes related problem, but only 66% felt that the school counsellor knew enough about diabetes to be helpful.  Role of teacher: Providing a drawer in the classroom for diabetes supplies, reminding to monitor blood glucose or eat a snack, helping with insulin dosage algorithms, and reading books about diabetes. 14% reported that they had experienced problems with regard to diabetes care (testing, snacking, taking insulin, treating lows, etc.) Examples included not allowing a snack in class or delaying in going to the nurse’s office to treat hypoglycaemia.  Role of peers: 31% noted that peers provided help as a diabetes “buddy.” Children reported that buddies recognize hypoglycaemia, alert staff, prompt self-care, buffer teasing, and escort the student to the nurse. 56% reported that their classmates had received diabetes training.  Quality-of-life: Controlling for age and HbA1c, participants who reported trained peers had significantly higher QOL (*M* = 82.8), than those with untrained classmates (*M* = 75.2), *F*(3, 45) = 7.01, *p* < .05.  Location / HbA1c: 56% reported BGM, treating hypoglycaemic episodes, or injecting insulin outside the classroom (e.g. nurse’s office, main office, at locker between classes). Children who reported leaving class for diabetes care had higher HbA1c (*M* = 8.4) than those who indicated they did so in the classroom or were unrestricted (*M* = 7.5), *F*(4, 48) = 17.31, *p* < .001.  Education and training: 72% - was a person at their child’s school trained to handle diabetes emergencies, usually the school nurse (86%). 58% of parents- their child’s school personnel had received training in routine, non-emergent diabetes care. The child’s parents had provided that training 48% of the time. Type of training varied, for example, an informal conversation, review of educational materials, or consultation with a healthcare provider.  Controlling for age and pump status, children whose parents reported school personnel had received training had significantly lower (better) HbA1c (*M* = 7.7) compared with those with untrained school personnel (*M* = 8.4), *F*(3, 41) = 5.12, *p* < 0.05.  Extracurricular activities: Parents reported that their child’s diabetes affected their decisions regarding participation in field trips (29%), extracurricular activities (27%), and after high-school planning (11%). | |
| Amillategui *et al* (2009), Spain, To identify the special needs of children with T1D at primary school taking into account the perceptions reported by parents, children and teachers | | | |
| Design: Survey (postal)  Measure: ID  Data analysis: Descriptive statistics  Quality criteria met: ABCDEFHI | | Self-management skills: 60% (children), 64% (parents) and 65% (teachers) reported that child required BGM during school hours with help need by Teacher (6%), other personnel (2%) and a peer (5%). Between 9%-11% of children needed insulin administration during school hours.  26% (children), 20% (parents), 8% (teachers) reported that there had been one, or more than one, hypoglycaemic episode before or during an exam.  Children’s major concerns included not being able to recognize a hypoglycaemic episode [mean: 3.64 (+1.43)], followed by not being able to administer insulin to themselves [mean: 3.11 (+1.64)]. In contrast, they seemed to be less worried about the necessity of following a diet [mean: 2.29 (+1.41)] or adhering to a strict management timetable [mean: 2.31 (+1.39)], as well as about feeling different from their peers [mean: 2.36 (+1.48)].  Glucagon: 46% (children), 51% (parents), 51% (teachers) no glucagon available at school. 54%(children) would like to have glucagon readily available along with a person who knows how to administer it.  Role of teacher: 18% (children) , 21% (parents), 4% (teachers) thought that the physical education teacher would not be able to recognize a hypoglycaemic episode during these activities. 70% - would like teachers to be better informed about diabetes and 64% to have a better knowledge of the steps they should follow to manage.  Support: Children reported -Teachers (68%), peers (68%), other school staff was lower (19%). Parents reported teachers (71%), peers (80%), other school staff lower (19%)  Local school policies: 39% were unable to resit an exam they had not been able to take because of their disease.  School nurse: Nurse at the school (48%)  Availability of snacks and supplies: Children would like glucose and fruit juices readily available for them (40%).  Role of teacher: 7% reported that they have experienced problems at school when they informed about their child’s diabetes (2% child not accepted into chosen school, 1% (n=1/167) were forced to change school).  Outside school trips: 16% of parents experienced difficulties in getting their school to accept responsibility for the children during day trips. 86% said that their children undertook the same trips as their peers.  Information and resources: 88% (parents), 95% (children), 99% (teachers) felt that written information about T1D is needed. 64% (children), 79% (parents), 81% (teachers) were convinced about the importance of more written information about T1D would improve integration at school.. 100% (teachers) felt that they need information optimal management of emergencies  Concerns of teachers: Possibility that the children could go into a coma at school Not be able to recognize a hypoglycemic episode | |
| Barnard *et al* (2008), UK, To identify key components of quality-of-life and assess the impact of insulin pump therapy on children / adolescents with T1D and their parents | | | |
| Design: Interviews  Measure:  Adapted Schedule for the Evaluation of Individualised Quality-of-life  Data analysis: Descriptive statistics & Thematic analysis  Quality criteria met: ABCDEFGHI | | Support: Children most frequently reported family, friends and school as the discrete life domains that were important for their quality-of-life. | |
| Low *et al* (2005), USA, To explore psychosocial issues related to insulin pump use (continuous subcutaneous insulin infusion (CSII)) in youth aged between 11 and 18 years | | | |
| Design: Interviews  Measure: open ended and informational questions  Data analysis:  Themes generated using a constant comparative method  Quality criteria met: ABCDEFGHI | | Substantive themes: Pump therapy: expectations and benefits, adjustments related to pump therapy, social issues liabilities associated with CSII, wearing an insulin pump and school-related issues | |
| Wilson and Beskine 2007, UK, To examine how children with diabetes are managing their condition in the school setting using pump therapy and multiple daily injections | | | |
| Design: Survey  Measure: Open questions  Data Analysis: Thematic analysis of the context of comments  Quality criteria met: ABCDEH | | Care plan: Child has a school diabetes care plan (64%)  Information and resources: School has written information about child’s diabetes (82%)  Self-management skills: All children perform BGM in school (100%) school staff checking the reading (82%)  Insulin injections during school (40%) overseen for (20%). Help available if needed with BGM or injecting (67%)  Outside school trips: Able to participate in all school trips/outings/clubs (80%)  Bullying: Child bullied/picked on at school because of diabetes (26%)  Support: Younger children (<12 years) required assistance from teacher/classroom assistant, office staff, carer/mother, school nurse, head teacher with BGM during school hours. Some younger children (< 12 years) also needed assistance with insulin injections during school hours usually from parent, teacher/classroom assistant (n=1). Older children (>12 years) conducted their own BGM and insulin injections.  Locations: BGM: classroom (22/73), anywhere (19/73), medical room (15/73), school office (7/73), locker room (1/73), computer room(1/73), toilet/ cloakroom (3/73), head’s office (1/73), side room (1/73), own room (1/73), library/reading room (2/73). Insulin injections: medical room (15/29), school office (7/29), locker room (3/29), toilets/cloakroom (3/29), head’s office (1/29) | |
| Amillategui *et al* (2007), Spain, To identify the special needs of children with T1D in schools from the parents’ point of view and the difficulties experienced with full integration, and to define a series of interventions which may improve the situation | | | |
| Design: Survey  Measure: ID  Data analysis: Descriptive statistics  Quality criteria met: ABCDEFGHI | | School nurse: psychologist and nurse (17%), psychologist (44%) and nurse (5%). Parents felt that a nurse should be available at the school for the children (70%)  Role of school teachers: Teachers were informed about their children’s diabetes (95%), but only 78% of physical education instructors were aware of the children’s condition.  Experienced problems at schools after they had informed the schools about their children’s condition. (3-6 years – 30% / 7-10 years 23% / 11-14 years 16% / >14 years 7%, overall – 17%).  Institutions (62%) and teachers (56%) were considered responsible for these problems. As a result 5% of children were not accepted into the school of the parents’ choice. 8% were forced to change school (3-6 years – 20%).  9% admitted experiencing discriminatory behaviour from the school (3-6 years 23% / 7-10 years – 9% / 11-14 years – 6% / >14 years 6%).  Self-management skills; BGM required during school day (75%), of whom 87% were in the 3 to 6-year-old age group. 9% admitted that they were forced to reduce the number of blood glucose measurements because of lack of cooperation from school staff 3-6 years 18% / 7-10 years –14% / 11-14 years – 4% / >14 years 8%). In 26% of the cases, Insulin injections during school (26%), but only 63% of the children were able to do this by themselves. In 16% of cases, treatment modifications were made because of a lack of cooperation from the school.  Support: The greatest support that their children received at school came from teachers (66%) and peers (63%).  Diabetes knowledge: Parents felt teachers had a basic knowledge about T1D (58%). 34% of parents believed that school personnel would be able to recognize a mild hypoglycaemic episode. In 64% of cases, the children themselves had been able to resolve such an event, with the greatest proportion (80%) being in the 14 years and older age group.  Glucagon: Only 9.8% of children had experienced a serious hypoglycaemic episode at school, but, according to parents, 55% of schools did not have glucagon available. Parents felt that glucagon should be readily available, together with a person who was aware of how to administer it (64%).  Education and training: Parents would like teachers to have a better knowledge of the basic steps that should be taken during a glycaemic emergency (75%), to have a better understanding of the disease (70%)  Food availability at the canteen: 27% of their children ate at school of whom 72% could eat anything from the menu. In all, 63% of parents felt their children’s meals were under control. Parents said that schools were not able, or did not consider it their responsibility, to modify diets to enable children with T1D to eat a school lunch (14%).  Extracurricular activities: 95% of the children engaged in normal physical activities with their peers, although 51% had had at least one hypoglycaemic episode during these activities. In extracurricular activities, 16% of parents experienced difficulties from their school over responsibility of the children during 1-day trips (3-6 years 26%, 7-10 years 27%, 11-14 years 10%, >14 years 7%), falling to 34% when trips were extended over several days.  Peer support: 61% of their children’s peers were sympathetic to a child with diabetes, 12% had experienced some form of verbal abuse or mocking (3-6 years 3%, 7-10 years 7%, 11-14 years 15%, >14 years 18%). | |
| Jacquez *et al* (2008), USA, To investigate parent reports of the diabetes care support their children receive in school, their concerns about diabetes management in school, and their knowledge about diabetes management in school, and their knowledge of federal laws that protect children with diabetes | | | |
| Design: Survey  Measure: ID  Data analysis: Descriptive and inferential statistics  Quality criteria met: ABCDEFGI | | School care plan: 45% of children did not have a written care plan.  School nurse: 45% did not have a nurse at their school.  Glucagon: 49% reported that their school had a glucagon kit.  Location: 54% of children were allowed to perform BGM in the classroom and only 50% of children were allowed to perform BGM in special places. Only 21% of children were allowed to administer insulin in the classroom and only 54% of children allowed administer insulin in special places.  Snacking: 84% reported that the child was allowed extra snacks when needed.  Leaving the class: 81% reported that the child was allowed access to bathroom when needed. | |
| Pinelli *et al* (2011), Italy, To determine how Italian parents and school personnel of 8-13 year old children with T1D manage during school hours, including insulin administration, management of hypoglycaemia, and glucagon use | | | |
| Design: Survey  Measure: ID  Data analysis: Descriptive statistics  Quality criteria met: ABCDEFI | | Self-management skills: Insulin injection done during school time (54.2%). Person responsible for administration of insulin at school (Child 20%, Parent/relative 20%, nurse 3.6%, teacher 2.7%, other school workers 0.9%). Blood glucose test done during school time (58.6%).Treatment of hypoglycaemia during school hours (Carbohydrate 66.9%, Call to parents 47.1%, Self-management 9.6%, Call to emergency services 2.9%, other 3.7%). At least one hypoglycaemic event during school hours (63.6%), the event had been managed well (85.7%) and that a blood glucose test had been done (79.3%). Treatment of hyperglycaemia during school hours (Request of parents advice 64.6%, self-management 22.4%, insulin administration 9.3%, correction with pump 3.7%).  Glucagon: 40.9% of schools had a refrigerator to store glucagon  Understanding of teachers: Parents reported difficulties encountered with school staff in the daily management of diabetes (Practical difficulties of good diabetes management, checks, insulin treatment, hypoglycaemia – 26.3%, underestimation of the impact of diabetes on life by teachers – 24.6%, poor knowledge of illness and therapy – 22.8%, generic attitude of school personnel to avoid responsibility – 15.8%, and the school staff’s fears – 12.3% and refusal to allow self-management of children – 3.5%.  Extra curricular activities: Parents’ assistance and presence during sport and extra curricular activities (54%) | |
| Yu *et al* (2000), USA, To characterize the academic and social experiences of children with diabetes | | | |
| Design: Survey  Measure: ID interview schedule  Data analysis: Descriptive statistics  Quality criteria met: ABCDEFGI | | Role of teacher: Who knows about diabetes – Classmates (97%), Teachers (100%), Administrators (87%) - Early Onset Diabetes. Classmates (100%), Teachers (97%) and Administrators (91%) - Late Onset Diabetes  Accommodation for diabetes 65% - diet related (52%), Medical maintenance (16%), Other (13%) - Early Onset Diabetes. Accommodation for diabetes (80%) – diet related (77%), Medical maintenance (14%), Other (9%) – late Onset Diabetes | |
| Hellems and Clarke 2007, USA, To determine which school personnel currently assist students with insulin administration and management of hypoglycaemia and to determine whether these students are being cared for in a safe manner | | | |
| Design: Survey  Measure: ID  Data analysis: Descriptive analysis  Quality criteria met: ABCDEGHI | | School nurse: School nurse assigned to their child’s school (95%) - with 69% reporting that this was full time.  Location BGM: 49% reported that their children were permitted to check their own blood glucose in the classroom (elementary school – 41%, middle school – 28%, high school – 74%).  Self-management skills: 89% reported that their child required insulin administration at school during the previous year, 79% injected their own insulin. Of children in elementary school, 41% injected their own insulin doses. Of children in high school, 74% injected their own insulin doses.  75% reported that their child had experienced low blood glucose requiring treatment while at school in the previous year. Most episodes were treated with fast acting carbohydrate. One instance of severe hypoglycaemia reported that required glucagon administration.  Kindergarten to 5^th^ grade  Person responsible for child’s diabetes care **during school day**: Medical Personnel (88%), Teachers and administrators (64%), Other school personnel (21%), Parent (16%) / **during after school activities**: Medical Personnel (26%), Teachers and administrators (40%), Other school personnel (2%), Parent (71%), No one (2%) / helps **with BGM**: Medical Personnel (84%), Teachers and administrators (40%), Other school personnel (2%), Parent (19%), No one (3%) / with **insulin administration**: Medical Personnel (74%), Teachers and administrators (29%), Other school personnel (2%), Parent (24%), No one (7%) / **episodes of hypoglycaemia**: Medical Personnel (88%), Teachers and administrators (53%), Other school personnel (14%), Parent (24%), No one (2%)  Middle school – 6^th^- 8^th^ grade  Person responsible for child’s diabetes care **during school day:** Medical Personnel (97%), Teachers and administrators (43%), Other school personnel (30%), Parent (15%) / **during after school activities:** Medical Personnel (25%), Teachers and administrators (37%), Other school personnel (10%), Parent (52%), No one (18%) / helps **with BGM:** Medical Personnel (85%), Teachers and administrators (12%), Other school personnel (0%), Parent (30%), No one (15%) / **insulin administration:** Medical Personnel (53%), Teachers and administrators (8%), Other school personnel (0%), Parent (32%), No one (22%) / **episodes of hypoglycaemia**: Medical Personnel (95%), Teachers and administrators (27%), Other school personnel (17%), Parent (23%), No one (5%)  High school - 9^th^ - 12^th^ grade  Person responsible for child’s diabetes care **during school day:** Medical Personnel (81%), Teachers and administrators (25%), Other school personnel (12%), Parent (6%), No one (9%) / **during after school activities**: Medical Personnel (6%), Teachers and administrators (16%), Other school personnel (22%), Parent (25%), No one (46%) / helps **with BGM**: Medical Personnel (33%), Teachers and administrators (6%), Other school personnel (0%), Parent (13%), No one (54%) / **insulin administration:** Medical Personnel (12%), Teachers and administrators (1%), Other school personnel (0%), Parent (15%), No one (75%) / **episodes of hypoglycaemia**: Medical Personnel (75%), Teachers and administrators (27%), Other school personnel (10%), Parent (19%), No one (18%) | |
| Lewis *et al* (2003), USA, To identify obstacles to good control of diabetes in the school setting and document the level of support available in various school districts serving the clinic patient population | | | |
| Design: Survey  Measure: ID  Data analysis: Descriptive statistics  Quality criteria met: ABCEI | | Extra curriculum activities: 20% of parents reported that their child was not allowed to participate in all school activities without restriction. Many parents stated that their child was not allowed to go on school field trips unless accompanied by a parent or a school nurse, and some parents stated that their child was not allowed to play sports such as football.  Role of teachers: 6.2% of parents stated either that they did not know if teachers or specific child’s teachers were aware of their child’s diabetes.  The most frequent issues specified by parents concerning their level of satisfaction with the management of their child’s diabetes during school hours were better staff training on diabetes management, better communication between parents and schools, healthier school lunches, and a daily on-site school nurse. | |
| Lin *et al* (2008), Taiwan, To explore the essential structure of mothers’ life experience when helping their first-to-third grade children with T1D made adjustments at school | | | |
| Design: Semi structured interviews  Measure: Interview schedule  Data analysis: Colaizzi’s six-step method approach  Quality criteria met: ABCDEFGHI | | Six major themes emerged: Worrying about the child’s safety, creating a safe environment, building the child’s self-care ability, improving academic achievement, assisting with peer relationships and normalizing the child’s life | |
| **Students** | | | |
| Ramchandani *et al* 2000, USA, To assess changes in diabetes management and control that occurs in the transition from high school to attending college away from home. | | | |
| Design: Survey  Measure: ID  Data analysis: Descriptive and inferential statistics  Quality criteria met: ABCDEFHI | | Findings from open ended questions not reported.  Self-management skills: 35.7% blood glucose control was better in college, 33.3% worse & 26.2% not changed. (4.8% did not know). 30.6% of clinicians rated that the metabolic control of the students as being worse in college, 38.9% as not changing and 30.6% as having improved.  71% more difficult to manage their diabetes in college than in high school, 24% easier, 5% same. Diabetes was perceived to be significantly more difficult to manage in college than in high school (p=0.002). Neither an increase in the frequency of SMBG nor an increase in the number of daily insulin injections was associated with perceived increased difficulty of diabetes management.  The reasons selected for any type of change (positive or negative) on college student’s diabetes control were: diet (n=36), exercise (n=34), frequency of SMBG (n=25), increased responsibility (n=24), irregular schedule (n=21), fear of hypoglycaemia (n=17), alcohol use (n=16), no parental involvement (n=13), contact with healthcare provider (n=11).  HbA1c: There was no significant change in control between high school and college | |
| Wdowik 1997, USA, To identify factors that affect the ability and motivation of college students to engage in appropriate self-care behaviours for successful management of diabetes | | | |
| Design: Focus groups and telephone interviews  Measure: Structured interview schedule  Data analysis: Chart of pertinent issues and listing the responses  Quality criteria met: ABCDEFHI | | Self-management skills: The five most salient barriers to successful diabetes management cited were; scheduling and time management difficulties; stress; hypoglycaemic reactions; diet management constraints & inadequate finances  Psycho-social issues were also identified as barriers to diabetes management these included: inconvenience of diabetes management; motivators to managing diabetes, social support issues. | |
| Wilson 2010, UK, To explore the experiences of young people managing their diabetes at college of university | | | |
| Design: Interviews  Measure: Semi structured schedule  Data analysis: Thematic data analysis  Quality criteria met: ABCEFGHI | | Thematic data analysis identified four main  themes: balancing diabetes and further education; adverse diabetes management strategies, reduced participation in social events; and transition to an adult diabetes clinics. | |
| Wdowik *et al* (2001), USA, To determine relationships between constructs of the Expanded Health Belief Model and to identify characteristics of college studiers who successfully manage their diabetes | | | |
| Design: Survey  Measures*:* ID - Diabetes College Scale / Expanded Health Belief Model  Data analysis: Descriptive, inferential and analytical statistics  Quality criteria met: ABCDEFGHI | | HbA1c: Only 45.8% provided a self reported recent HbA1c.  Self-management skills: Planning snacks, exercising and testing blood sugar were reported between sometimes and usually.  Self management attitudes: Survey responses on attitude constructs indicated that participants had good intentions to engage in self-care behaviours. However, intention alone was not enough to result in optimal behaviours, as indicated by the infrequency of exercise reported. Notably, students’ emotions were negative predictors of exercise, such that the more they disliked exercise or felt stressed, out of control, or unhappy, the less likely they were to participate.  Intention and emotional response were strong predictors of exercise, whereas health importance and intention were predictive of testing blood sugar. Situational factors and emotional response were substantial barriers to optimal diabetes --self-care.  In this study, the attitude constructs most predictive of good diabetes management behaviours included Intention and Health Importance, whereas barriers to achieving appropriate outcomes were identified as Situational Factors and Emotional Response. Thus, even students with positive attitudes and good intentions may be unable to engage in desired self-care behaviours if significant barriers or negative emotions are present. | |
| Balfe 2007a, 2007b, Balfe and Jackson 2007. Balfe 2009a, 2009b, UK, To explore the narratives of practice of young university students with T1D | | | |
| Design: Interviews and follow up interviews 6 months later  Research diaries for 2 weeks  Measure: Semi-structured interview schedule  Data analysis: Thematic analysis  Quality criteria met:  Balfe 2007a. ABCDEFHI  Balfe 2007b. ABCEFGHI  Balfe and Jackson 2007. ABCDEFGHI  Balfe 2009a: ABCDEFGHI  Balfe 2009b: ABCDEFGHI | | Balfe (2007a): Alcohol: Discussion centred around the themes of reasons for drinking riskily, risk anxieties (short term and long term), managing risk and changing attitudes to risk  Many respondents (especially first years) engaged in alcohol consumption practices that were risky for their diabetes control in order to perform identities as normal young students.  Younger interviewees mainly engaged in alcohol consumption practices that were risky for their diabetes control in public spaces where they felt that there would be a risk to their identities as normal young people if they did not engage in these practices.  There was evidence that as the respondents experienced transitions within university their attitudes towards the risks of drinking changed and, in many cases, students’ drinking decreased substantially after their first year.  Balfe (2007b): Healthy eating and exercise: Discussion centres around the themes of moral practices – concerns about eating healthily and exercising, abut ‘engaging in the ‘right’ practices, temporalities – concerned about exercising and eating healthily so as to minimise diabetes’ ability to affect their future identifies.  Routine – the university environment itself could deleteriously make an impact upon interviews abilities to engage in moral disciplinary practices, even for those who wanted to do so. Weight management narratives – using disciplinary regimes to manage weight  Balfe and Jackson (2007): Technologies defined as insulin pens, insulin pumps and blood testing equipment. Discussion centres around the themes of the use of technology, disadvantages and factors influencing the use of technology.  Technologies increased respondents’ social and spatial flexibility, providing them with the means to fit their diabetes more easily into their student lifestyles.  Balfe (2009a): Being normal: The main finding was that respondents attempted to be ‘normal’ via engagement with particular body projects: student bodies, toned bodies, and healthy bodies. If individuals cannot balance their body projects, they get ‘identity damage’.  Balfe (2009b): Self-management skills: Five themes were identified: Routine benefits, routines at university, adjustment (achieving a balance between self-care routines and student practices)  Glitching (Glitching describes the involuntary collapse of an entire diabetes self-care routine over an extended period) and crashing (Crashers tended to ignore their self-care routines or be overwhelmed by them).  In summary, students with diabetes can experience significant difficulties with their self-care routines in university, but can learn to overcome these with time and experience. | |
| Geddes *et al* (2006), UK, To examine the clinical characteristics of, and diabetes management provided for, young people with T1D in tertiary education | | | |
| Design: Retrospective survey  Measure: Case notes  Data analysis: Descriptive and inferential statistics  Quality criteria met: ABCDEFGH | | Diabetes management: Year of referral to local diabetes clinic at hospital was not consistent. Only 10 students (18%) were not using a basal bolus regimen by the time they left university. Smoking and alcohol consumption of students seldom reviewed. At the time of initial assessment the mean HbA1c as 8.8% (2.0%). By the end of their attendance at university this was unchanged [8.7% (1.6%), P= 0.77]. Prevalence of retinopathy was 10.9% at the beginning of the observation but b y the end of the observation period this had risen to 14.5%. The frequency of home blood glucose monitoring had not been adequately documented in 13 (24%) of the 55 patients. The frequency of testing in the remainder was variable, ranging from six to eight times per day to once per week. The frequency of mild hypoglycaemia had not been documented | |
| Eaton *et al* (2001), UK, To contact students with diabetes at the University of Leeds to ascertain their, alcohol, smoking and exercise habits and to explore their views on diabetes and factors which influence their ability to maintain glycaemic control | | | |
| Design: Interviews  Measure: Semi structured schedule  Data analysis: Themes generated  Quality criteria met: ABC | | Self-management skills: 22 (55%) reported that having diabetes interferes with being a student.  Two main themes were identified; the effects of having diabetes on students' lifestyles (with three sub themes of alcohol, exercise and student budget) and facilities and services | |
| Ravert 2009, USA, To examine the use of nine common alcohol management strategies among college undergraduates with diabetes in order to determine which strategies predicted alcohol consumption and consequences | | | |
| Design: Survey  Measure: ID  Data analysis: Descriptive and inferential statistics  Quality criteria met: ABCDEFI | | Alcohol: A majority of respondents (68.0%) reported alcohol use in the previous month, with 41.8% consuming five or more drinks in one sitting during the previous 2 weeks.  Reported to usually use the following strategies: Eat before and/or during drinking (77.9%); Keep track of how many drinks you were having ( 65.5%); Determine, in advance, not to exceed a set number of drinks (40.1%); Avoid drinking games (38.7%); Have a friend let you know when you’ve had enough (30.7%); Alternate non-alcoholic with alcoholic beverages (29.4%); Pace your drinks to 1 or fewer per hour (29.6%); Choose not to drink alcohol (34.0%); Drink an alcohol look-alike (non-alcoholic beer, punch etc. (10.0% )  No significant correlation was found between age and overall mean management strategy use. However, two individual strategies, rely on a friend and keep track, were negatively correlated with age (r = -0.13, P = 0.019, and r = -0.11, P = 0.047, respectively), and therefore more common among younger students. In contrast, the strategies pace drinks and avoid drinking games were more common among older students (r = 0.11, P = 0.047, r = 0.21, P < .001, respectively).  High alcohol management strategy use in the past year was associated with fewer heavy drinking episodes and fewer alcohol related consequences. Two strategies, avoiding drinking games and pacing one’s drinking, were especially strong predictors of reduced consumption and consequences, and were more common among older students. | |
| Miller-Hagan and Janas 2002, USA, To explore how college students with diabetes perceive and manage alcohol consumption | | | |
| Design: Interviews  Measure: Semi structured schedule  Data analysis: Constant comparative method  Quality criteria met: ABCDEFI | | Alcohol: Three drinking related perceptions were especially salient among the participants in this study: students with diabetes can drink if they are careful, drinking is the primary social activity at this university, the peer pressure to drink is strong  Students had varied responses to the social environment and pressure to drink. Three distinct practices emerged: non drinking, experimenting with drinking, and drinking within limits.  Analysis for students’ drinking practices revealed six categories of strategies that students used in attempts to restrict or limit their alcohol consumption: to avoid or diffuse peer pressure; limit the frequency of going out to parties and bars; develop personal rules for the amount of alcohol consumed; check or monitor blood glucose levels; “cover” the alcohol with insulin and/or food & drink with trusted friends | |
| **School Personnel** | | | |
| Amillategui *et al* (2009), Spain, To identify the special needs of children with T1D at primary school taking into account the perceptions reported by parents, children and teachers  (for further details see Amillategui *et al* 2009 under children and parents section) | | | |
| Greenhalgh 1997, UK, To assess school teachers knowledge of insulin dependent diabetes in school children aged 5 to 16 years | | | |
| Design: Survey  Measure: AQ- diabetes knowledge  Data analysis: Descriptive and inferential statistics  Quality criteria met: ABCDE | | Diabetes knowledge: Adequate knowledge of diabetes (35%) / (40% Primary and 38.3% secondary, 27% Secondary without science and PE). Primary school teachers scored significantly higher marks (40%) than secondary school teachers (38.3%); (teachers who taught science and PE were excluded).  Sources of information: Parents (64%). Secondary school other sources: radio, television, other school staff, teaching literature, newspapers and magazines.  DKA: Manage symptoms of DKA (37.6%).  Policies: Detention was an appropriate punishment for misbehaviour by a child with diabetes (53% Secondary and 48% Primary). Children with diabetes should not be late for a meal 37.5% secondary and 25% primary)  Outside school Trips: 100% felt that all children with diabetes should be allowed to go on holiday with the school | |
| Bowen 1996, UK, To discover whether the teachers had the training to cope with the health related emergencies which, may arise with children who have special needs | | | |
| Design: Survey  Measure: ID – competence  Data analysis: Descriptive statistics  Quality criteria met: ABCDEFGHI | | Hyperglycaemia: Recognise signs (70%) / Hypoglycaemia: Recognise signs (60%)  Competence: Competent to cope with emergencies which arise with blood sugar levels (63%).  Information about diabetes: More information and advice (86%) | |
| Gormanous *et al* (2002), USA, To determine the levels of knowledge about diabetes mellitus among Arkansas public elementary school teachers | | | |
| Design: Survey  Measure: ID – diabetes knowledge  Data analysis: Descriptive and inferential statistics  Quality criteria met: ABCDEHI | | Training: 19% had never received in-service training or prior education on diabetes.  Information about diabetes: More information (79%)  Diabetes knowledge: Teachers who had previous experience of diabetes were more significantly likely to correctly define hypoglycaemia (65% vs 45%, p<0.05), and know correct treatment (37% vs 13%, p<0.05).  Teachers with family and friends with diabetes were significantly more likely to correctly identify symptoms of diabetes (49% vs 29%, p<0.05), identify symptoms of hypoglycaemia (60% vs 38%, p<0.05), select appropriate treatment for low blood sugar (21% vs 12%, p<0.05) than those with no personal exposure to diabetes. | |
| Alnasir and Skerman 2004, Bahrain, To study awareness about common health problems in Bahrain  Latif Almasir 2003, Bahrain, To assess the Bahrani school teachers’ knowledge of diabetes | | | |
| Design: Survey  Measure: ID – diabetes knowledge  Data analysis: Descriptive and inferential statistics  Quality criteria met: ABCDEF{Alnasir, 2004 #32} / ABCDEFI{Latif Alnasir, 2003 #161} | | Diabetes knowledge: Possible range 1-10, mean 5.34 (SD 2.13), median 5.5  The level of diabetes knowledge was significantly better in females, science teachers, those who did not drink alcohol, those with an ill family member and those who had unsatisfactory perception about general health. | |
| Tahirovic 2007, Bosnia and Herzegovina, To investigate how far physical education teachers from elementary school understand diabetes and are trained in its management and in the treatment of diabetes emergencies according to their understanding | | | |
| Design: Survey  Measure: AQ  Data analysis: Descriptive and inferential statistics  Quality criteria met: ABCDEFH | | Training: Physical education teachers whose schools were attended by at least 1 pupils suffering from T1D were significantly more likely to been taught about T1D (Group 1 – 39%, Group 2 – 20%, x^2^ =5.04, p=0.02)  Physical education teachers whose schools there were pupils suffering from T1D were significantly more likely be interested in learning about T1D (Group 1 – 89%, Group 2 – 98%, x^2^=4.18, p=0.04).  Hypoglycaemia: Physical education teachers whose schools were attended by at least 1 pupils suffering from T1D were significantly more likely to know about hypoglycaemia. x^2^=4.178, p=0.040.  Policies: Schools where there were pupils suffering from T1D were significantly more likely be allowed to take glucose or food during lessons? (Group 1 – 82%, Group 2 – 53%, x^2^=10.32, p=0.001)  Schools where there were pupils suffering from T1D were significantly more likely be allowed to measure his/her blood glucose levels during lessons? (Group 1: 71%, Group 2 – 31%, x^2^=7.58, p<0.05) | |
| MacArthur 1996, UK, Looked at the practice and attitudes of local children who were taking pre lunch insulin and injections at school | | | |
| Design: Survey  Measure: ID:  Data analysis:  Descriptive statistics and numerical content analysis  Quality criteria met: ABCHI | | Location: Certain area to do the insulin injection. (yes, n= 6, no, n = 5) reasons for yes mainly concerned the student’s privacy, safety and cleanliness. The school staff did not think that the children needed to be supervised whilst doing injections at school.  91% (n=10) of school staff reported that pupils should look after their own pens | |
| Boden *et al* (2011), UK, To examine the concerns of primary school staff working with children with T1D and their parents, and to relate these views to the views of health care professionals working with school personnel | | | |
| Design: Interviews  Measure: Semi structured interview schedule  Data analysis: Qualitative approach  Quality criteria met: ABCDEFGHI | | Primary school staff expressed a range of concerns about injecting and blood glucose testing, the ability of children to mishandle their condition, and corresponding reactions of parents to school decisions on health-based matters. | |
| Nabors *et al* (2008), USA, To assess special education and regular education teachers’ perceptions of their knowledge about and confidence in meeting the academic and social needs of children with chronic medical conditions (including diabetes) | | | |
| Design: Survey  Measure: ID - Perceptions of knowledge and confidence  Data analysis: Descriptive and inferential statistics  Quality criteria met: ABCEFGHI | | Diabetes knowledge: 22.1% of teachers indicated being very well informed (rating of 5 or 6) regarding diabetes. There was no difference in knowledge of regular education teachers and special education teachers.  Confidence: 42.5% of teachers (regular education teachers and special education teachers) indicated being very confident (rating of 5 or 6) in meeting the academic needs of students’ diabetes. 43.7% of teachers indicated being very confident (rating of 5 or 6) in meeting the social needs of students with diabetes. There was no difference in confidence in meeting the social needs of students of regular education teachers and special education teachers. | |
| Lewis *et al* (2003), USA, To identify obstacles to good control of diabetes in the school setting and document the level of support available in various school districts serving the clinic patient population | | | |
| Design: Survey  Measure: ID – 25 items  Data analysis: Descriptive statistics  Quality criteria met: ABCEI | | Policies: No policy pertaining to diabetes management (9%: n=6) / Students with diabetes did not have activity restrictions (95%)  Blood glucose monitoring: Not allowed to perform BGM while at school (3% n=2)  Availability of supplies: Did not have refrigerators with a local available for the storage of glucagon, insulin or syringes (20%). Food and beverages containing glucose were not readily available (3% : n=2)  Training: Did not have staff trained in diabetes management 17% : n=11) | |
| Rickabaugh and Salterelli 1999, USA, To explore the attitudes and reported behaviours of participants concerning diabetes and exercise guidelines | | | |
| Design: Survey  Measure: ID – Diabetes and exercise knowledge and attitudes  Data analysis: Descriptive and inferential statistics (non parametric)  Quality criteria met: ABCDEGHI | | Knowledge: Parents (36.3%) performed noticeably better on knowledge items than children with T1D and physical education teachers (23.1%). PE teachers were also very unsure about the optimal HbA1c range for children with T1D (PE Teachers 100%, Parents 32%). PE teachers were unsure about what exercise limitations exists for children with T1D (PE = 47%, Parents = 86%). PE teachers were also more uncertain than parents on the effects of exercise on long term blood glucose levels (PE teachers = 56%, Parents 86%).  Attitudes: Children with T1D and their parents displayed similar attitudes toward diabetes and exercise guidelines, while PE teachers appeared to be less familiar with these procedures and indicated that they were “unsure” on many responses. | |
| Chmiel-Perzynska *et al* (2008), Poland, To evaluate the knowledge of primary school teachers in the Lubelskie Province, Poland, about hypoglycaemia and to determine educational needs necessary to ensure that children with diabetes are properly dealt with by their teachers | | | |
| Design: Survey  Measure: ID - diabetes knowledge  Data analysis: Descriptive statistics  Quality criteria met: ABCDE | | Blood glucose monitoring: Know what a glucose meter is (98%) but only 46% declared being able to operate it.  Hypoglycaemia: Recognise signs (71.3%) / Did not know what blood glucose levels are indicative of hypoglycaemia (76.9%) / Did not know how they could help a hypoglycaemic child (42.3%).  Glucagon: Did not know what it was used for (43.3%) / Knew when glucagon should be given (23.1%) / They knew how to give glucagon (15.4%)  Diabetes knowledge: 92% considered their knowledge sufficient and only one in five expressed a willingness to participate in a free training on diabetes. | |
| **School Healthcare Personnel** | | | |
| Fisher 2006, USA, To measure school nurses’ perceived self-efficacy in providing diabetes care in education to children | | | |
| Design: Survey  Measure: SEDE  Data analysis: Descriptive and inferential statistics  Quality criteria met: ABCDEGHI | | Support: 62.9% participated in the care of children with T1D & supervised blood glucose meter testing.  Education and training: 94.3% reported they had up-to-date diabetes reference materials in their offices,  Attended a conference on diabetes during the past year (35.7%)  Self-efficacy: Mean SEDE score 36.30 – moderately confident. There was a significant relationship between higher self-efficacy scores and having a diabetes curriculum. However, only nine school nurses reported having a diabetes curriculum.  Significant findings in this study were positive relationships between self-efficacy and 3 variables:(a) participating in the care of children with diabetes, (b) having type 1 diabetes children in the school system, and (c) supervising children with blood glucose meter testing. Regression analysis of self-efficacy on these demographic variables revealed *R* of .42 (*R* squared .20), indicating that 20% of the variance in self-efficacy was explained by these factors (*p =*0.01). | |
| Guttu *et al* (2004), USA, To examine the impact of school nurse-to-student ratios on student outcomes in a 21-county region with a range of school nurse-to-student ratios | | | |
| Design: Survey  Measure: % students with T1D known to school nurse  Data analysis: Descriptive and inferential statistics  Quality criteria met: ABCDEI | | Availability of school nurse: There was no difference in the percentage of students with diabetes between counties and nurse-to-student ratios. A significant correlation existed between increased presence of school nurses and services provided to children with diabetes (r=0.52, p=0.000). | |
| Joshi *et al* (2008), USA, To gather school nurses perceptions of the barriers related to diabetes knowledge, communication and management | | | |
| Design: Survey  Measure: ID  Data analysis: Descriptive statistics and numerical content analysis of open ended questions  Quality criteria met: ABCEH | | Diabetes knowledge: Low to average perception (29%).  Source of diabetes information: Internet (79%) / Professional books and magazines (42%).  Barriers to acquiring new information: Time constraints (37%), Lack of access to education / regular updates and inadequate training (28%).  Barrier to increasing students’ adherence to medical regimen: Improper food habits (limited food choice in cafeteria, availability of snacks with high carbohydrates (40%).  Problems faced in helping students cope with T1D: Easy access to vending machine s (36%), inadequate physical and parent orders (27%), students do not want to be recognised as different to their peers (53%), enhance education of students (42%).  Ways to promote better support of children diet schedules should be modified (32%), improve communication with provider and parents (30%), timely availability of supplies (21%). | |
| Nabors *et al* (2005), USA, To examine nurses’ perceptions of how to support adolescents with T1D at school | | | |
| Design: Survey  Measure: ID  Data analysis: Descriptive and inferential statistics.  Content-Coding Process based on grounded theory  Quality criteria met: ABCDEHI | | Experience of diabetes: Adequate (54%) or high (30%).  School policy: 92% had developed written health care plans for a child with diabetes. Nurses who worked for more years as a ‘‘school nurse’’ were more apt to report that they had developed care plans for youth with diabetes (r =0.22, p=0.025).  Extra curriculum activities: 73% did not believe that after-school activities should be included in written health care plans. Participants with more years of experience as a school nurse were more likely to state that care plans should address after-school activities than those with less experience (r =0.25, p =0.012).  Diabetes knowledge: School staff needed to improve their knowledge about diabetes.  Support from school staff: 98% of nurses agreed that adolescents with diabetes needed more support at school.  Nurses who felt knowledgeable about diabetes were more likely to report that adolescents with diabetes needed more support at school than nurses who did not feel they were knowledgeable (r =0.30, p =0.002).  Facilitators to adherence at school: Four themes emerged: improving communication among everyone who could potentially help the adolescent at school, educating nurses and school staff, improving parental involvement in school planning and improving support for adolescents.  Barriers to adherence at school: Five themes emerged: issues for teens, issues for school staff, communication, food management, education | |
| Wagner and James 2006, USA, The purpose of this study of school counsellors were to explore whether training in diabetes is associated with better knowledge and more helpful attitudes regarding students with diabetes | | | |
| Design: Survey  Measure: ID-awareness of diabetes related issues / DAS Version 3 / TDKT  Data analysis: Descriptive and inferential statistics  Quality criteria met: ABCDEFGHI | | 87% indicated that they had received no specific training about diabetes, yet 40% had reportedly worked directly with students with diabetes.  Knowledge: Scores on the TDKT - mean =10.3 (SD=3.4), indicating a basic understanding of diabetes according to criteria established by the scale’s authors. Knowledge deficits were demonstrated by 15% of the sample, basic understanding by 57%, and scores indicative of being an effective support for children with diabetes by 28%. Respondents who reported diabetes training had higher TDKT scores (mean = 13.04) than those who did not (mean = 10.51), F(3,90) = 8.62, p<0.01.  Attitudes: Scores on the DAS - with mean =3.9 (SD =0.5). Respondents who reported diabetes training had higher DAS scores (mean =4.27) than those who did not (mean = 3.96), F(3,91) =6.55, p<0.05.  Awareness: Many school counsellors endorsed items which would hinder their ability to serve students with diabetes. Specifically, counsellors were neutral or agreed with unhelpful statements such as ‘‘diabetes restricts extracurricular activities’’ (35% did not disagree) and ‘‘the most appropriate place for children with diabetes to test blood sugar is in the nurse’s office’’ (87% did not disagree). | |
| Schwartz *et al* (2010), USA, To evaluate the experience of children and adolescents with T1D in school by surveying patients, their parents or guardians, and the school personnel directly involved in their care. (for further details see Schwartz *et al* 2010{Schwartz, 2010 #241}, under children and parents section) | | | |
| Darby 2006{Darby, 2006 #89}, USA, To examine the challenges encountered by school nurses when caring for students receiving CSII therapy | | | |
| Design: Interviews  Method: Semi structured schedule  Data analysis: Phenomenological analysis with the process of intuiting and describing at the centre of this analysis  Quality criteria met: ABCDEFHI | | Eight themes evolved from the data. These were: Feeling scared, developing trust, knowing your students, working with Children’s Hospital, teaching and learning, talking the talk, dealing with pump problems and calculating challenges  The nurses' responses indicated that they were "scared" when first caring for students with continuous subcutaneous insulin infusion therapy. However, they were able to work through their fear by using their resources and gaining more knowledge and hands-on experience with insulin pumps. The data also revealed that school nurses who were able to learn the language of continuous subcutaneous insulin infusion therapy and successfully deal with pump problems developed trusting and knowing relationships with students, teachers, and parents | |

**Quality criteria key:**

1. Clear statement of the aims of the study.
2. Adequate description of the context for the study.
3. Clear specification of research design and its appropriateness for the research aims.
4. Reporting of clear details of the sample and method of recruitment/sampling.
5. Clear description of data collection.
6. Clear description data analysis provided.
7. Attempts made to establish rigour of data analysis.
8. Discussion of ethical issues / approval details.
9. Inclusion of sufficient original data to support interpretations and conclusions
