# Supplementary material for: An ongoing struggle: a mixed-method systematic review of interventions, barriers and facilitators to achieving optimal self-care by children and young people with Type 1 Diabetes in educational settings
Source: BMC Pediatr. 2014 Sep 12;14:228. doi: 10.1186/1471-2431-14-228 (PMC4263204; doi:10.1186/1471-2431-14-228)
Supplement: Supplementary file 6 — Additional file 6: Overarching synthesis matrix of entire dataset mapped against best practice.(DOC 80 KB) [file 12887_2014_1206_MOESM6_ESM.doc]

**Additional file 6. Overarching synthesis matrix of entire dataset mapped against best practice principles.**

| **School/College/University T1D guidance** | **Experiences of and attitudes to achieving optimal TID management in educational settings** | | **Interventions to promote optimal T1D management in educational settings** | | |
| --- | --- | --- | --- | --- | --- |
| **Best practice principles** | **Barriers** | **Facilitators** | **RCTs** | | **Non RCTs** |
| **Assembling school health care plans** | | | | | |
| Individualised Diabetes Medical management Plan (DMMP) should be agreed by the parent/guardian, school, and the student’s diabetes team [12]and updated on a regular basis [11]  DMMP developed by the student’s personal diabetes HCT with input from the parent/guardian [18] along with specific IHP / EMP [19] | Generic school-based policies that that don’t take into account the needs of students with T1D[49]C&P; [50]P  Students do not have written care plan[46]P; [47]P; [48]P | School nurses felt that individual healthcare plans that set out the components of diabetes care in school as important[51]HCP | None identified | | A school based case management approach involved the development of an IHP and Emergency Action plan with specific goal setting as appropriate for each pupil showed a significant improvement in the treatment barriers subscale for diabetes QOL (p=0.01) [36] |
| **Checking blood glucose during the school day** | | | | | |
| Support for blood glucose monitoring and guidance on the interpretation of blood glucose results and any subsequent action [8, 9]. School nurse is the most appropriate person in school to provide care for a student with T1D [19]  School nurse and back-up trained school personnel who can check blood glucose and ketones and administer insulin, glucagon, and other medications as indicated by the student’s DMMP [18]. Permission for student to see school nurse and other trained SPl upon request [18] | Lack of assistance with blood glucose monitoring.  6-13 years[65]C,P;  5-12 years [48]C,P | Assistance with blood glucose monitoring and the interpretation of results especially for younger children  <10 years [60]C; [61]P;  < 12 years [46]P  13 years[67]C,P | Blood glucose reading checked for those students with poorly controlled T1D by school nurse showed significant decreases in HbA1c (p<0.0001) at 3MFU for children aged 10-17 years (mean 13) [32] | | A school based diabetes care program involved a review of school blood glucose readings by a PNP with the pupil and the school showed no significant differences HbA1c for children under 11 years of age. [38] |
| **Accessibility and storage of supplies and snacks** | | | | | |
| Parents and, where appropriate, school nurses and other carers should have access to glucagon in an emergency, especially when there is a high risk of severe hypoglycaemia [17] School personnel be offered education on the administration of glucagon [17]. Parents/guardians should supply the school with a glucagon emergency kit [19, 68] and school nurse and/or TDP must know where it is stored and have access to it at all times [14, 19, 68].  Provision of fridge space for insulin {11}and correct storage of other supplies including glucagon where necessary [11, 18]  Give permission for student to eat whenever required [9,11, 18]. Schools may need to make special arrangements if the school has staggered lunch times [9]  Permission for self-sufficient and capable students to carry equipment, supplies, medication, and snacks; to perform diabetes management tasks [18]    Diabetes supplies and equipment (e.g glucogel, glucose drinks and some complex carbohydrate to treat hypoglycaemic episodes) should be accessible to the student at all times {17, 18] | Lack of remedies and trained personnel for treatment of severe hypoglycaemia[60]C; [47]P; [65]C  Not being allowed to eat snacks when needed[49]C&P [50]P  The timing of school lunches was also a commonly reported problem [50]P.  Teachers did not realize that students with T1D should not be late for lunch [71]SP  Students prevented from having easy access to supplies and equipment at all times[51]HCP; [56]HCP; [83]P | Availability of Glucagon for the treatment of severe hypoglycaemia and personnel trained in its administration[60]C [65]C,  Permission to be able to eat a snack during the day as required[52]C;[83]P  Diabetes supplies and equipment to stored at appropriate locations and accessible at all times[59]C;[54]SP;[50]C | None identified | | None identified |
| **Administering insulin during the school day** | | | | | |
| Support of administration of insulin including treatment changes and a suitable location [8, 9]  Assignment of diabetes care tasks, must take account of State laws that may be relevant in determining which tasks are performed by trained diabetes personnel [19] | Lack of support for insulin administration[65]p;[60]c [52]C especially for younger children 8-12 years [106]C;  6-10 years [60]C  < 12 years [46]P,  <10 years [61]P;  8-13 years [64]P | Support with administration for younger children  ns [63]C,P;  6-10 years [60]C;  8-13 years[64]P;  < 12 years [46]P | Supervision of insulin administration for those students with poorly controlled T1D by school nurse showed significant decreases in HbA1c (p<0.0001) at 3MFU for children aged 10-17 years (mean 13) [32] | | None identified |
| **Providing suitable locations for checking blood glucose and administering insulin during the school day** | | | | | |
| A location that provides privacy during blood glucose monitoring and insulin administration [18]. Permission for the student to check his or her blood glucose level and take appropriate action to treat hypoglycaemia in the classroom or anywhere the student is in conjunction with a school activity, if indicated in the student’s DMMP [18, 19]. Provide and clean and safe environment [11]. Suitable, private location to manage injections and check blood glucose [9] | Lack of a private locations for insulin administration and blood glucose monitoring  [52]C; [50]C; [47]P | Assess to a private location for insulin administration and blood glucose monitoring[47]P;[46]P;[58]SC; [57]SP | None identified | | None identified |
| **Accessibility of and participation in physical education in schools** | | | | | |
| Allow students to manage their T1D according to their chosen management form and to take part in the full range of school activities[12]  Students with T1D should participate fully in PE classes and team or individual sports [19]  Staff in charge of PE or other physical activity sessions should be aware of the need for them to have glucose tablets or a sugary drink to hand [9] | No strategies in place to enable participation in sport at school [70]C; [74]C; [59]C; [73]P; [64]P | Strategies in place so that students can participate in sport at school[50]P; [65]P] | None identified | | None identified |
| PE teachers and sports coaches must be able to recognize the symptoms of hypoglycemia and be prepared to call for help with a hypoglycemia emergency[19] |  |  |  | |  |
| **Food and dietary management** | | | | | |
| Carb content of foods should be provided to  the parents/guardian and the student [19]  Information on serving size and caloric, carbohydrate, and fat content of foods served in the school [18] | Unhealthy choices available in school canteen[49]C&P; [56]HCP; [48]C,P.  A lack of information available about foods served, portion sizes and carbohydrate content of foods available[58]SC | Ensuring snacks and appropriate food and drink are available [51]HCP; [72]C,P [65]C; [73]P  Nutritional labeling on food choices[49]C&P | None identified | | No significant changes in HbA1c were reported when students and school nurses were provided with school menus that included carbohydrate servings for all food items listed as part of the school based diabetes care program [38] |
| **Planning for special events, field trips, and extracurricular activities** | | | | | |
| Students with T1D must not be excluded from day or residential visits[12]. Information should be readily available from the PDSN on the inclusion of students with T1D on school trips [11]. | Lack of support during after school activities[58]SC; [61]P | Parents and school nurses felt that school care plans that include strategies for after school care[59]C, [51]HCP. | None identified | | None identified |
| Full participation in all field trips, with coverage provided by trained diabetes personnel [18] . The school nurse or TDP should accompany the student with T1D on fieldtrips [19] . Parental attendance at field trips should never be a prerequisite for participation [19]  School nurse or TDP should be available during school-sponsored extracurricular activities that take place outside of school hours [19] | Problems with pupil being allowed to participate in school day or extended trips[65]P; [67]C,P,SP; [60]C; [46]P; [73] | Specific policies in place in relation to school trips[50]P |  | |  |
| **Flexible accommodation for school exams and tests** | | | | | |
| Alternative times and arrangements for academic exams if the student is experiencing hypoglycaemia or hyperglycaemia [19] | Students unable to have opportunity to re take exams if they are experiencing hypoglycaemia or hyperglycaemia[60]C; [67]C |  | None identified | | None identified |
| **Dealing with emotional and social issues** | | | | | |
| The student’s personal diabetes HCT and school health team must be aware of emotional and behavioral issues and refer students with T1D and their families for counseling and support as needed [19] | School nurses felt that students being made to feel “different” from their peers acts as a barrier towards self-care [56]HCP; [53]C | Support from peers considered important [50]P; [58]SC;.[59]C; [74]C  Students felt that peers being informed about their condition and having information about diabetes was important [60]C; [59]C; [52]C | None identified | | None identified |
| **Assisting the student with performing diabetes care tasks** | | | | | |
| The school nurse and/or TDP should assist with insulin administration in accordance with the student’s health care plans and education plans [19] | Lack of adequate daily support from school nurse[51]HCP; [59]C | Availability of a school nurse every day during school hours [65]C; [60]C [73]P ;especially for younger children (age ns:[59]  Support from school counsellor for non medical diabetes related problems[58]SC | Supervision of insulin administration and blood glucose reading checked for those students with poorly controlled T1D by school nurse showed significant decreases in HbA1c (p<0.0001) at 3MFU.  Mean age 13 years. Range 10–17 years[32] | | None identified |
| **School healthcare personnel: knowledge, skills and education** | | | | | |
| Opportunities for the appropriate level of ongoing training and diabetes education for the school nurse[18] | Lack of time to access education and regular updates)[56]HCP  Students felt knowledge could be improved [49]C&P; [59]C.  Nurses reported low levels of diabetes knowledge[56]HCP and moderate levels of confidence [80]HCP  Difficulties in getting to grips with CSII therapy[81]HCP; [82]HCP  Nurses are perceived to be inadequately prepared to assist a student with hypoglycaemia[48]HCP | Access to up to date information[80]HCP  Diabetes knowledge and skills updated on a regular basis[80]HCP | None identified | | A continuing education programme seeking to increase the competence of school nurses showed significant improvements in perceived confidence (p=0.0001) [42]  School nurses felt that an continuing education programme enhanced their ability to manage students with diabetes [41] |
| **Diabetes education and training of school nurses and school personnel** | | | | | |
| All SP should be taught an overview about diabetes and how to recognize and respond to an emergency situation [10, 11, 17, 18]  All SP who have responsibility for the student with T1D throughout the school day should be receive training for recognition and management of hypoglycaemia [16] and emergency situations [18, 19]  All SP designated as trained diabetes personnel who will perform or assist the student with diabetes care tasks when allowed by State law should receive specific training [18, 19]  It is important that when staff agree to administer blood glucose tests or insulin injections, they should be trained by an appropriate HCP [7, 8, 10, 16, 18]  Staff members need an appropriate level of diabetes education, and this should be relevant to activities that take place on the premises as well as those associated with participation in school trips and camps [87] | School staffs fear of liability[54]SP  Lack of understanding when students need to leave the classroom to manage their diabetes[70]C; [58]SC  Students not allowed a snack in class[47]P;[58]SC; [52]C  Lack of diabetes knowledge of school personnel[51]HCP; [67]C,P; [59]C; [49]C&P; [117]P  Students and parents felt school personnel would benefit from written information about T1D [67]C,P [60]C  Teachers un-willing participate in diabetes training[85]SP | Teachers prepared to support a student with diabetes on a daily basis[105]C;[74]C; [54]SP.  Awareness of needs of students with T1D in their classroom[49]C&P; [59]C  The majority of information about diabetes received from parents [71]SP  Training in diabetes management for school staff seen as beneficial [50]P;[49]C&P;[70]C; [73]p; [58]SC  Especially in how to deal with an emergency diabetes situation[65]C; [72]C,P | A CD Rom teaching tool containing basic diabetes information showed a significant increase in confidence (p<0.016) but no changes knowledge [34] | | The ‘5 C- of diabetes’ lecture based program for school personnel showed significant improvements in knowledge  (p<0.004) [37]  To investigate the effects of disclosing information about T1D with implications for classroom learning and behaviour  Teachers’ mean confidence was similar across no disease information, basic disease information, and basic disease information _ classroom implications levels (11.93 *SD*_2.53, 11.59 *SD* _ 2.71, and 11.26 *SD* _ 3.67, respectively[39]  The more knowledge teachers have about the consequences in the classroom of chronic health conditions the more confident they will be in attributing chronic conditions to behaviour (p=007)[40] |
| **Recognizing and treating hypoglycaemia and hyperglycemia** | | | | | |
| Awareness by school staff of the signs of Hyperglycaemia [8-16]  Hyperglycemia needs to be recognized and treated in accordance with the student’s DMMP [19]  All SP who have responsibility for the student with T1D should receive a copy of the Hyperglycemia Emergency Care Plan and be prepared to recognize and respond to the signs and symptoms of hyperglycemia [19] | Diabetes HCPs biggest concern was about the ability of teachers to spot the onset of hypoglycaemia and react quickly[54]HCP |  | None identified | None identified | |
| **Communication between school health personnel and diabetes healthcare providers** | | | | | |
| None identified | Poor communication with health care providers and the school nurse[48]P; [56]HCP | Having regular appointments with healthcare providers and written communication between the health care providers and the school nurse regarding management needs for school and increased information exchange between the two was seen as something that would be beneficial[51]HCP, [54]SP | School nurses exchanging graphical and tabular blood glucose measurement information (telemedicine) with the diabetes nurse showed significant improvements for diabetes QOL on the treatment barriers subscale at 12 mths: (p=0.039); significant improvements on the treatment adherence subscale(at 6 mths: p=0.017 which was maintained at 12 mths and .significant improvement for HbA1cat 6 mths [(p <0.02)](#_ENREF_123) [33] | | A school based diabetes care program which sought to strengthen collaboration between school health personnel and the children’s diabetes center staff to resolve diabetes-related school problems and enhance diabetes management showed no significant differences HbA1c. Although a trend towards increased blood glucose monitoring at home was observed and the frequency of insulin administrations at school doubled [38] |
| **Self-care and management at college/university** | | | | | |
| None identified | Lack of perceived routine with little or no time to engage in self-care practices[88]C; [89]C; [35]C; [90]C; [44]c  Poor adherence to dietary recommendations[35]C; [109]C.  Inadequate finances[35]C; [89]C  Alcohol use[92]C; [89]C; [95]C  In frequent contact with health care provider[90]C | Ability to juggle all aspects of diabetes and student life[111]C; [108]C;[89]C.  Strategies in place when engaging in alcohol consumption[95]C; [91]C; [35]C; [92]C; [88]C |  | | The intervention “Control on Campus” showed significant improvement in knowledge (p<0.001) which was maintained at 3MFU (p<0.001). Significant increase in numbers of students knowing their HbA1c results (p=0.003), 3MFU (p=0.005) and a significant increase in testing BGM in the past week (p<0.05), 3MFU (p=0.005) [43] |

Key –Information obtained from C-Children/Young Person, P–Parent, C&P-Children and Parent HCP–Health Care Professional, SC, School Counselor, PE – Physical Education, PNP – Pediatric Nurse Practitioner, SP- School Personnel, TDP – Trained Diabetes Personnel

Quality of Evidence – **Red – Low/Very Low,** **Amber – Moderate**, **Green – High**
