# Supplementary material for: An ongoing struggle: a mixed-method systematic review of interventions, barriers and facilitators to achieving optimal self-care by children and young people with Type 1 Diabetes in educational settings
Source: BMC Pediatr. 2014 Sep 12;14:228. doi: 10.1186/1471-2431-14-228 (PMC4263204; doi:10.1186/1471-2431-14-228)
Supplement: Supplementary file 7 — Authors’ original file for figure 1 [file 12887_2014_1206_MOESM7_ESM.pdf]

REVIEW QUESTION

What is known about the barriers to, and facilitators of, providing optimal care and management for children and young people with T1D within educational settings?

↓

SCREENING EXERCISE

1. Systematic and exhaustive searches to identify all relevant research
2. Retrieval, screening and classification of full reports

↓

Agreement on key questions, review scope and focus amongst co-applicants

Focus for in-depth review prioritized by study type

Stream 1:
'Intervention studies'

Stream 2:
'Non intervention studies'

IN-DEPTH REVIEW

Conducted within study type

Stream 1:
'Intervention studies'

1. Application of inclusion criteria
2. Data extracted from studies to describe characteristics and assess methodological quality
3. Data extracted on study findings
4. Findings synthesized to answer sub-question:

Which interventions are effective for optimising the care and management of children and young people with T1D in educational settings?

Stream 2:
'Non intervention studies'

1. Application of inclusion criteria
2. Data extracted from studies to describe characteristics and assess methodological quality
3. Data extracted on study findings
4. Findings synthesized to answer sub-questions:

What are the attitudes and experiences of children and young people with T1D and those involved with their care and management in educational settings?

What are the barriers and facilitators to achieving optimal T1D management in educational settings?

Stream 3:
IN-DEPTH REVIEW
Conducted across study type

Synthesis across study types to answer sub question:
'To what extent do interventions address the barriers identified build upon the facilitators for providing optimal care and management of children and young people with T1D in educational settings?'
