# Supplementary figures and images for: An ongoing struggle: a mixed-method systematic review of interventions, barriers and facilitators to achieving optimal self-care by children and young people with Type 1 Diabetes in educational settings
Source: BMC Pediatr. 2014 Sep 12;14:228. doi: 10.1186/1471-2431-14-228 (PMC4263204; doi:10.1186/1471-2431-14-228)

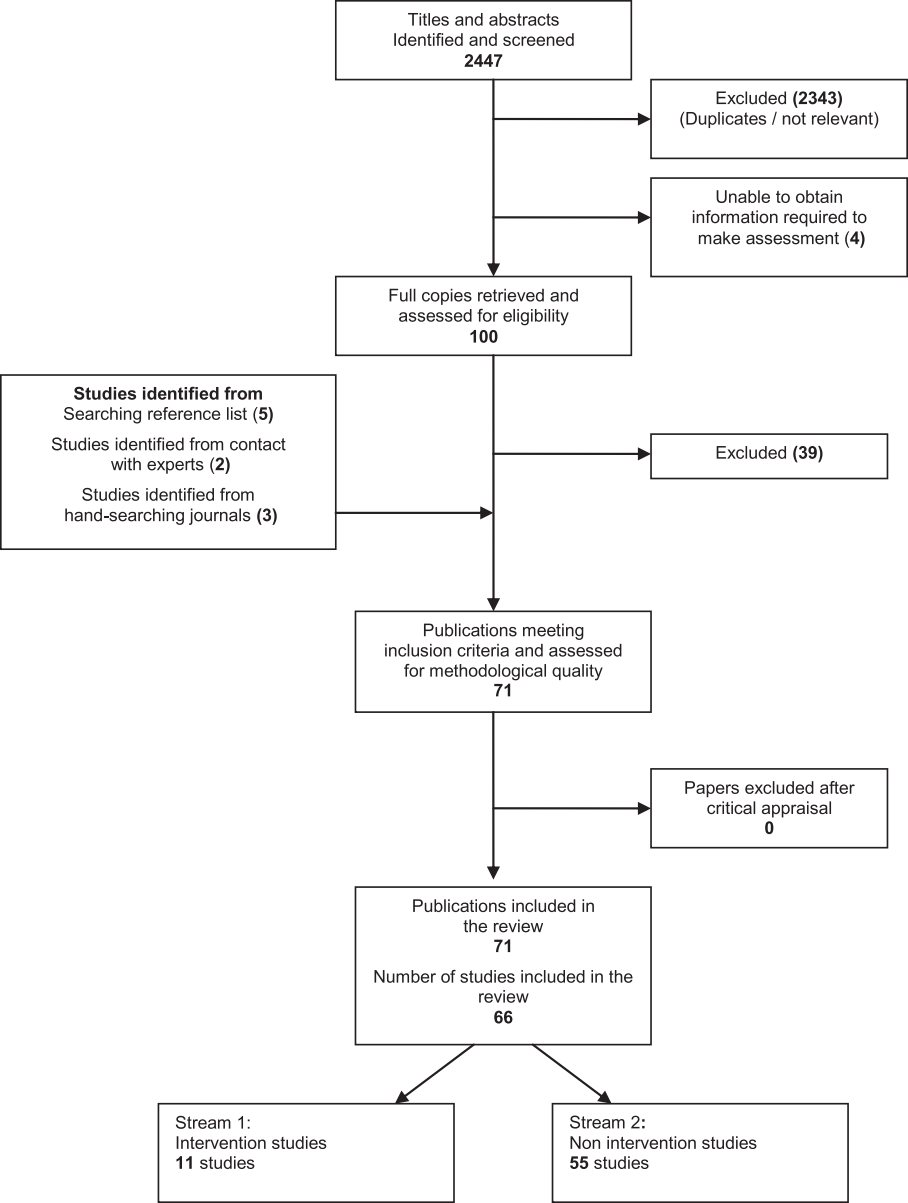

Supplement: Supplementary file 8 — Authors’ original file for figure 2 [file 12887_2014_1206_MOESM8_ESM.pdf]
