# Supplementary material for: An ongoing struggle: a mixed-method systematic review of interventions, barriers and facilitators to achieving optimal self-care by children and young people with Type 1 Diabetes in educational settings
Source: BMC Pediatr. 2014 Sep 12;14:228. doi: 10.1186/1471-2431-14-228 (PMC4263204; doi:10.1186/1471-2431-14-228)
Supplement: Supplementary file 9 — Authors’ original file for figure 3 [file 12887_2014_1206_MOESM9_ESM.pdf]

**High confidence in qualitative evidence**	A review finding drawn from generally well-conducted studies with few methodological limitations and showing high levels of coherence	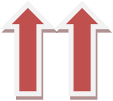
**Moderate confidence in qualitative evidence**	A review finding where there are concerns regarding *either* the methodological limitations of the studies *or* the coherence of the review finding	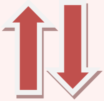
**Low confidence in qualitative evidence**	A review finding based on studies with important methodological limitations *and* where there are concerns regarding the coherence of the review finding	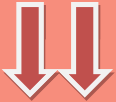
**Coherence**	*The extent to which a clear pattern can be identified across individual study data.* This pattern could include, for example:<br>* circumstances where the review finding is consistent across multiple contexts *or*<br>* where the review finding incorporates explanations for any variations across individual studies<br><br>Coherence may be further strengthened if the individual studies contributing to the finding are drawn from a wide range of settings	
