# Supplementary material for: An ongoing struggle: a mixed-method systematic review of interventions, barriers and facilitators to achieving optimal self-care by children and young people with Type 1 Diabetes in educational settings
Source: BMC Pediatr. 2014 Sep 12;14:228. doi: 10.1186/1471-2431-14-228 (PMC4263204; doi:10.1186/1471-2431-14-228)
Supplement: Supplementary file 10 — Authors’ original file for figure 4 [file 12887_2014_1206_MOESM10_ESM.doc]

Full copies retrieved and

assessed for eligibility

**100**

Publications meeting

inclusion criteria and assessed for methodological quality

**71**

Publications included in

the review

**71**

Number of studies included in the review

**66**

Excluded **(39)**

Excluded **(2343)**

(Duplicates / not relevant)

Unable to obtain

information required to

make assessment (**4)**

**Studies identified from**

Searching reference list (**5)**

Studies identified from contact

with experts (**2)**

Studies identified from

hand-searching journals **(3)**

Titles and abstracts

Identified and screened

**2447**

Papers excluded after critical appraisal

**0**

Stream 1:

Intervention studies

**11** studies

Stream 2**:**

Non intervention studies

**55** studies
